# The treatment of Qibai Pingfei Capsule on chronic obstructive pulmonary disease may be mediated by Th17/Treg balance and gut-lung axis microbiota

**DOI:** 10.1186/s12967-022-03481-w

**Published:** 2022-06-21

**Authors:** Yu Jia, Tiantian He, Di Wu, Jiabing Tong, Jie Zhu, Zegeng Li, Jingcheng Dong

**Affiliations:** 1 College of Integrated Chinese and Western Medicine, Anhui University of Chinese Medicine, No.1, Qianjiang Road, Hefei, Anhui China; 2 Institutes of Integrative Medicine, Fudan University, Shanghai, China; 3 Institute of Traditional Chinese Medicine Prevention and Control on Respiratory Disease, Anhui Academy of Chinese Medicine, No. 117, Meishan Road, Hefei, Anhui China; 4 Department of Respiratory Medicine, First Affiliated Hospital of Anhui University of Traditional Chinese Medicine, Meishan Road, Hefei, Anhui China

**Keywords:** COPD, Th17/Treg, Intestinal microbiota, Pulmonary microbiota, Gut-lung axis, QBPF

## Abstract

**Background:**

Chronic obstructive pulmonary disease (COPD), a prevalent, progressive respiratory disease, has become the third leading cause of death globally. Increasing evidence suggests that intestinal and pulmonary microbiota dysbiosis is associated with COPD. Researchers have shown that T helper (Th) 17/regulatory T (Treg) imbalance is involved in COPD. Qibai Pingfei Capsule (QBPF) is a traditional Chinese medicine used to treat COPD clinically in China. However, the effects of QBPF intervention on the Th17/Treg balance and microbiota in the gut and lung are still poorly understood.

**Methods:**

This study divided the rats into three groups (n = 8): control, model, and QBPF group. After establishing the model of COPD for four weeks and administering of QBPF for two weeks, Th17 cells, Treg cells, their associated cytokines, transcription factors, and intestinal and pulmonary microbiota of rats were analyzed. Furthermore, the correlations between intestinal and pulmonary microbiota and between bacterial genera and pulmonary function and immune function were measured.

**Results:**

The results revealed that QBPF could improve pulmonary function and contribute to the new balance of Th17/Treg in COPD rats. Meanwhile, QBPF treatment could regulate the composition of intestinal and pulmonary microbiota and improve community structure in COPD rats, suppressing the relative abundance of *Coprococcus_2*, *Prevotella_9*, and *Blautia* in the gut and *Mycoplasma* in the lung, but accumulating the relative abundance of *Prevotellaceae_UCG_003* in the gut and *Rikenellaceae_RC9_gut_group* in the lung. Additionally, gut–lung axis was confirmed by the significant correlations between the intestinal and pulmonary microbiota. Functional analysis of microbiota showed amino acid metabolism was altered in COPD rats in the gut and lung. Spearman correlation analysis further enriched the relationship between the microbiota in the gut and lung and pulmonary function and immune function in COPD model rats.

**Conclusions:**

Our study indicated that the therapeutic effects of QBPF may be achieved by maintaining the immune cell balance and regulating the gut-lung axis microbiota, providing references to explore the potential biomarkers of COPD and the possible mechanism of QBPF to treat COPD.

**Supplementary Information:**

The online version contains supplementary material available at 10.1186/s12967-022-03481-w.

## Background

COPD is a common respiratory disease associated with notable morbidity and mortality, causing a heavy economic burden on society and families [1]. Studies have shown that COPD development is related to the T lymphocyte mediating inflammatory immune response and immune imbalance [2]. The Th17 cells and Treg cells, as subtypes of CD4^+^ T lymphocytes, play critical roles in the pathogenic process of COPD [3, 4]. Cervilha et al. demonstrated the crucial role of Th17/Treg imbalance in worsening the pulmonary inflammation in a COPD mice model [5]. Therefore, furthering the understanding of immune imbalance in the pathogenesis of COPD is beneficial for disease treatment.

With the recent advances in multiple omics techniques, a knowledge of the communities of commensal microorganisms within the human body was improved [6], and the correlation between the respiratory and gastrointestinal tract has been gradually discovered [7]. In modern medicine, the gut–lung axis (GLA) theory uses the immune system and microbial flora, which colonize the intestine and lung, as a link hub to form a two-way axis that connects the intestines and lungs [8]. The microbiota of the gut has been the most extensively investigated and has a profound impact on host physiology, metabolism, immune function, and nutrition [9]. Gut microbiota dysbiosis plays a causal effect in influencing the severity of cigarette smoke-induced COPD pathogenesis [10]. Faecal microbiota transplantation experiments further indicate that altered gut microbiota in COPD patients accelerated COPD progression in mice [11]. Lungs were once considered to be a sterile environment [12]. However, the microbiota of the lung is now recognized as a cornerstone in the physiopathology of numerous respiratory diseases [13]. Changes in the pulmonary microbiota are directly associated with the onset of respiratory infections, including pneumonia and influenza [14]. Studies have shown that the microbial composition of the lower respiratory tract of patients with chronic respiratory diseases is different from that of healthy people [15, 16]. Therefore, the intestinal and pulmonary microbiota may be a new perspective to investigate COPD's pathogenesis and treatment mechanism.

Human microbiota has been shown to interact with the human immune system [17]. Bacterial interactions may alter the host's immune and inflammatory response [18]. Lactobacillus murinus A pulmonary strain (CNCM I-5314) increases the presence of lung Th17 cells and a Treg cell subset, suggesting that strains found in the lung may shape local T cells in mice [19]. Intestinal microbiota could maintain the balance of local immune response [20, 21] and is also involved in the immune response process of various respiratory diseases [22], such as immune dysfunction in recipient mice after faecal transplantation of COPD patients [23].

Traditional Chinese medicine treatment has advantages in alleviating symptoms, reducing the frequency of acute exacerbation, and improving the quality of life in COPD [24–26]. QBPF, as the hospital preparation of the first affiliated hospital of Anhui University of Traditional Chinese Medicine (Patent No.: ZL 2010 10573274.1), has long been used in the clinical treatment of COPD for more than ten years. QBPF can improve the pulmonary function of COPD patients [27], reduce the body mass index, dyspnea grade and traditional Chinese medicine syndrome score of COPD patients [28, 29], and ulteriorly improve the quality of life of patients [30]. Pulmonary function in COPD rats was also improved by QBPF [31, 32]. Animal experiments showed that QBPF could regulate T lymphocyte subsets, increase CD3^+^ and CD4^+^ T cells, decrease CD8^+^ T cells [33], and increase the proportion of CD4^+^CD25^+^Foxp3^+^ Treg cells in COPD rats [34]. Clinical trials indicated that QBPF combined with Montelukast could down-regulate Th17 expression and up-regulate CD4^+^CD25^+^Foxp3^+^ Treg expression in acute exacerbation of COPD (AECOPD) patients [35]. These experiments provided the basis for the regulation of immune function by QBPF, but the exact effect of QBPF on Th17/Treg balance was needed to be further explained. In this experiment, we intervened the rat model of COPD by Chinese medicine QBPF. We investigated whether the improvement effect of QBPF on COPD was achieved by maintaining the immune cell balance and regulating the gut-lung axis microbiota, providing new ideas and directions to explore the potential biomarkers of COPD and the possible mechanism of QBPF to treat COPD.

## Methods

### Establishment of COPD model

Twenty-four Specific pathogen-free (SPF)-grade Sprague–Dawley (SD) strain of rats with a weight of (200 ± 20) g were purchased from the Experimental Animal Center of Hangzhou Medical College (SCXK 20190002; Hangzhou, China). All rats ate and drank freely, changed bedding materials every other day, controlled temperature at (22 ± 1) ℃, relative humidity at 60%, and light of 12 h light/12 h dark cycle in an animal breeding room. The treatment of rats followed the relevant provisions of the Regulations on the Management of Laboratory Animals. Experimental Animal Ethics Committee of Anhui University of Chinese Medicine reviewed and approved all experiments (identification number: AHUCM-rats-2021021).

A total of 24 rats were randomly divided into three groups after adaptive feeding for one week: control group, model group, and QBPF group (n = 8 per group). To establish the disease model of COPD [36], rats of the model and QBPF group were forced to swim for 30 min every day in a constant temperature [(43 ± 1) ℃] water tank (homemade by the First Affiliated Hospital of Anhui University of Chinese Medicine). Subsequently, we put rats in a 1 m^3^ whole-body inhalation chamber (homemade by the First Affiliated Hospital of Anhui University of Chinese Medicine) filled with cigarette smoke (Dujiang brand cigarette, tar content: 10 mg, nicotine content: 0.8 mg, carbon monoxide content: 13 mg, China Tobacco Anhui Industrial Co., Ltd) for 20 cigarettes/day, 1 h/day. Then put the rats in the constant hypoxia oxygen device (Shanghai Tawang Intelligent Technology Co., Ltd), 7 h/day, using an automatic oxygen meter to control the oxygen concentration to (10 ± 0.5) % through nitrogen, carbon dioxide (CO_2_) sensor control chamber. The modelling was carried out six days per week for four consecutive weeks.

### Preparation of QBPF and medication

QBPF was provided by the First Affiliated Hospital of Anhui University of Traditional Chinese Medicine (Hefei, Anhui, China), including Huangqi (Radix Astragali), Shengshaishen (sun-dried ginseng), Chuanxiong (Ligusticum chuanxiong Hort), Xiebai (Allium macrostemon Bunge), Tinglizi (Lepidium seed), Wuweizi (Schisandra) and Dilong (earthworm). The drug powder in the capsule was carefully ground and prepared solution with 0.9% saline. The dose of QBPF was determined by calculating the clinical equivalent dose using a conversion coefficient from human to rat based on body surface area, as instructed by multiple previous studies [37]. The detailed calculation formula based on the standard body weight of adults and rats is as follows: “Clinical equivalent dose of rat (QBPF, g/ kg) = clinical dose × conversion coefficient = 4.8 g/60 kg × 6.2 ≈ 0.05 g/ kg”.

After successfully preparing animal models, the QBPF group rats were administered intragastric according to the volume of 10 mL/kg rats’ weight with QBPF solution. The model group was given the same volume of saline for two weeks. The control group were raised in a normal environment and gavage of saline (10 ml/kg) for two weeks.

### Pulmonary function measurement

Spirometry data were obtained using the animal pulmonary function analysis system (AniRes 2005, Beijing Beilanbo Technology Co., Ltd). Rats were sedated with 2% pentobarbital (1 ml/Kg), tracheostomized, and intubated. Then rats were placed supine in the body chamber and connected to the system. According to the procedures, the FEV 0.3 (Forced expiratory volume in 0.3 s), FVC (Forced vital capacity) and (FEV 0.3/FVC) % were automatically measured. At least three acceptable manoeuvres for each test of every rat were conducted to obtain reliable mean spirometry data.

### Flow cytometry

The Th17 and Treg cells in the rat anticoagulant peripheral blood were detected using incubation with the corresponding antibodies under dark conditions. For Th17 cells, firstly, side scatter (SSC) was used as the ordinate, forward scatter (FSC) as the abscissa and lymphocyte population were set as the gates. Then use fluorescein isothiocyanate (FITC)-conjugated anti-CD4 (cat.no.201505; clone.no.W3/25; Biolegend) as the abscissa and phycoerythrin (PE)-conjugated anti-interleukin (IL)-17A (cat.no.bs-1183R-PE; Bioss) as the ordinate to mark the CD4^+^IL-17^+^ Th17 cells. For the Treg cells analysis, first, SSC was used as the ordinate, FSC as the abscissa and lymphocyte population were set as the gates. Then use SSC as the ordinate and FITC-conjugated anti-CD4 (cat.no.201505; Biolegend) as the abscissa to circle the CD4^+^T cell gate. Finally, use Alexa Fluor 647-conjugated anti-CD25 (cat.no.bs-0577R-A647; Bioss) as the ordinate and PE-conjugated anti-forkhead box protein p3 (Foxp3) (cat.no.320007; clone.no.150D; Biolegend) as the abscissa to mark the CD25^+^FOXP3^+^ Treg cells. Ultimately, the labelled cells were analyzed on a CytoFLEX flow cytometer (Beckman, USA), using Flow Jo software V10 for statistical analysis.

### Enzyme-linked immunosorbent assay (ELISA)

Peripheral blood of each rat was collected and centrifuged at 4℃ and 3000 rpm/min for 15 min. The supernatant was taken, and the serum levels of IL-17A (cat.no.JYM0480Ra), IL-10 (cat.no.JYM0651Ra), CC chemokine ligand (CCL) 20 (cat.no.JYM0644Ra) and CC chemokine receptor (CCR) 6 (cat.no.JYM1313Ra) were performed using the corresponding ELISA kit (Wuhan ColorfulGene Biological Technology Co., LTD). The experimental procedure was based on the manufacturer's instructions.

### Western blotting

After the lysis of lung tissues with RIPA (cat.no.P0013B; Beyotime), the supernatant was collected, containing the total tissue protein. The protein was separated by sodium dodecyl sulphate–polyacrylamide gel electrophoresis (SDS-PAGE) (cat.no.S8010, cat.no.T8090; Solarbio) and transferred to polyvinylidene fluoride (PVDF) membranes (cat.no.IPVH00010; Millipore) for Western blot assay. After being blocked with 5% skim milk for 2 h, the PVDF membranes were incubated with primary antibodies [rabbit anti-rat retinoid related orphan receptor (ROR) γt (1:1500, cat.no.bs-10647R; bioss), rabbit anti-rat Foxp3 (1:1000, cat.no.bs-23074R; bioss) and mouse anti-rat glyceraldehyde-3-phosphate dehydrogenase (GAPDH) (1:2000, cat.no.TA-08; clone.no.OTI2D9; Zsbio)] overnight at 4 °C. GAPDH was used as a loading control. After being washed three times with PBST, the membranes were incubated with horseradish peroxidase (HRP)-conjugated secondary antibodies (goat anti-rabbit, 1:20,000; cat.no.ZB-2301; Zsbio) at room temperature for 1.2 h, washed three times with PBST. Proteins were detected with ECL luminescence kits (cat. no.340958; Thermo) and analyzed with Image-J software. The results were presented as relative expression levels related to corresponding internal control GAPDH.

### Immunohistochemistry

The expression of RoRγt and Foxp3 in lung tissues was analyzed by immunohistochemistry after fixation with 4% paraformaldehyde and paraffin embedding. All sections were dewaxed in xylene and rehydrated by ethanol gradient elution. After that, the antigen was retrieved in citric antigen retrieval buffer before being blocked with 3% H_2_O_2_. Subsequently, the sections were incubated at 37 °C for 1 h with primary antibodies, including rabbit anti-rat ROR γt (1:200, cat.no.bs-10647R; bioss) and rabbit anti-rat Foxp3 (1:200, cat.no.bs-23074R; bioss). After washing three times with PBS, the slides were incubated with secondary antibodies [mouse/rabbit universal secondary antibody kit (cat.no.PV-6000; Zsbio)] at 37 °C for 30 min before being stained by DAB and counterstained with hematoxylin. The images were captured by light microscopy. Five visual fields at 200 × magnification were randomly selected for each sample, and the mean optical density of RORγt and Foxp3 was measured using Image-Pro Plus 6.0 software.

### Microbiota analyses

Gut contents (GC) of each rat were collected separately with a sterile instrument. Fresh samples of gut contents were rapidly frozen in liquid nitrogen using a cryopreservation tube and stored at − 80 °C for microbial analysis. The right lung lobe was ligated with a cotton thread, and the bronchoalveolar lavage fluid (BALF) was collected from the left lung lobe. The left lung was lavaged three times with 5 mL of physiological saline. The lavage fluid was about 4–5 mL was recovered and then stored in a − 80 °C refrigerator.

Bacterial DNA was isolated from the gut contents and BALF samples using a MagPure Soil DNA LQ Kit (Magan) following the manufacturer’s instructions. The purity and concentration of DNA were determined by agarose gel electrophoresis. PCR amplification of the V3-V4 hypervariable regions of the bacterial 16S rRNA gene used universal primer pairs (343F: 5′-TACGGRAGGCAGCAG-3′; 798R: 5′-AGGGTATCTAATCCT-3′). The Amplicon quality was visualised using gel electrophoresis. The PCR products were purified with Agencourt AMPure XP beads (Beckman Coulter Co., USA) and quantified using a Qubit dsDNA assay kit. The concentrations were then adjusted for sequencing. Sequencing was performed on an Illumina NovaSeq 6000 with two paired-end read cycles of 250 bases each (Illumina Inc., San Diego, CA; OE Biotech Company; Shanghai, China). The sequencing data obtained were further processed and analysed.

### Statistical analysis

GraphPad Prism (GraphPad Software version 8.0, San Diego, CA, USA) was used for statistical analysis. All parametric data were analyzed using one-way analysis of variance (ANOVA), followed by Brown-Forsythe and Welch ANOVA posthoc multiple comparison tests. All nonparametric data were analyzed using the Kruskal-wills test. Statistical significance was established at *p* < 0.05. The data were represented as mean ± standard error. Spearman correlation analysis was performed using R version 3.1.1 to evaluate the correlations between bacterial taxa and lung function and immune features.

## Results

### Pulmonary function

From a general point of view, compared with the control group, the weight of rats in the model group increased slowly, the spirit was inactive, the hair was untidy, the colour was dull, and the back was arched. Compared with the model group, the weight of rats in the QBPF group increased to some extent, and their mental state and activity improved to varying degrees.

**Fig. 1:**
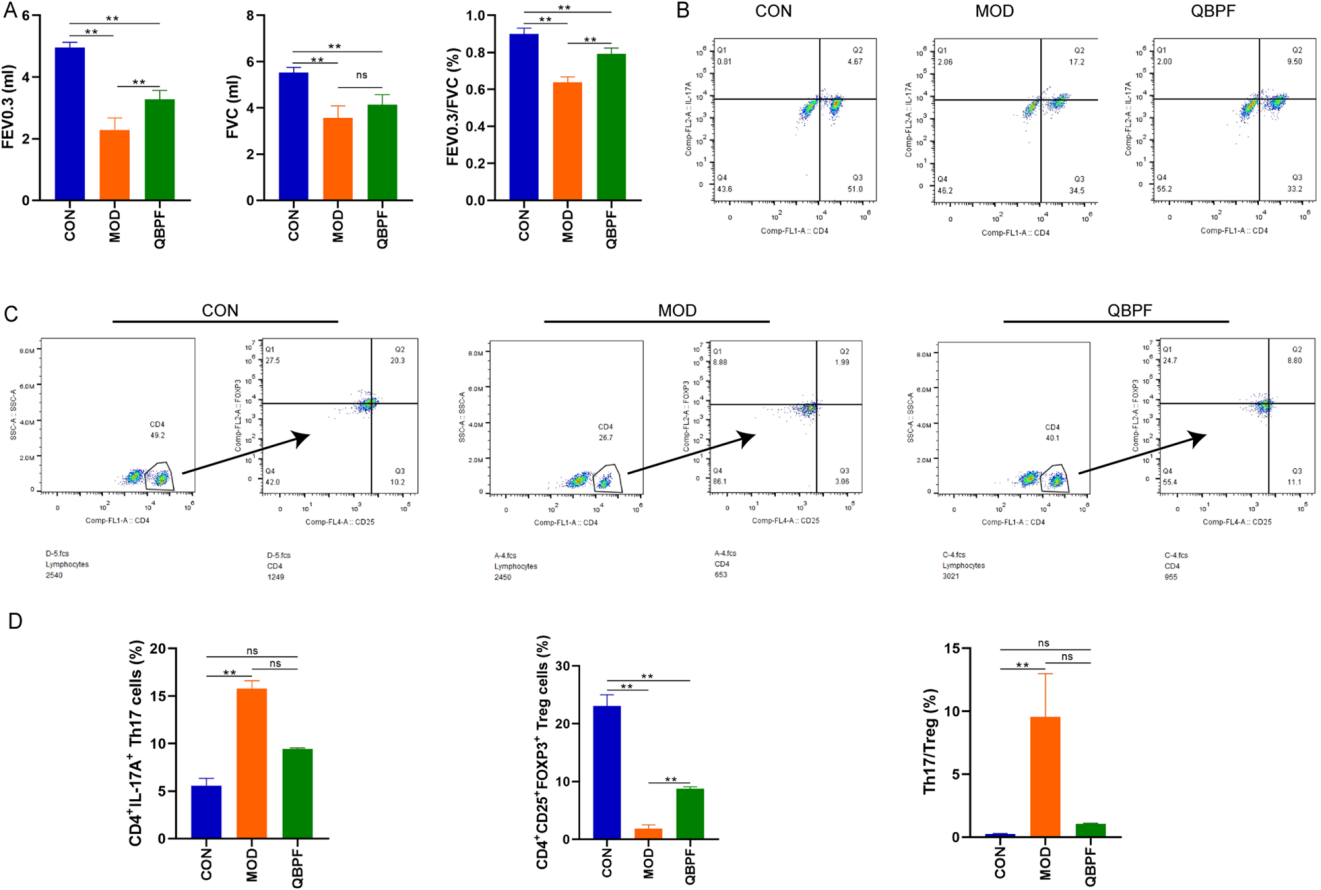
Pulmonary function and flow cytometry of Th17 and Treg cells in the peripheral blood. Pulmonary function showing FEV 0.3, FVC and (FEV 0.3/FVC) % of all groups (**A**). The representative plots of Th17 cells were gated by CD4^+^IL17^+^ (**B**). Th17% refers to the ratio of Th17 to total lymphocytes. The representative plots of Treg cells were gated by CD4^+^CD25^+^Foxp3^+^ (**C**). Treg% refers to the ratio of Treg to CD4^+^ T cells. The Th17/Treg ratio in peripheral blood (**D**). Data are expressed as mean ± standard deviation (SD). *P < 0.05, **P < 0.01

FEV 0.3, FVC and (FEV 0.3/FVC) % of all groups were detected to reflect pulmonary function. As shown in Fig. 1A, compared with the control group, these parameters were dramatically lower in the COPD and QBPF groups (*P* < 0.01). Following QBPF treatment, FEV 0.3, FVC and (FEV 0.3/FVC) % in the QBPF group were significantly improved compared to rats in the COPD group.

Based on these measurements and analyses, we have successfully established a COPD rat model and confirmed that QBPF treatment could alleviate the overall symptoms of COPD.

### Analysis of the Th17/Treg cells balance

Flow cytometry was performed to measure the percentage of Th17 and Treg cells from peripheral blood. Compared with the model group, the proportion of Tregs in the control and treatment group was significantly increased (Fig. 1C, D). Compared with the model group, the proportion of Th17 cells in the control and treatment group was significantly reduced (Fig. 1B, D); meanwhile, the ratio of Th17 to Treg declined significantly (Fig. 1D). Thus it can be seen that QBPF can increase Treg while reducing Th17 cells expression, and it can treat COPD by decreasing the ratio of Th17 to Treg.

**Fig. 2:**
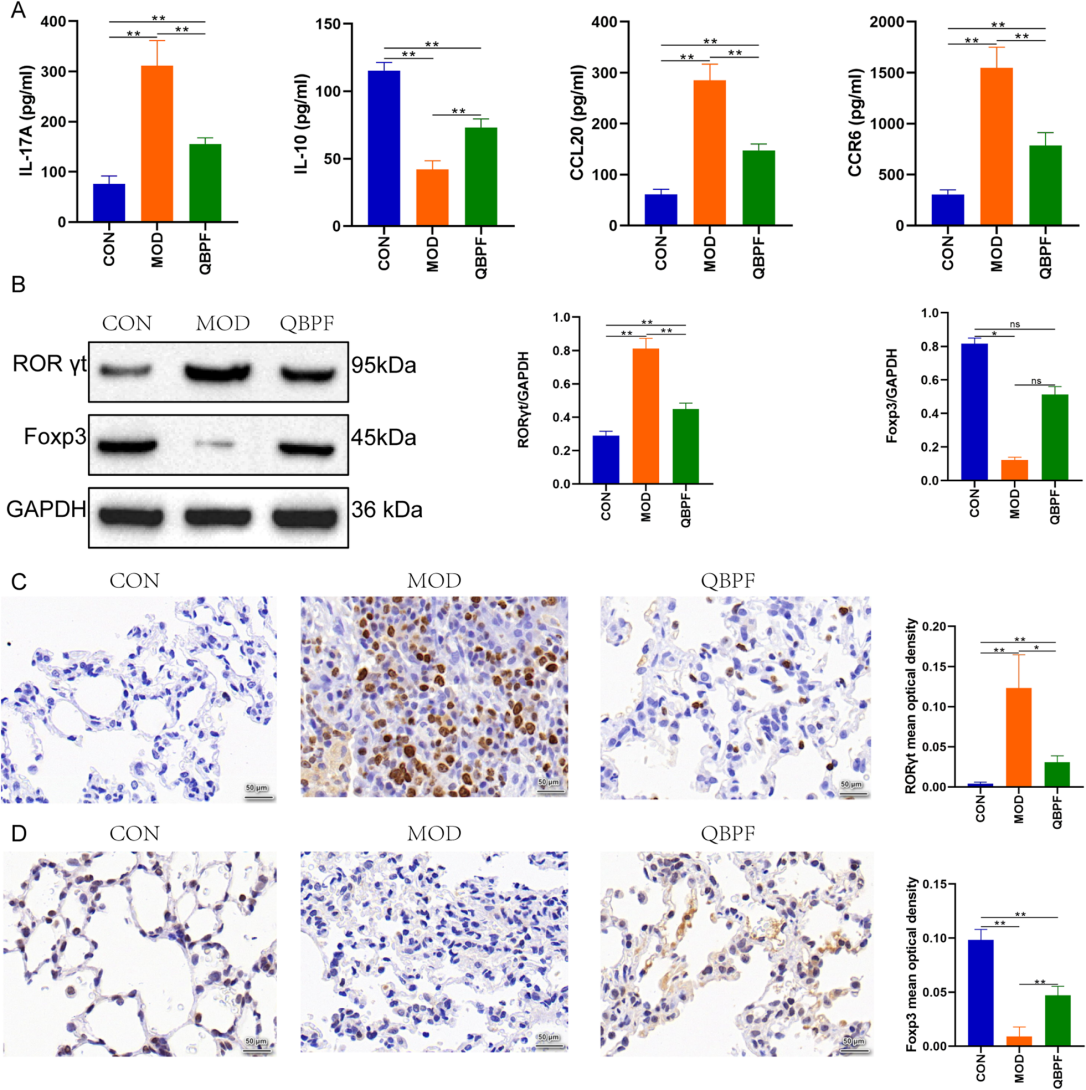
Cytokines and transcription factors related to Th17 cells and Treg cells. ELISA showing IL-17A, IL-10, CCL20, and CCR6 protein expression levels (**A**). Western blot analysis and quantification of western blotting showing RORγt and Foxp3 (**B**). The immunohistochemistry staining and the mean optical density of RORγt (**C**) and Foxp3 (**D**) in the lung tissue. Data are expressed as mean ± standard deviation (SD). *P < 0.05, **P < 0.01

ELISA was used to determine the expression levels of related cytokines of Th17 cells and Tregs in rat serum. A significant increase in IL-17A was observed in the model group compared with the control group (Fig. 2A). Moreover, the expression of IL-10 decreased significantly in the model group (Fig. 2A). Treatment with QBPF significantly reduced levels of IL-17A compared with the model group (*p* < 0.01) and increased levels of IL-10 (*p* < 0.01). The content of CCL20 and CCR6 in serum was significantly increased in the COPD model group compared with controls and significantly decreased in the QBPF treated group compared with the model group (Fig. 2A). Thus, these findings suggested that the QBPF protective effect of COPD in the rat may partly be due to the inhibition of inflammatory factors and regulation of immune-related cytokines.

To investigate the expression levels of RORγt and Foxp3, transcription factors of Th17 cells and Tregs, in the lung tissue of each group of rats, the western blot and immunohistochemistry were performed. Histological analyses showed that for the control group rats, the alveolar wall and the alveolar structure were intact and normal; no significant inflammatory cell infiltration was seen (Fig. 2C, 2D). While in the model group, the alveolar structure of the rats was disturbed, as evidenced by thinning or fracturing of the alveolar wall, reduced alveolar elasticity, cystic dilatation, enlarged alveolar cavities, and partial fusion into pulmonary blisters. There were also many inflammatory cells infiltration of lung tissue in the model group (Fig. 2C, D). Notably, the model group rats' COPD-related lung histopathological damage was markedly relieved by QBPF treatment (Fig. 2C, 2D). Compared with the control group, the expression levels of RORγt in the lung tissue of the model group increased significantly. However, compared with the model group, the expression levels of RORγt of the QBPF treatment group were significantly reduced (Fig. 2B, C). Compared with the rat in the control group, the positive expression of Foxp3 in the model group was significantly reduced. However, treatment with QBPF increased the positive expression of Foxp3 compared with that in the model group (Fig. 2B, D).

### Analysis of the alpha and beta diversity of intestinal and pulmonary microbiota

To detect the changes in intestinal and pulmonary microbiota in COPD group rats and whether the QBPF intervention can regulate the changes in microbiota, we performed 16S rRNA gene sequencing of gut contents and BALF samples from rats.

**Fig. 3:**
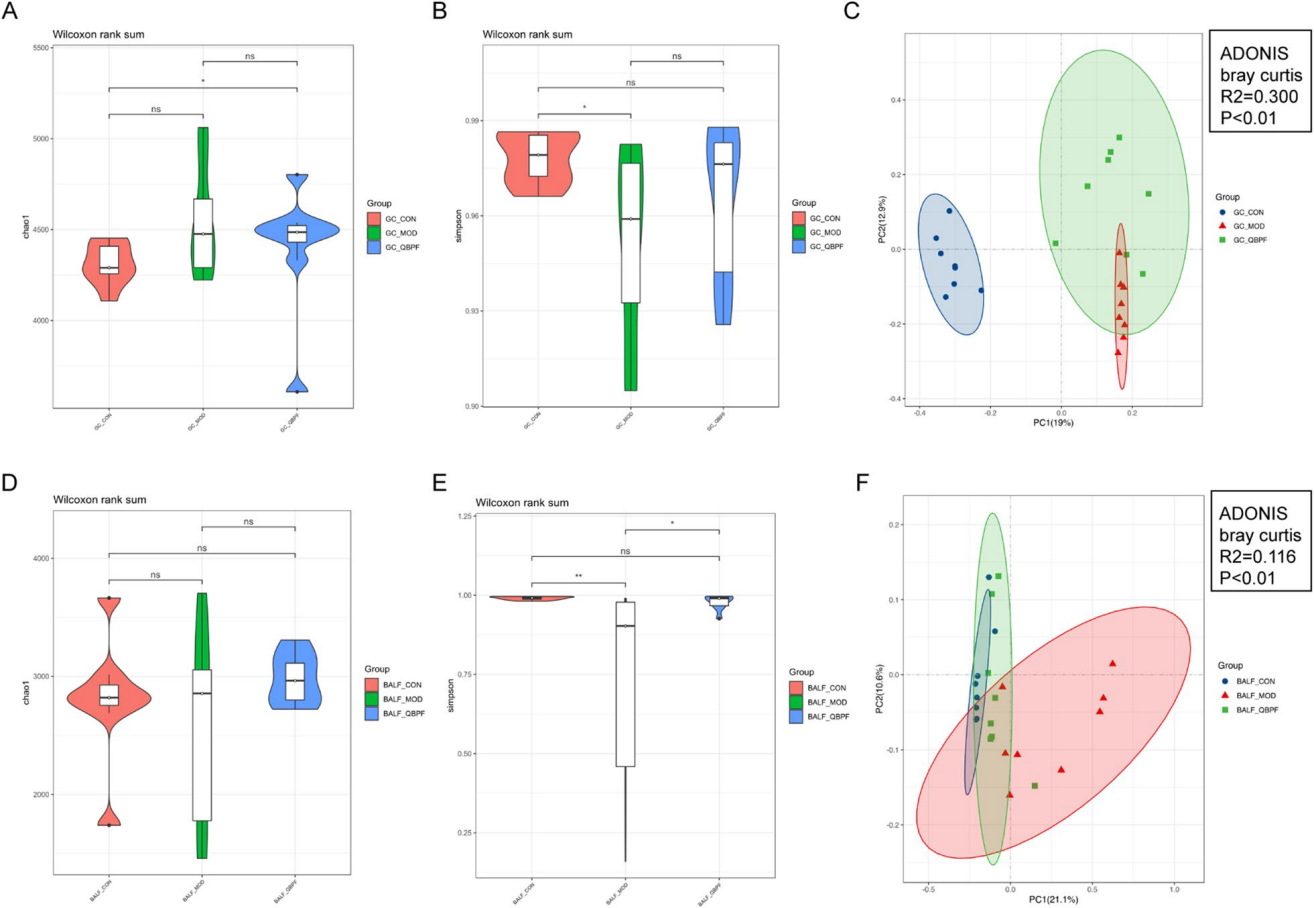
Analysis of the microbial diversity of the gut contents (**A**–**C**) and BALF (**D**–**F**). Comparison of alpha diversity (as assessed by the Chao 1 (**A**, **D**) and Simpson (**B**, **E**) indices) and beta diversity (as assessed by the PCoA (**C**, **F**)). *P < 0.05, **P < 0.01

We first compared the diversity of the gut contents microbiomes in different groups. The species richness was similar between the control and model groups (*P* > 0.05), as well as between the model and QBPF group (*P* > 0.05, Fig. 3A). The species evenness was strikingly decreased in the model group than in the control group (*P* < 0.05) and was slightly increased but not significantly different following QBPF treatment (*P* > 0.05, Fig. 3B). The principal coordinates analysis (PCoA) plot indicated a distinctly different gut contents microbiota among the three groups (*P* < 0.01, Fig. 3C).

For the diversity analysis of the BALF microbiomes, species richness according to the Chao1 index was similar in the control, model, and QBPF groups (*p* > 0.05, Fig. 3D). The species evenness according to the Simpson index was lower in the model group than in the control group (*P* < 0.01) and QBPF group (*P* < 0.05), indicating that the BALF microbiota in COPD model rats was characterized by a lower evenness than that in the control group. At the same time, QBPF treatment could increase species evenness (Fig. 3E). PCoA plot revealed a distinctly different BALF microbiota among the three groups (*P* < 0.01, Fig. 3F). Similar to the case of gut contents microbiota, the QBPF group was closer to the control group, indicating that the QBPF treatment could influence the integral structure of the intestinal and pulmonary microbiota of the model group rats towards normal.

### Analysis of the taxonomic composition of intestinal and pulmonary microbiota

To investigate the difference in intestinal and pulmonary microbiota community structure in the three groups, we analyzed each group's top fifteen dominant taxa at the phylum and genus levels.

**Fig. 4:**
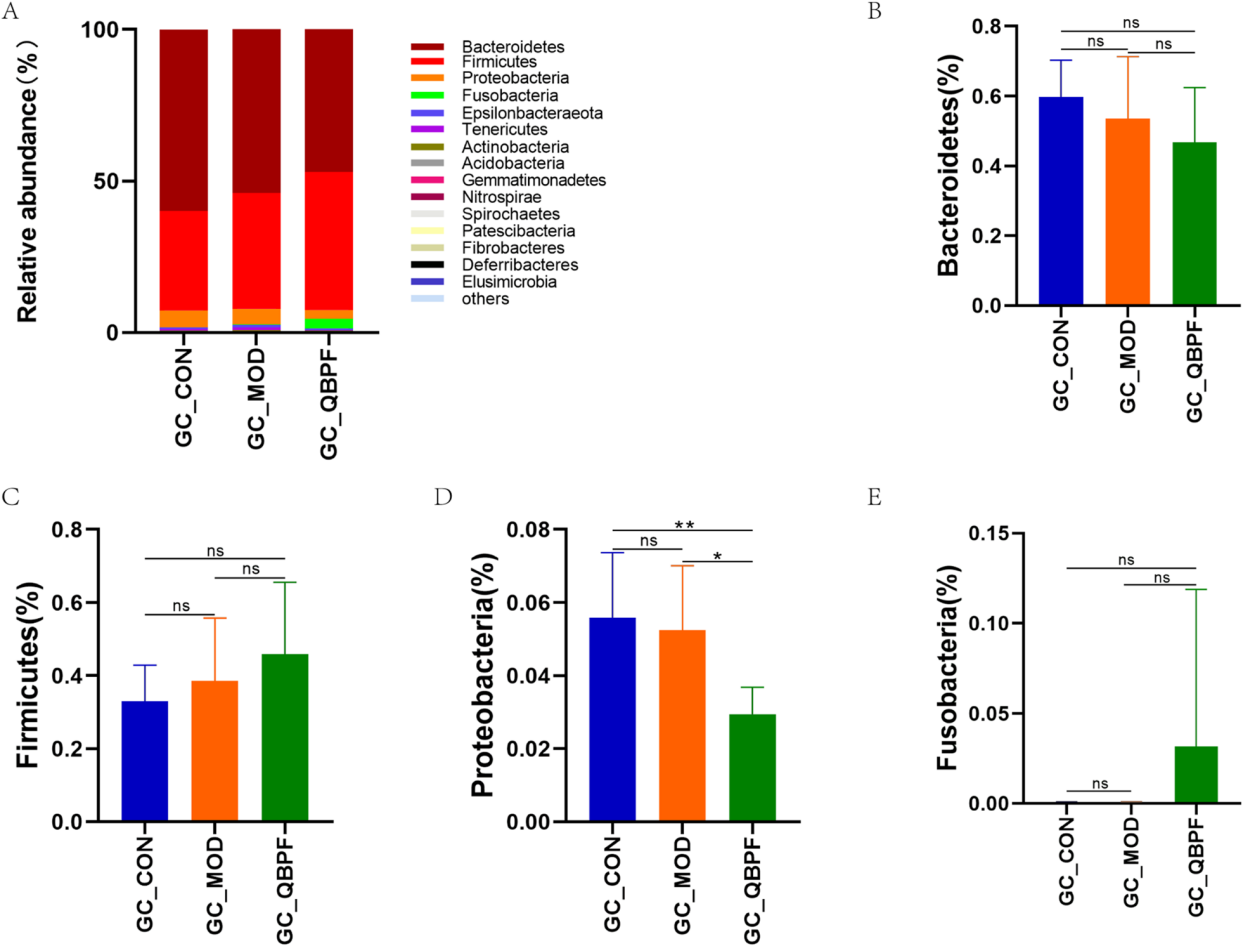
Relative abundance of the most prevalent intestinal bacteria at the phylum level. Relative proportions of major bacterial phyla (**A**) and those significantly differentially expressed among the three groups (**B**–**E**). Data are expressed as mean ± standard deviation (SD). *P < 0.05, **P < 0.01

**Fig. 5:**
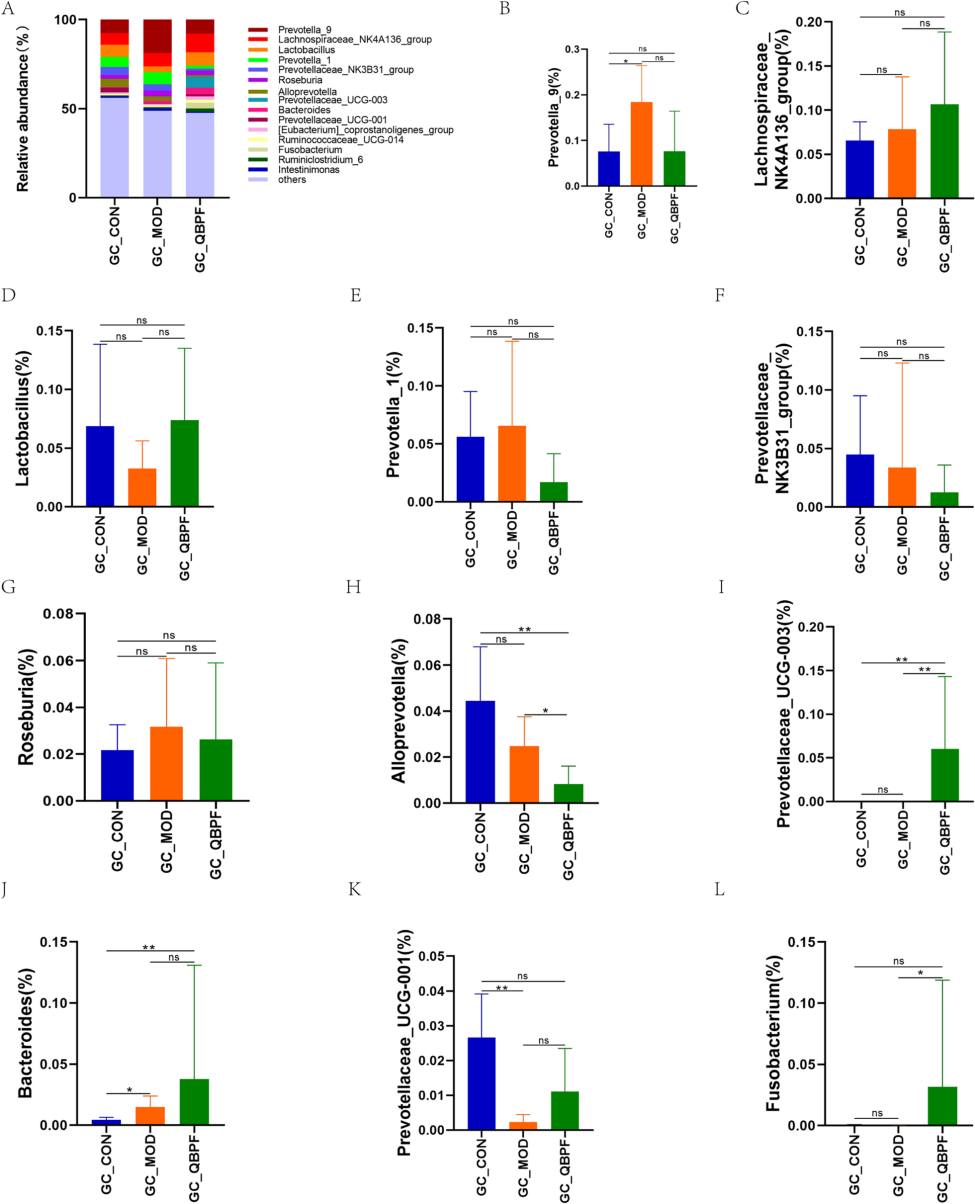
Relative abundance of the most prevalent intestinal bacteria at the genus level. Relative proportions of major bacterial genera (**A**) and those significantly differentially expressed among the three groups (**B**–**L**). Data are expressed as mean ± standard deviation (SD). *P < 0.05, **P < 0.01

The intestinal microbial community structure analysis showed that the dominant phyla in the three groups, accounting for more than 97%, include *Bacteroidetes*, *Firmicutes*, *Proteobacteria*, and *Fusobacteria* (Fig. 4A). Compared to the control group, the proportion of *Bacteroidetes* and *Proteobacteria* declined in the model group, while the ratio of *Firmicutes* climbed in the model group. After QBPF intervention, the changing trends of *Bacteroidetes*, *Firmicutes*, and *Proteobacteria* were further deepened (Fig. 4B–D). The relative abundance of *Fusobacteria* was decreased in the model group than in the control group and increased after QBPF intervention (Fig. 4E). At the genus level, the dominant genera with a relative abundance greater than 2% include *Prevotella_9*, *Lachnospiraceae_NK4A136_group*, *Lactobacillus*, *Prevotella_1*, *Prevotellaceae_NK3B31_group*, *Roseburia*, *Alloprevotella*, *Prevotellaceae_UCG-003*, *Bacteroides*, *Prevotellaceae_UCG-001*, and *Fusobacterium* (Fig. 5A). The *Lactobacillus*, *Prevotellaceae_UCG-001*, and *Fusobacterium* levels showed downregulation in the model group compared to that in the control group and upregulation in the QBPF group compared to the model group (Fig. 5D,K, L). The *Prevotella_9*, *Prevotella_1*, and *Roseburia* levels showed upregulation in the model group compared to the control group and downregulation in the QBPF group compared to the model group (Fig. 5B, E, G). Compared to the control group, the proportion of *Lachnospiraceae_NK4A136_group, Prevotellaceae_UCG-003,* and *Bacteroides* increased in the model group (Fig. 5C, I, J), while the proportion of *Prevotellaceae_NK3B31_group* and *Alloprevotella* decreased in the model group (Fig. 5F, H), these microbiota trends continued after the QBPF intervention.

**Fig. 6:**
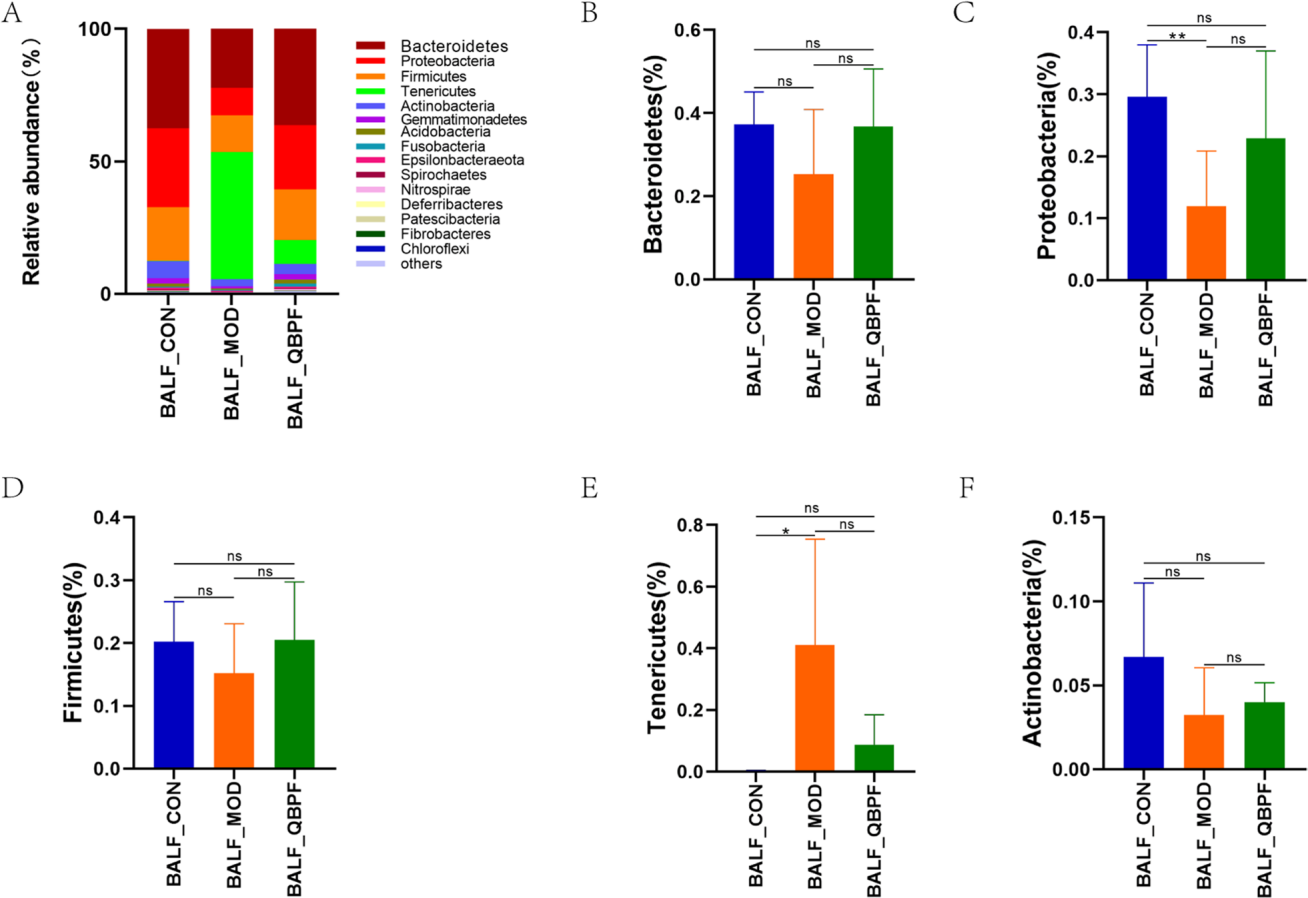
Relative abundance of the most prevalent pulmonary bacteria at the phylum level. Relative proportions of major bacterial phyla (**A**) and those significantly differentially expressed among the three groups (**B**–**F**). Data are expressed as mean ± standard deviation (SD). *P < 0.05, **P < 0.01

**Fig. 7:**
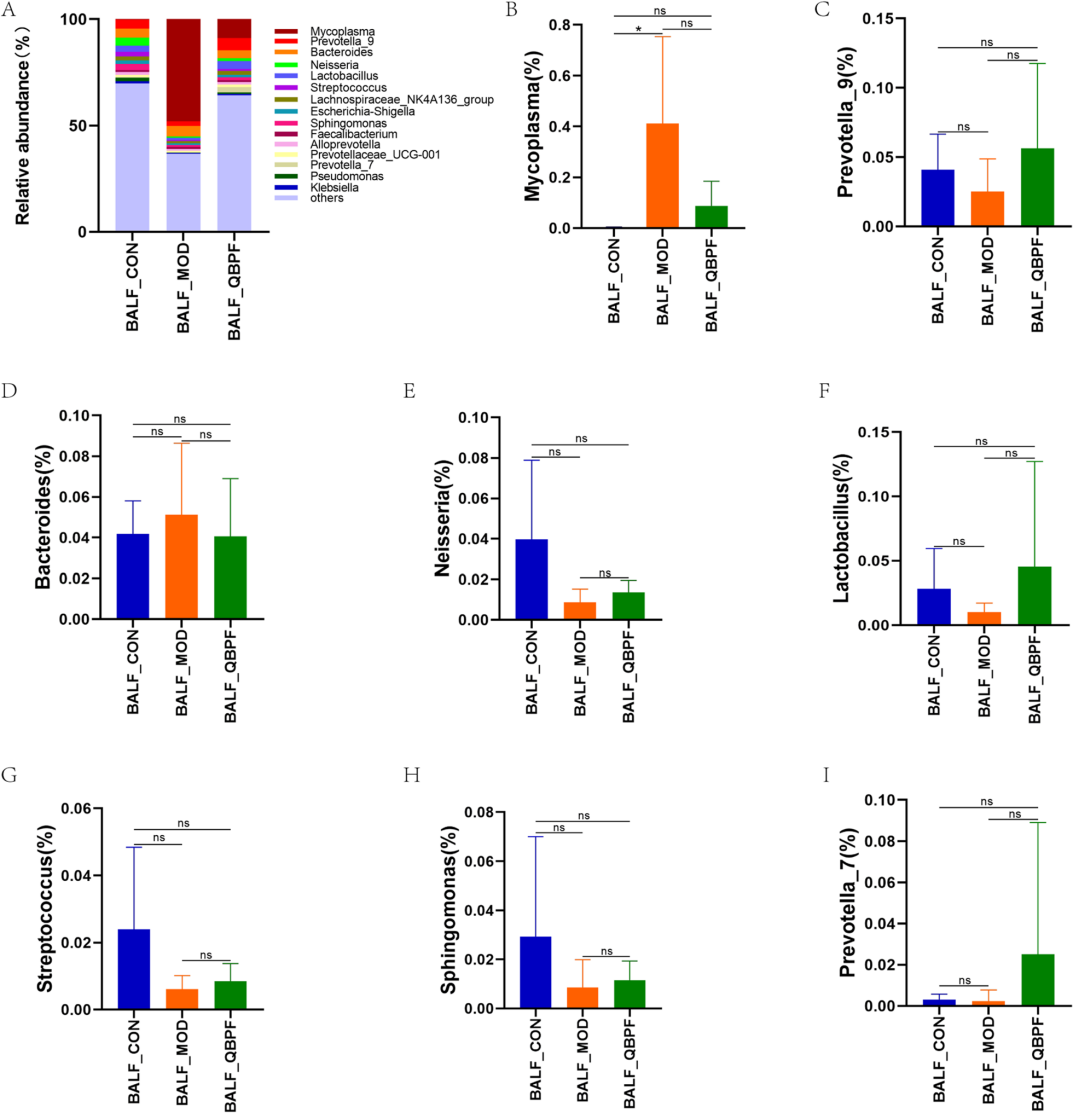
Relative abundance of the most prevalent pulmonary bacteria at the genus level. Relative proportions of major bacterial genera (**A**) and those significantly differentially expressed among the three groups (**B**–**I**). Data are expressed as mean ± standard deviation (SD). *P < 0.05, **P < 0.01

Regarding pulmonary microbiota, the top five dominant phyla were *Bacteroidetes*, *Proteobacteria*, *Firmicutes*, *Tenericutes*, and *Actinobacteria*, which accounted for over 92% of the total sequences in all three groups (Fig. 6A). Compared to the control group, the proportion of *Bacteroidetes*, *Proteobacteria*, *Firmicutes*, and *Actinobacteria* declined in the model group and climbed in the QBPF group (Fig. 6B–D, F), and the *Tenericutes* level rose in the model group and dropped in the QBPF group (Fig. 6E). The dominant genera with a relative abundance of more than 2% in BALF include *Mycoplasma*, *Prevotella_9*, *Bacteroide*, *Neisseria*, *Lactobacillus*, *Streptococcus*, *Sphingomonas*, and *Prevotella_7* (Fig. 7A). Compared to the control group, the proportion of *Prevotella_9*, *Neisseria*, *Lactobacillus*, *Streptococcus*, *Sphingomonas*, and *Prevotella_7* dropped in the COPD model group, but it went up in the QBPF group (Fig. 7C, E–I). On the contrary, the *Mycoplasma* and *Bacteroide* level increased in the model group compared to the control group and dropped in the QBPF group (Fig. 7B, D).

### Analysis of the significant differences of intestinal and pulmonary microbiota

Line Discriminant Analysis (LDA) Effect Size (LEfSe) can detect species that differ significantly in abundance between groups. We selected an LDA score higher than 3 to represent the most enriched species in each group.

**Fig. 8:**
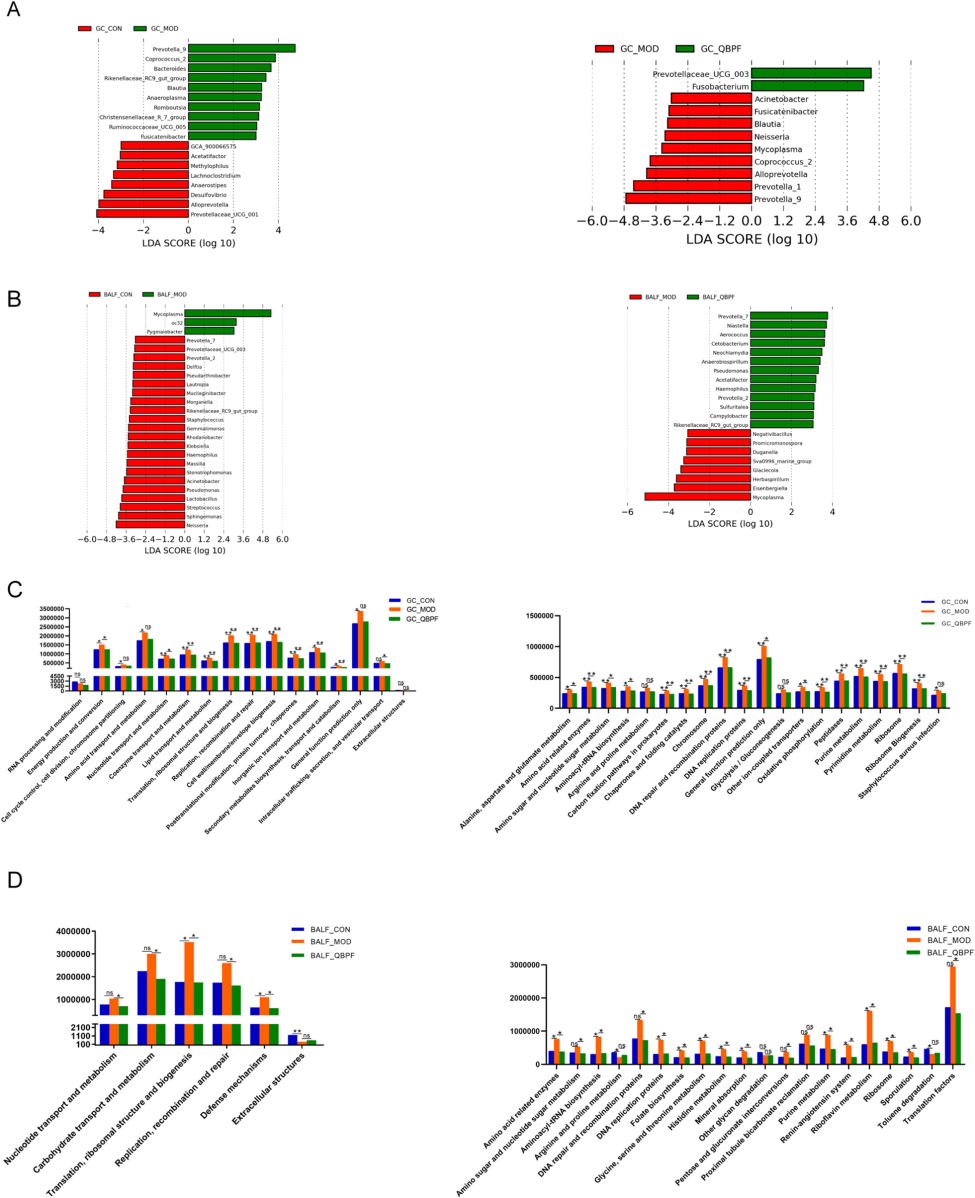
Differential genera identification and the differences in metabolic pathways in each group. LEfSe revealed differentially abundant genera at the gut (**A**) and lung (**B**) in all groups. The differences in metabolic pathways by PICRUSt functional analysis (**C**, **D**). Comparison of functional analysis of intestinal microbiota in three groups at COG and KEGG level 3 (**C**). Comparison of functional analysis of pulmonary microbiota in three groups at COG and KEGG level 3 (**D**). *P < 0.05, **P < 0.01

In terms of gut microbiota, compared with the control group, *Bacteroides*, *Christensenellaceae_R_7_group*, *Ruminococcaceae_UCG_005*, *Anaeroplasma*, *Fusicatenibacter*, *Coprococcus_2*, *Prevotella_9*, *Blautia*, *Rikenellaceae_RC9_gut_group*, and *Romboutsia* were more abundant in the COPD group. However, *Anaerostipes*, *Methylophilus*, *Acetatifactor*, *Lachnoclostridium*, *Alloprevotella*, *GCA_900066575*, *Desulfovibrio*, and *Prevotellaceae_UCG_001* exhibited lower relative proportions in the COPD rats compared to the control group (Fig. 8A). Compared with the model group, *Fusobacterium* and *Prevotellaceae_UCG_003* were enriched, whereas *Fusicatenibacter*, *Neisseria*, *Acinetobacter*, *Coprococcus_2*, *Mycoplasma*, *Prevotella_9*, *Prevotella_1*, *Alloprevotella*, and *Blautia* presented lower abundances in the QBPF group (Fig. 8A).

LEfSe analysis of different lung species for three groups was also performed. Compared with the control group, *Klebsiella*, *Sphingomonas*, *Streptococcus*, *Lactobacillus*, *Mucilaginibacter*, *Stenotrophomonas*, *Delftia*, *Neisseria*, *Acinetobacter*, *Staphylococcus*, *Pseudomonas*, *Prevotella_2*, *Prevotella_7*, *Lautropia*, *Rhodanobacter*, *Pseudarthrobacter*, *Rikenellaceae_RC9_gut_group*, *Haemophilus*, *Morganella*, *Gemmatimonas*, *Prevotellaceae_UCG_003*, and *Massilia* were less abundant in the COPD group. On the contrary, *Pygmaiobacter*, *Mycoplasma*, and *oc32* exhibited higher relative proportions in the COPD group compared to the control group (Fig. 8B). *Cetobacterium*, *Campylobacter*, *Aerococcus*, *Acetatifactor*, *Pseudomonas*, *Neochlamydia*, *Prevotella_2*, *Prevotella_7*, *Sulfuritalea*, *Niastella*, *Rikenellaceae_RC9_gut_group*, *Haemophilus*, and *Anaerobiospirillum* were enriched, but *Promicromonospora*, *Duganella*, *Herbaspirillum*, *Glaciecola*, *Eisenbergiella*, *Sva0996_marine_group*, *Negativibacillus*, and *Mycoplasma* presented lower abundances in the QBPF group than the COPD group (Fig. 8B).

### Analysis of the functional prediction of intestinal and pulmonary microbiota

Phylogenetic Investigation of Communities by Reconstruction of Unobserved States (PICRUSt) was used to perform clusters of orthologous groups (COG) and Kyoto Encyclopedia of Genes and Genomes (KEGG) functional prediction for sequenced genomes to determine potential functional changes in the gut and lung microbial composition of different groups of rats.

Functional prediction of intestinal microbiota based on the COG database showed that the energy production and conversion, nucleotide transport and metabolism, coenzyme transport and metabolism, lipid transport and metabolism, translation, ribosomal structure and biogenesis, replication, recombination and repair, cell wall/membrane/envelope biogenesis, posttranslational modification, protein turnover, chaperones, inorganic ion transport and metabolism, and secondary metabolites biosynthesis, transport and catabolism pathways altered dramatically in the COPD group (Fig. 8C). Functional prediction of pulmonary microbiota based on the COG database found that the translation, ribosomal structure and biogenesis, and defence mechanisms pathways altered markedly in the COPD group (Fig. 8D).

Functional analysis of intestinal microbiota based on the KEGG database showed that alanine, aspartate and glutamate metabolism, amino acid-related enzymes, amino sugar and nucleotide sugar metabolism, aminoacyl-tRNA biosynthesis, carbon fixation pathways in prokaryotes, chaperones and folding catalysts, chromosome, DNA repair and recombination proteins, DNA replication proteins, general function prediction only, other ion-coupled transporters, oxidative phosphorylation, peptidases, purine metabolism, pyrimidine metabolism, ribosome, and ribosome biogenesis were more abundant in the COPD group than the control group. Still, they significantly decreased after the QBPF administration (Fig. 8C).

Functional analysis of pulmonary microbiota based on the KEGG database indicated that amino acid-related enzymes, aminoacyl-tRNA biosynthesis, DNA replication proteins, folate biosynthesis, glycine, serine and threonine metabolism, histidine metabolism, mineral absorption, purine metabolism, renin-angiotensin system, riboflavin metabolism, ribosome, and sporulation pathways were more abundant in the COPD group than controls. In contrast, they were significantly decreased upon the QBPF administration (Fig. 8D).

### Correlation analysis of the intestinal and pulmonary microbiota

**Fig. 9:**
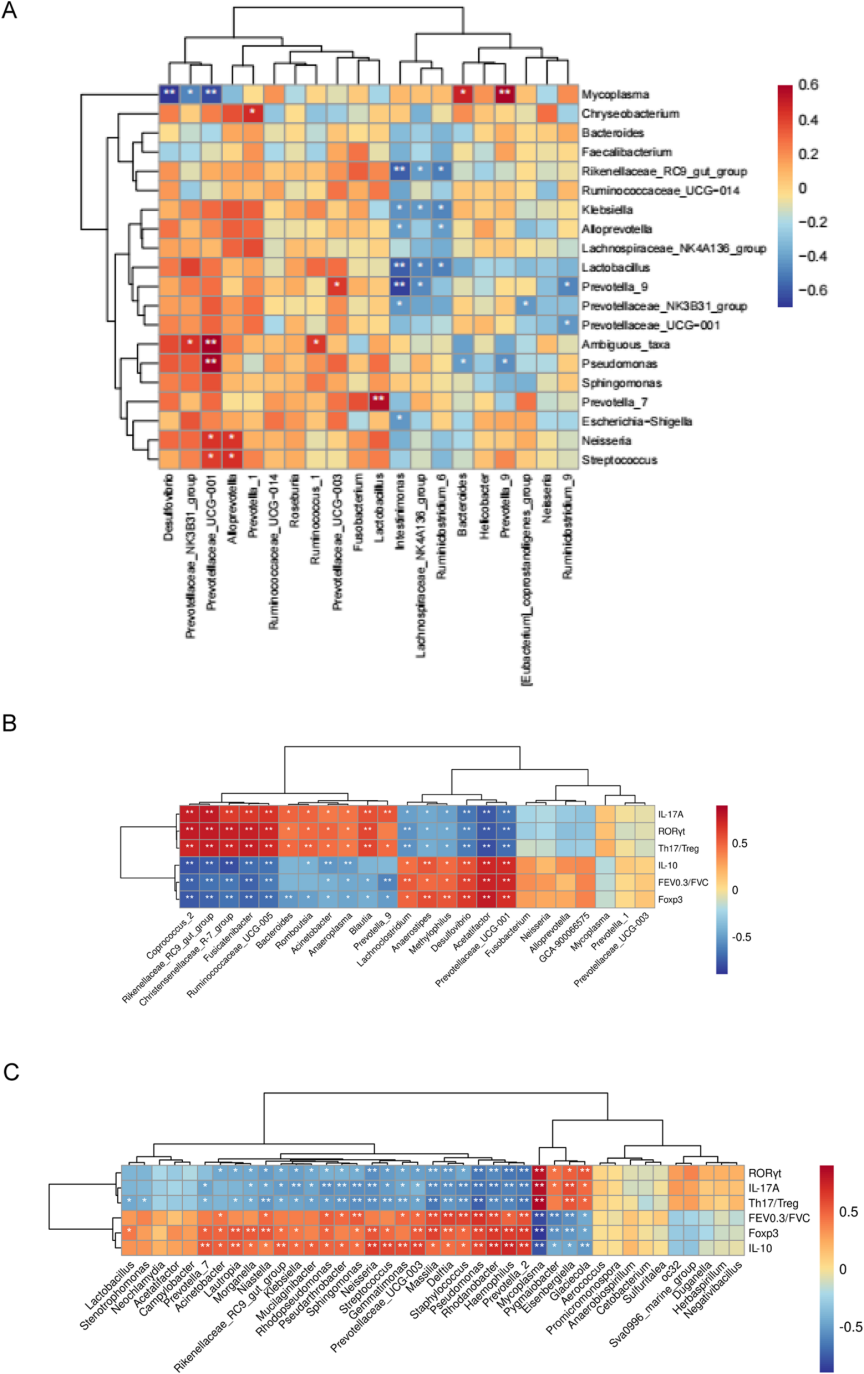
Heatmap of correlation of dominant genera and the correlation between key genera and certain parameters. Heatmap of correlation analysis of intestinal and pulmonary dominant genera (**A**). The abscissa axis is the dominant genera of intestinal microbiota, and the ordinate axis is the dominant genera of pulmonary microbiota. In the figure, red represents positive correlation, blue represents negative correlation, and the darker the colour, the greater the correlation coefficient. The heatmap analysis of Spearman correlation between key genera in the gut (**B**) and lung (**C**) and lung function and immune parameters. The red indicates a positive correlation, and the blue indicates a negative correlation.  *p < 0.05, ** *p *< 0.01

To investigate the relationship between intestinal and pulmonary microbiota, we performed a correlation analysis on the top 20 dominant genera in the relative abundance of intestinal and pulmonary microbiota at the genus level in the experiment (Fig. 9A). There were nine genera simultaneously expressing in the gut and lung, including *Bacteroides*, *Prevotella_9*, *Lactobacillus*, *Neisseria*, *Lachnospiraceae_NK4A136_group*, *Alloprevotella*, *Prevotellaceae_UCG-001*, *Prevotellaceae_NK3B31_group*, and *Ruminococcaceae_UCG-014*, among which *Bacteroides*, *Lactobacillus*, and *Lachnospiraceae_NK4A136_group* were negatively correlated (*P* > 0.05), *Prevotella_9*, *Neisseria*, *Alloprevotella*, *Prevotellaceae_UCG-001*, *Prevotellaceae_NK3B31_group*, and *Ruminococcaceae_UCG-014* were positively correlated (*P* > 0.05). *Mycoplasma* of pulmonary microbiota is positively correlated with *Prevotella_9* (*P* < 0.01) and *Bacteroides* (*P* < 0.05) and negatively correlated with *Prevotellaceae_NK3B31_group* (*P* < 0.05), *Prevotellaceae_UCG-001* (*P* < 0.01), and *Desulfovibrio* (*P* < 0.01) of intestinal microbiota. *Prevotellaceae_UCG-001* of intestinal microbiota is positively correlated with *Ambiguous taxa* (*P* < 0.01) and *Pseudomonas* (*P* < 0.01) of pulmonary microbiota. *Intestinimonas* of the intestinal microbiota are negatively correlated with *Prevotella_9* (*P* < 0.01), *Lactobacillus* (*P* < 0.01), and *Rikenellaceae_RC9_gut_group* (*P* < 0.01) of pulmonary microbiota.

### Correlation analysis of the bacterial genera and lung function and immune features

As shown in Fig. 9B, significant correlations were identified between gut microbiota and lung function and immune parameters. *Lachnoclostridium*, *Anaerostipes*, *Methylophilus*, *Desulfovibrio*, *Acetatifactor*, and *Prevotellaceae_UCG_001*, worsened in the COPD group, were positively associated with lung function and Treg-related cytokines but negatively correlated to Th17/Treg ratio and Th17-related cytokines. On the contrary, *Coprococcus_2*, *Rikenellaceae_RC9_gut_group*, *Christensenellaceae_R_7_group*, *Fusicatenibacter*, *Ruminococcaceae_UCG_005*, *Acinetobacter*, *Anaeroplasma*, *Blautia*, and *Prevotella_9*, enriched in the COPD group, were negatively correlated to lung function and Treg-related cytokines but positively associated with Th17/Treg ratio and Th17-related cytokines. These data suggested the associations between the immunogenic status and lung function and microbiota in the gut in COPD rats.

As shown in Fig. 9C, significant correlations were identified between lung microbiota and lung function and immune parameters. *Mycoplasma*, *Pygmaiobacter*, *Eisenbergiella*, and *Glaciecola*, enriched in the COPD group, presented positive correlations to Th17-related cytokines but negative association coefficients with lung function and Treg-related cytokines. In particular, *Acinetobacter*, *Niastella*, *Pseudarthrobacter*, *Sphingomonas*, *Gemmatimonas*, *Prevotellaceae_UCG_003*, *Massilia*, *Delftia*, *Staphylococcus*, *Pseudomonas*, *Rhodanobacter*, *Haemophilus*, and *Prevotella_2* were positively associated with lung function. Several genera having lower abundances in the COPD group, including *Prevotella_7*, *Lautropia*, *Morganella*, *Niastella*, *Rikenellaceae_RC9_gut_group*, *Klebsiella*, *Mucilaginibacter*, *Pseudarthrobacter*, *Sphingomonas*, *Neisseria*, *Streptococcus*, *Gemmatimonas*, *Prevotellaceae_UCG_003*, *Massilia*, *Delftia*, *Staphylococcus*, *Pseudomonas*, *Rhodanobacter*, *Haemophilus*, and *Prevotella_2*, were negatively correlated to Th17-related cytokines and Th17/Treg ratio, but positively associated with Treg-related cytokines. These data suggested the associations between the immunogenic status and lung function and microbiota in the lung in COPD rats.

## Discussion

### There were differences in intestinal and pulmonary microbiota expression in COPD model rats

The changes in richness, diversity, and community structure of the bacterial microbiome in the gut and lung with COPD model rats were observed using 16S rRNA high-throughput sequencing in this study. There were significant differences in the overall structure of intestinal and pulmonary microbiota between the control and model groups, and QBPF intervention could alleviate the trend of sample differences. It was suggested that intestinal and pulmonary microbiota changes occurred in COPD rats, and QBPF could optimize these changes. Based on the taxonomic composition analysis, the dominant microbiota phyla in the gut and lung include *Bacteroidetes*, *Proteobacteria*, and *Firmicutes* in all groups. *Bacteroidetes* of intestinal and pulmonary microbiota were lower in the model group than in the control group in this experiment, consistent with the previous facts in patients with COPD and mice exposed to diesel exhaust particles [11, 38]. Consistent with previous clinical studies, the proportion of *Firmicutes* in the lung was lower [39], which was higher in the gut [11] in the model group than in the control group. Nevertheless, interestingly, different from COPD and AECOPD patients, the proportion of *Proteobacteria* in the gut and lung was lower in the model group than that of the control group in our study [11, 40]. Many common human pathogens belong to the *Proteobacteria*, and their proportions were negatively correlated with the FEV1/FVC value in stable COPD patients [39]. However, *Proteobacteria* deletion can lead to inflammation under the condition of dysbacteriosis [41].

### There is an imbalance of Th17, Treg cells and their related cytokines in the immune system in COPD model rats

The dynamic equilibrium of Th17 and Treg cells played an essential role in balancing COPD patients’ immune status [4, 42]. We noticed that CCL20, CCR6 and Th17 cells and related cytokines were increased. In contrast, the expression of Treg and associated cytokines were reduced in the COPD model group compared with the control group. Th17 cells have the effect of inducing inflammatory response and increasing the expression of related cytokines IL-17A and transcription factor RORγt. While Treg cells antagonize the inflammatory response, their related cytokine IL-10 and transcription factor Foxp3 expression are decreased in the COPD group. CCL20 and its unique receptor CCR6 are mainly expressed on the surface of Thl7 and Treg cells, which can mediate and regulate the chemotaxis of Thl7 and Treg cells to inflammatory sites in humans [43, 44]. Therefore, CCL20 and CCR6 may be involved in chronic inflammation and immune response of airways and thus may participate in the COPD pathogenesis process [45].

The interactions between microbiota and immunity in the gut and lung are two-way [19, 46–48].

On the one hand, intestinal microbiota affects the local immune system [46]. Furthermore, intestinal microbiota and producing short-chain fatty acids (SCFAs) play an essential role in establishing and regulating the pulmonary immune system in mice and humans [41, 49]. A crucial part of pulmonary microbiota in the maturation and homeostasis of lung immunity has also emerged in mice and humans [50, 51]. On the other hand, Treg cell depletion from the intestinal lamina propria in mice influenced the intestinal microbiota composition [48]. And reduced SCFA production is commonly associated with chronic autoimmune diseases in humans[52]. A significant inflammation in the lung can also sickly transform the pulmonary microbiota composition in COPD patients [53].

### There is a significant relationship between gut-lung axis microecology and pulmonary function and immune function in COPD model rats

Traditional Chinese medicine theory suggests that “the lung stands in interior-exterior relationship with the large intestine” [54], which expounds on the mutual dependence physiologically and influences pathologically between the organs [55]. Modern research has confirmed that gastrointestinal and respiratory mucosal tracts share the same origin and aspects of physiology and structure [56]. Consequently, as a specific axis with intensive dialogues, the GLA plays a vital role in functional structure, inflammatory response, and immunity between the intestine and lung [56, 57].

We revealed nine genera simultaneously expressing in the gut and lung, including *Bacteroides*, *Prevotella_9*, *Lactobacillus*, *Neisseria*, *Lachnospiraceae_NK4A136_group*, *Alloprevotella*, *Prevotellaceae_UCG-001*, *Prevotellaceae_NK3B31_group*, and *Ruminococcaceae_UCG-014*, among which connections exist. We noticed *Lactobacillus* decreased in the model group compared with the control group and increased after QBPF treatment in the gut and lung. As a probiotic, *Lactobacillus* has the protective effects of preventing asthma and anti-influenza in mice [58, 59]. A mixture of the six lactic acid bacteria from kefir increased the cytotoxicity of human natural killer KHYG-1 cells [60]. Our study suggested that pulmonary *Neisseria* decreased in the model group compared with the control group and increased after drug intervention. Commensal *Neisseria* plays a part in humans' evolution and stability of the upper respiratory tract microbiome[61]. *Lachnospiraceae* ferments different plant polysaccharides into SCFAs [62], and its proportion increases in the gut and lung after QBPF treatment. SCFAs are believed to have anti-inflammatory and immunomodulatory effects [63]. Our results showed that SCFAs-producing bacteria such as *Alloprevotella*, *Prevotellaceae_NK3B31_group*, and *Prevotellaceae UCG-001* decreased in the model group compared with the control group in the intestine and lung, suggesting disorders of fatty acids metabolism are involved in COPD. Studies have shown that *Ruminococcaceae* can also produce SCFAs and maintain a healthy gastrointestinal tract in individuals [64]. The relative abundance of *Ruminococcaceae_UCG-014* in the gut and lung increased after QBPF treatment compared with the model group.

In addition to the common bacteria, there were significant correlations between the different intestinal and pulmonary microbiota. *Mycoplasma* of pulmonary microbiota is positively correlated with *Prevotella_9* and *Bacteroide* and negatively correlated with *Prevotellaceae_NK3B31_group*, *Prevotellaceae_UCG-001*, and *Desulfovibrio* of intestinal microbiota. *Mycoplasma* belongs to the phylum *Tenericutes*. Their members establish symbiotic or highly toxic relationships in animals and humans [65]. *Prevotella* strains are associated with plant-rich diets in the gut, but they are also linked with chronic inflammatory conditions in mice and humans [66]. The results suggested that the imbalance of COPD microbiota is related to the increase of harmful bacteria and affect the production of SCFAs. The correlations between the intestinal and pulmonary microbiota add pieces of evidence to the GLA [67, 68].

Based on the changes in the community structure of intestinal and pulmonary microbiota, we performed COG and KEGG function prediction to analyse the differences in metabolic pathways among the three groups. The results showed that amino acid-related enzymes, aminoacyl-tRNA biosynthesis and purine metabolism pathway increased markedly in the model group compared with the control group but decreased observably after QBPF intervention. COPD patients are associated with amino acid metabolic deregulations [69, 70]. Studies have shown that serum histidine levels are elevated in COPD patients with worse disease severity with emphysema, cachexia and increased systemic inflammation [71, 72]. Cysteine, glycine, and glutamates increased in the lung of idiopathic pulmonary fibrosis compared with the control group [73]. Uric acid is the end product of purine metabolism, and the increased level of serum uric acid is thought to be a consequence of increased purine catabolism in the presence of tissue hypoxia [74]. Many patients with COPD have systemic hypoxia at rest or during acute exacerbation due to decreased oxygen diffusion capacity and alveolar hypoventilation. Therefore, serum uric acid is higher in patients with COPD [75]. These results may indicate that the imbalance of intestinal and pulmonary microflora may lead to metabolic disorders, affecting the occurrence and development of COPD.

Spearman correlation analysis showed that *Mycoplasma* in the lung was significantly positively associated with RORγt, IL-17A, and Th17/Treg, while dramatically negatively correlated with FEV 0.3/FVC, Foxp3 and IL-10. These further confirmed that *Mycoplasma* was a common pathogen. *Acetatifactor* in the intestine was notably negatively correlated with RORγt, IL-17A, and Th17/Treg and positively correlated with FEV 0.3/FVC, Foxp3, and IL-10. *Actatifactor* strongly correlates with steroid hormone biosynthesis, unsaturated fatty acid biosynthesis, linoleic acid metabolism and other metabolic pathways [76]. Similar to our results, the abundance of *Acetatifactor* in the microbial community of the lung cancer mice was relatively lower than that of the healthy control group [76]. *Coprococcus_2* of intestinal microbiota was significantly positively correlated with RORγt, IL-17A, and Th17/Treg and negatively correlated with FEV 0.3/FVC, Foxp3, and IL-10. Similarly, lung cancer patients with a relatively higher abundance of *Coprococcus* are prone to gastrointestinal reactions and disease progression after two cycles of chemotherapy [77]. Previous studies have confirmed that the community changes of intestinal and pulmonary microbiota in COPD are related to the decline of pulmonary function and immune imbalance in COPD patients and mice [39, 78, 79]. Consequently, our results further enrich the relationship between intestinal and pulmonary microbiota and pulmonary function and immune function in COPD model rats.

### There exists regulation of QBPF in immune homeostasis and intestinal and pulmonary microbiota in COPD model rats

Consistent with previous studies, the expression of CCL20, CCR6, and Th17 cells and related cytokines were increased, whereas Tregs and associated cytokines were reduced in the COPD model group compared with the control group, and QBPF treatment could alleviate the changes in these expression levels. These results suggested that QBPF treatment is conducive to the new balance of Th17/Treg.

Our results showed that *Mycoplasma* in the lung increased significantly in the COPD model group compared with the control group while decreased significantly after QBPF intervention. *Mycoplasma* is a notable species traditionally associated with infection [80], and *Mycoplasma pneumonia* is a common respiratory pathogen [81]. *Rikenellaceae_RC9_gut_group* in the lung decreased significantly in the COPD model group compared with the control group while increased significantly after QBPF intervention. Elevated gut microbiome abundance of *Rikenellaceae* is associated with reduced visceral adipose tissue and a healthier metabolic profile in the Italian elderly [82]. The relative abundance of *Coprococcus_2*, *Prevotella_9*, and *Blautia* in the gut increased significantly in the COPD group compared with the control group and reduced after QBPF treatment. *Coprococcus* is related to obese patients with Polycystic ovary syndrome is *Coprococcus_2* [83]. Some species of *Prevotella* have inflammatory properties in mice and humans [84] and may be involved in COPD clinically [11]. Specific operational taxonomic units in the *Blautia* are associated with inflammatory indicators in obese Children [85]. Therefore, these bacteria, which may be beneficial or harmful, are altered in the COPD model rats and modulated by QBPF. These results suggest that QBPF can regulate the composition of intestinal and pulmonary microbiota and improve community structure in COPD rats.

Many studies have shown that bioactive compounds or their metabolites from multiple herbs can inhibit COPD progression [86]. Previous clinical experiments showed that QBPF combined with Montelukast could down-regulate Th17 expression and up-regulate CD4^+^CD25^+^Foxp3^+^Treg expression in patients with AECOPD [35]. As the main drugs of QBPF, Radix Astragali and sun-dried ginseng exhibit anti-inflammatory and immunomodulatory properties and improve lung function in mice and rats [87–89]. Active ingredients isolated from other herbs in QBPF also have anti-inflammatory and immunomodulatory effects [90–94]. Our experiment confirmed that QBPF could improve the imbalance of Th17/Treg cells and regulate immune function. Additionally, through this experiment, we believe that the imbalance of intestinal and pulmonary microbiota may be one of the pathological mechanisms of COPD rats, and traditional Chinese medicine has a regulatory effect on the microbiota of the intestines and lungs in humans [95–97]. QBPF could treat COPD by regulating the intestinal and pulmonary microbiota, which provides a new idea and direction for exploring the potential biomarkers of COPD and the possible mechanism of QBPF in the prevention and treatment of COPD.

There are some limitations to our experiment. Firstly, we adopted a cross-sectional study to observe the changes in intestinal and pulmonary microbiota and immune homeostasis in control and model group rats. At the same time, we also confirmed the regulatory effect of QBPF on the microbiota of the intestine and lung and immune homeostasis in rats. However, the interaction mechanism between immune homeostasis and microbiota imbalance in COPD has not been specifically discussed. In addition, we should pay attention to the fact that the microbiota in the host is constantly changing under the influence of diet, environment, drugs and other factors, so we should control the uniqueness of variables to make the experimental results repeatable and comparable with the results of other similar studies. Our follow-up research direction is to carry out in vitro and in vivo colonization experiments on bacteria with significant differences between different groups, which is of great significance for developing new therapeutic approaches for COPD (Additional file 1).

## Conclusions

**Fig. 10:**
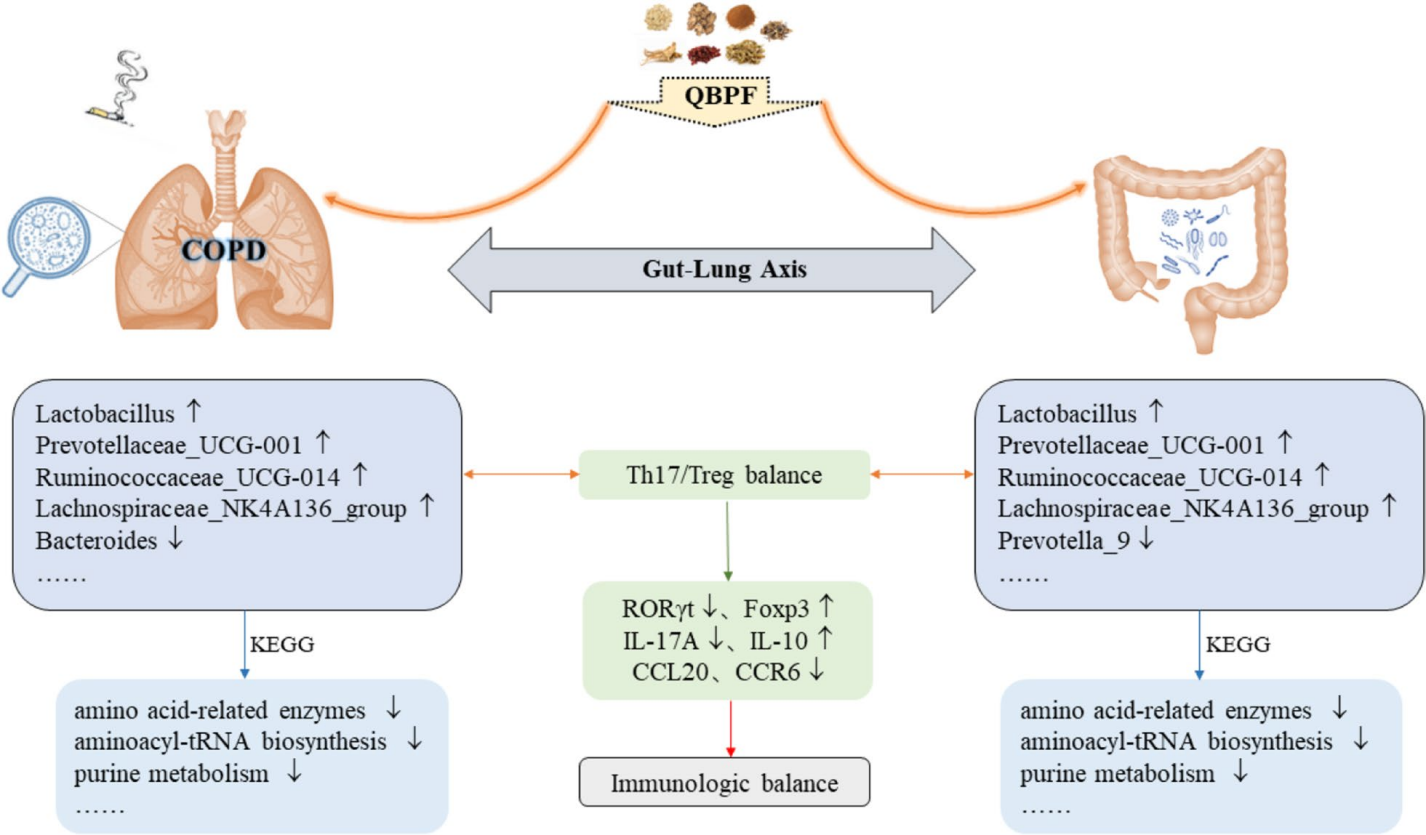
A model figure about the treatment of QBPF on COPD: we supposed that there is immune imbalance and microecological disorder in COPD. QBPF may up-regulate *Lactobacillus* and down-regulate *Bacteroides*, *Prevotella_9 *and other intestinal and pulmonary microbiota, regulate the level of Th17/Treg cells and the expression of related cytokines and transcription factors, so as to regulate immune homeostasis, ameliorate pulmonary inflammation and achieve the therapeutic effect on COPD

In summary, the present study demonstrates the imbalance of Th17/Treg and the dysbiosis of the abundance, diversity and community structure of intestinal and pulmonary microbiota in COPD model rats. The treatment of QBPF administration on COPD may be associated with maintaining the Th17/Treg balance and reshaping the intestinal and pulmonary microbiota. Moreover, we present shreds of evidence for the correlation between intestinal and pulmonary microbiota and the correlation between bacterial genera and pulmonary function and immune function. These findings may provide new insights into the potential biomarkers of COPD and the molecular mechanisms of QBPF administration (Fig. 10).

## Supplementary Information

**Additional file 1:** Raw data of UNIFI system about constituents of QBPF migrating to blood.

## Data Availability

All datasets generated for this study are included in the article.

## Abbreviations

COPD: Chronic obstructive pulmonary disease
Th: T helper
Treg: Regulatory T
QBPF: Qibai Pingfei Capsule
GLA: Gut–lung axis
AECOPD: Acute exacerbation of chronic obstructive pulmonary disease
FEV 0.3: Forced expiratory volume in 0.3 second
FVC: Forced vital capacity
SSC: Side scatter
FSC: Forward scatter
FITC: Fluorescein isothiocyanate
PE: Phycoerythrin
IL: interleukin
Foxp3: Forkhead box protein p3
ELISA: Enzyme-linked immunosorbent assay
CCL: CC chemokine ligand
CCR: CC chemokine receptor
SDS-PAGE: Sodium dodecyl sulphate–polyacrylamide gel electrophoresis
PVDF: Polyvinylidene fluoride
ROR: Retinoid related orphan receptor
GAPDH: Glyceraldehyde-3-phosphate dehydrogenase
HRP: Horseradish peroxidase
GC: Gut contents
BALF: Bronchoalveolar lavage fluid
ANOVA: One-way analysis of variance
LDA: Linear Discriminant Analysis
LEfSe: Linear Discriminant Analysis Effect Size
PICRUSt: Phylogenetic Investigation of Communities by Reconstruction of Unobserved States
COG: Clusters of orthologous groups
KEGG: Kyoto Encyclopedia of Genes and Genomes
SCFAs: Short-chain fatty acids
