# Supplementary material for: The treatment of Qibai Pingfei Capsule on chronic obstructive pulmonary disease may be mediated by Th17/Treg balance and gut-lung axis microbiota
Source: J Transl Med. 2022 Jun 21;20:281. doi: 10.1186/s12967-022-03481-w (PMC9210581; doi:10.1186/s12967-022-03481-w)
Supplement: Supplementary file 1 — Additional file 1: Raw data of UNIFI system about constituents of QBPF migrating to blood. [file 12967_2022_3481_MOESM1_ESM.docx]

Raw data about constituents of QBPF migrating to blood

After deleting the false positive results detected in blank plasma, the specific results of the raw UNIFI system analysis are as follows.

**Ginsenoside Rf**

Neutral mass (Da) 800.49221

Observed neutral mass (Da) 800.4899

Observed m/z 845.4881

Mass error (mDa) -2.3

Mass error (ppm) -2.7

Expected RT (min) 0

Observed RT (min) 50.53

Detector counts 411503

Response 308199

Adducts +HCOO


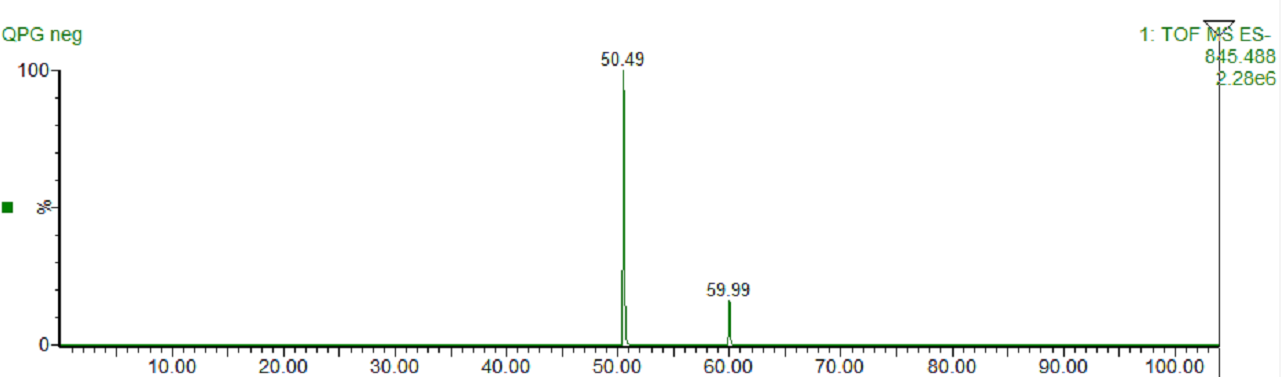

**Gypenoside XVII**

Neutral mass (Da) 946.55012

Observed neutral mass (Da) 946.549

Observed m/z 991.5472

Mass error (mDa) -1.1

Mass error (ppm) -1.1

Expected RT (min) 0

Observed RT (min) 50.4

Detector counts 375175

Response 264167

Adducts +HCOO, -H


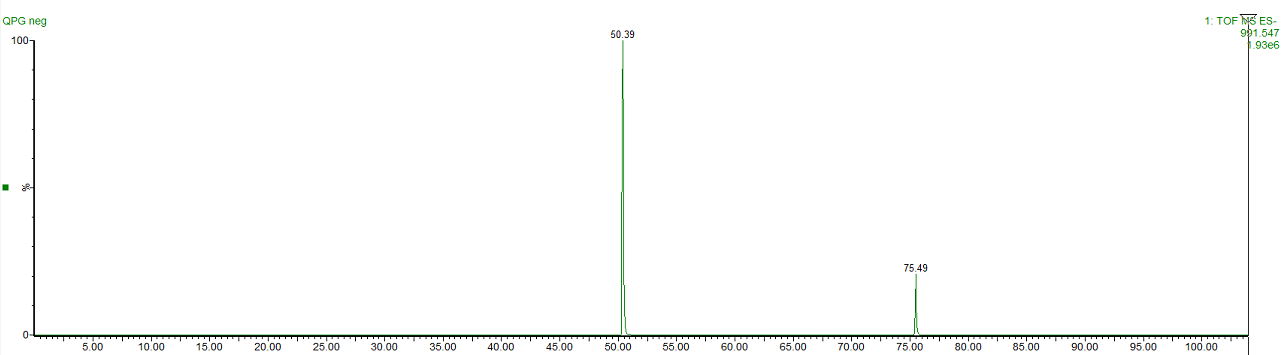

**Gypenoside XVII**

Neutral mass (Da) 956.49808

Observed neutral mass (Da) 956.4981

Observed m/z 955.4908

Mass error (mDa) 0

Mass error (ppm) 0

Expected RT (min) 0

Observed RT (min) 69.12

Detector counts 212198

Response 155813

Adducts -H, +HCOO

**Ginsenoside Rb1**

Neutral mass (Da) 1108.60294

Observed neutral mass (Da) 1108.6033

Observed m/z 1153.6015

Mass error (mDa) 0.4

Mass error (ppm) 0.3

Expected RT (min) 0

Observed RT (min) 70.4

Detector counts 93766

Response 64969

Adducts +HCOO, -H

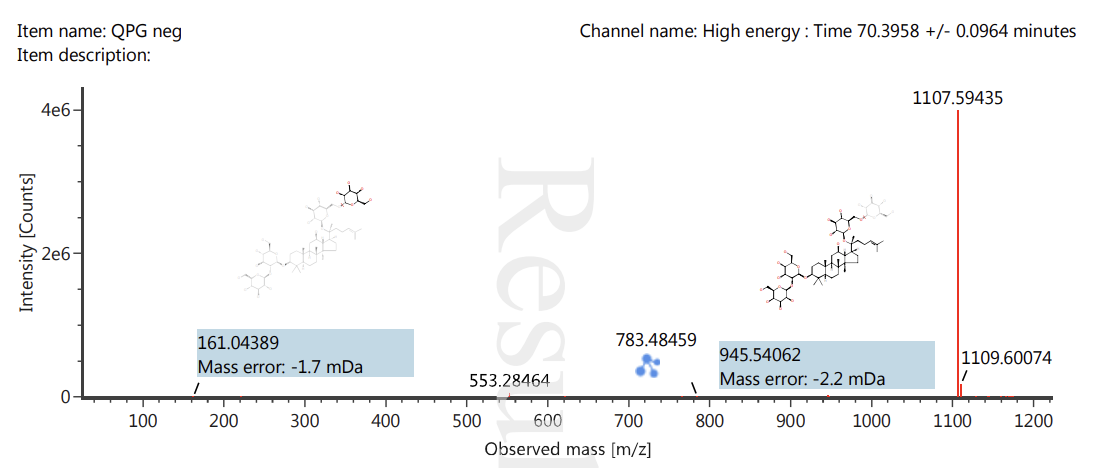


**Ginsenoside Rc**

Neutral mass (Da) 1078.59237

Observed neutral mass (Da) 1078.5923

Observed m/z 1123.5906

Mass error (mDa) 0

Mass error (ppm) 0

Expected RT (min) 0

Observed RT (min) 72.73

Detector counts 80504

Response 57316

Adducts +HCOO, -H

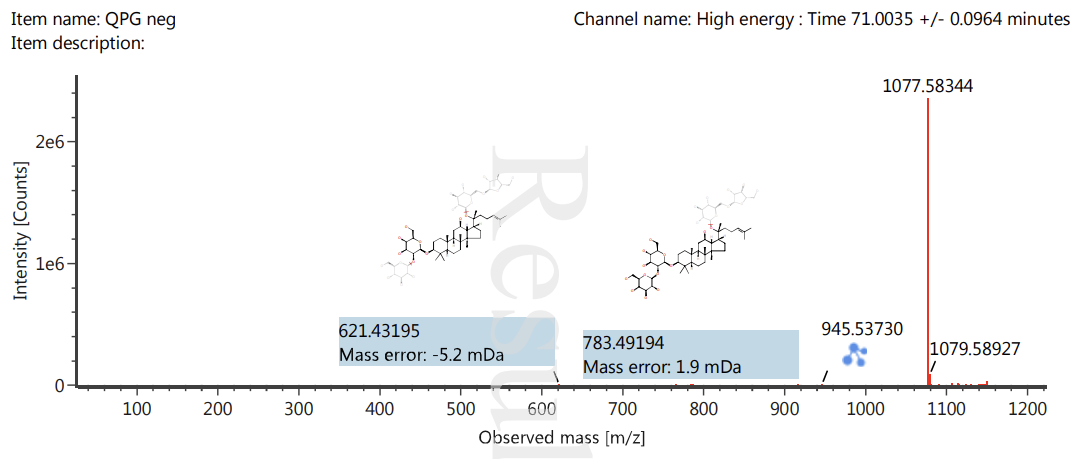


**Malonyl-ginsenoside Rb1**

Neutral mass (Da) 1194.60333

Observed neutral mass (Da) 1194.6044

Observed m/z 1193.5971

Mass error (mDa) 1.1

Mass error (ppm) 0.9

Expected RT (min) 0

Observed RT (min) 69.45

Detector counts 85304

Response 52400

Adducts -H

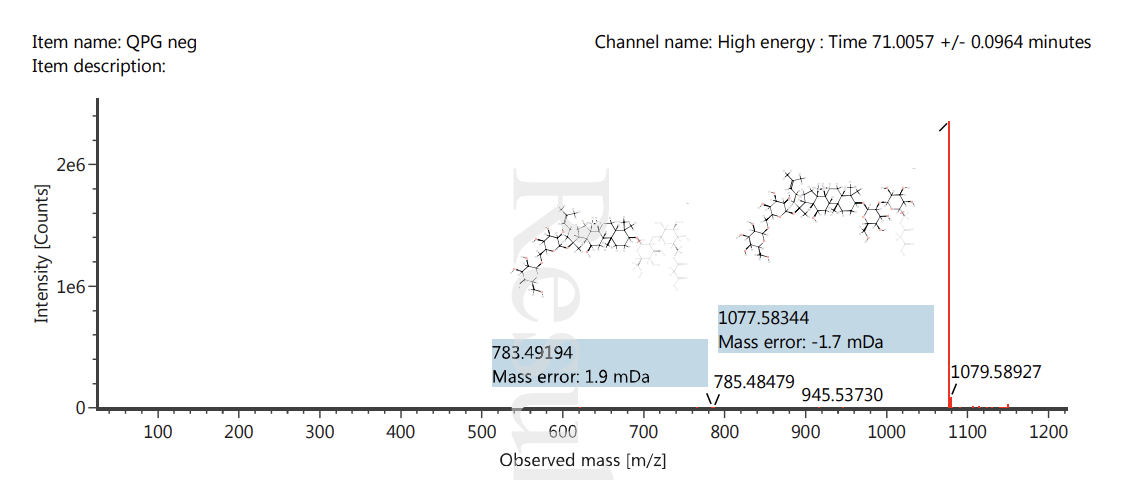


**Ginsenoside Rg1**

Neutral mass (Da) 800.49221

Observed neutral mass (Da) 800.4921

Observed m/z 845.4903

Mass error (mDa) -0.2

Mass error (ppm) -0.2

Expected RT (min) 0

Observed RT (min) 60.03

Detector counts 57622

Response 47238

Adducts +HCOO, -H

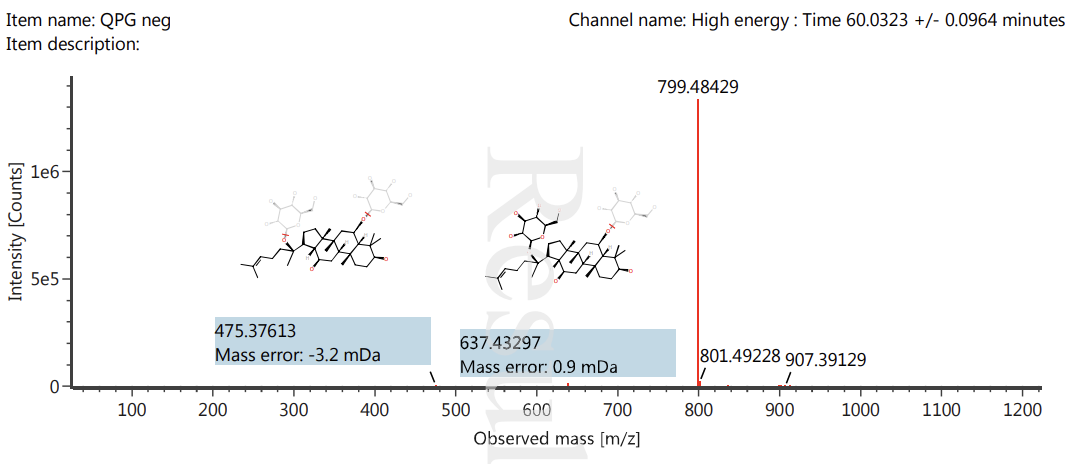


**Gypenoside XVII**

946.55012 946.5502 991.5484 0.1 0.1 0 75.51 58390 44729 +HCOO, -H

Neutral mass (Da)

Observed neutral mass (Da)

Observed m/z

Mass error (mDa)

Mass error (ppm)

Expected RT (min)

Observed RT (min)

Detector counts

Response

Adducts


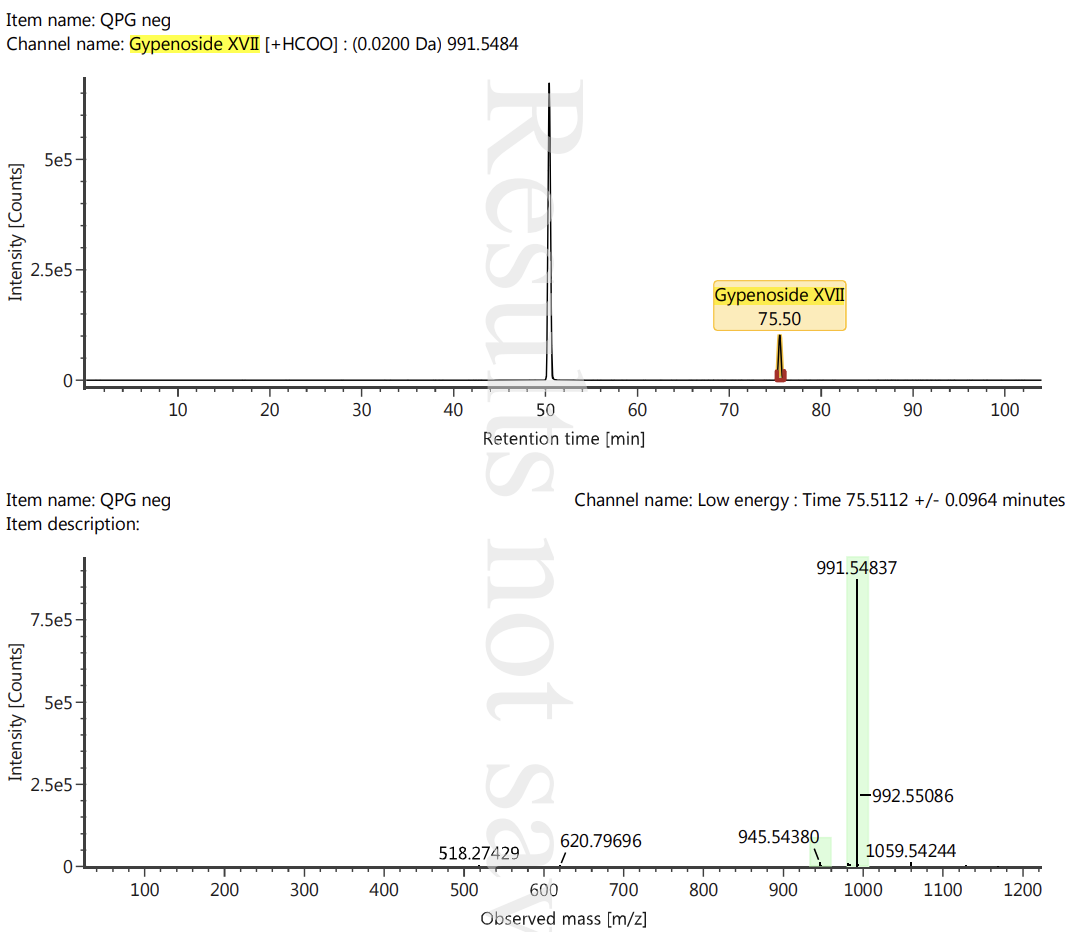


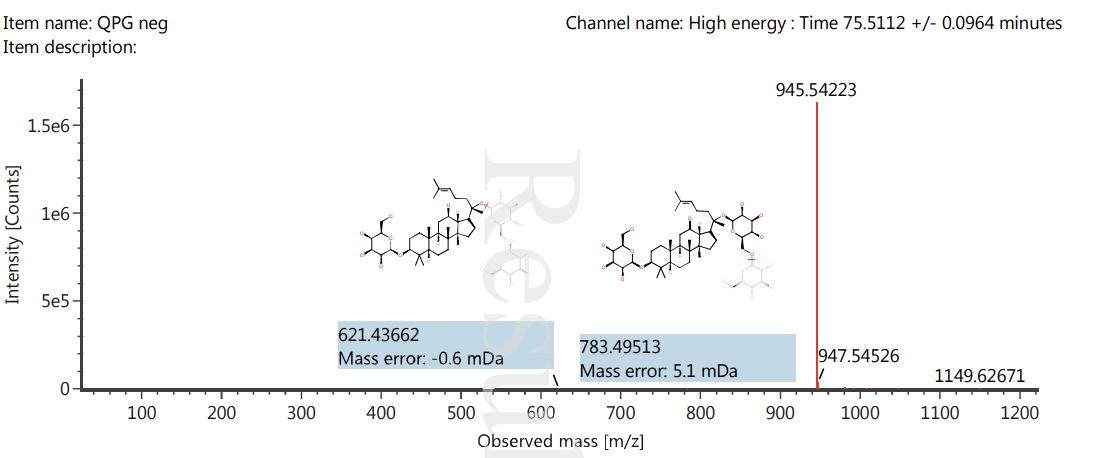


**Ginsenoside Rc**

Neutral mass (Da) 1078.59237

Observed neutral mass (Da) 1078.5913

Observed m/z 1123.5895

Mass error (mDa) -1.1

Mass error (ppm) -1

Expected RT (min) 0

Observed RT (min) 71

Detector counts 60429

Response 42611

Adducts +HCOO, -H


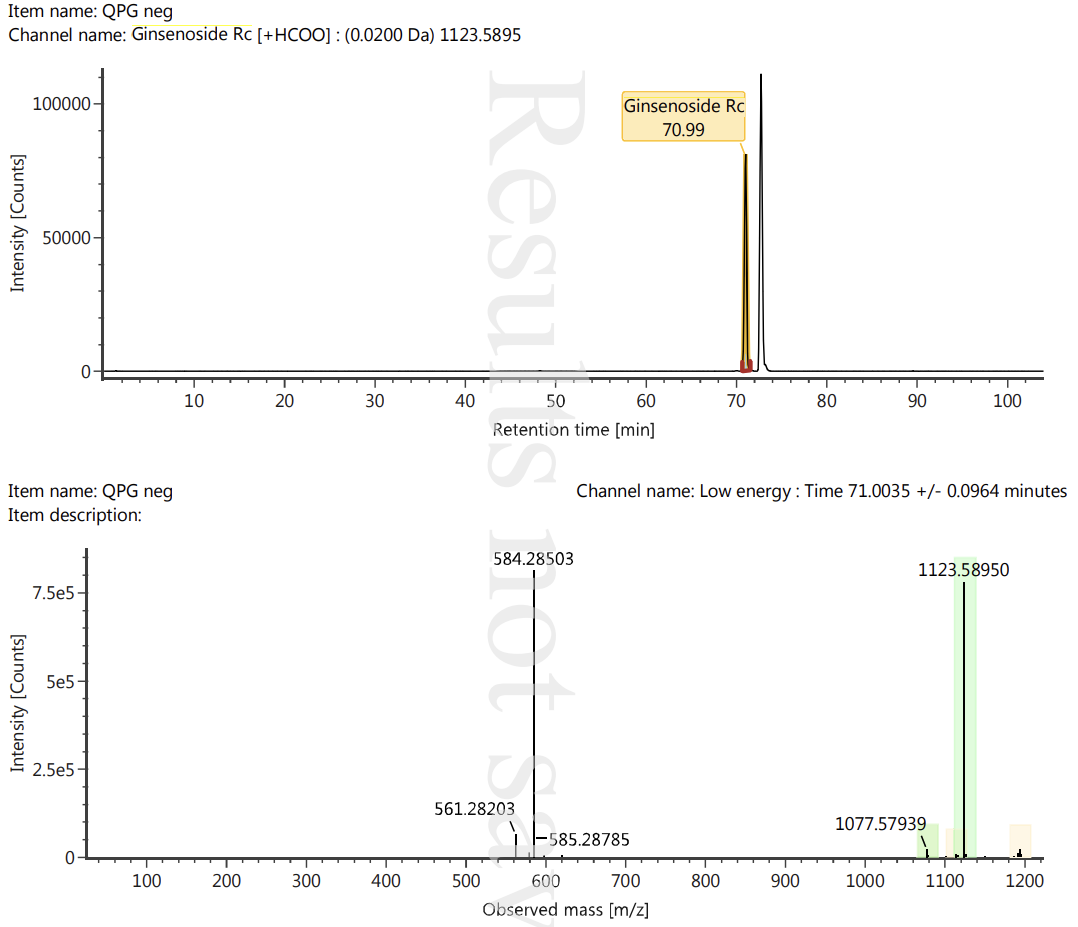


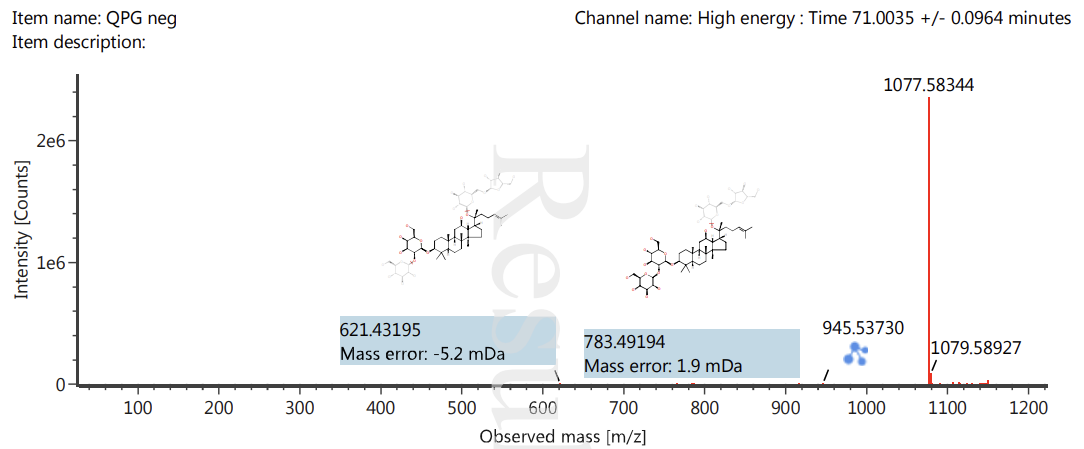


**Malonyl-ginsenoside Rb2**

Neutral mass (Da) 1164.59277

Observed neutral mass (Da) 1164.5926

Observed m/z 1163.5853

Mass error (mDa) -0.2

Mass error (ppm) -0.1

Expected RT (min) 0

Observed RT (min) 71.59

Detector counts 57426

Response 36380

Adducts -H


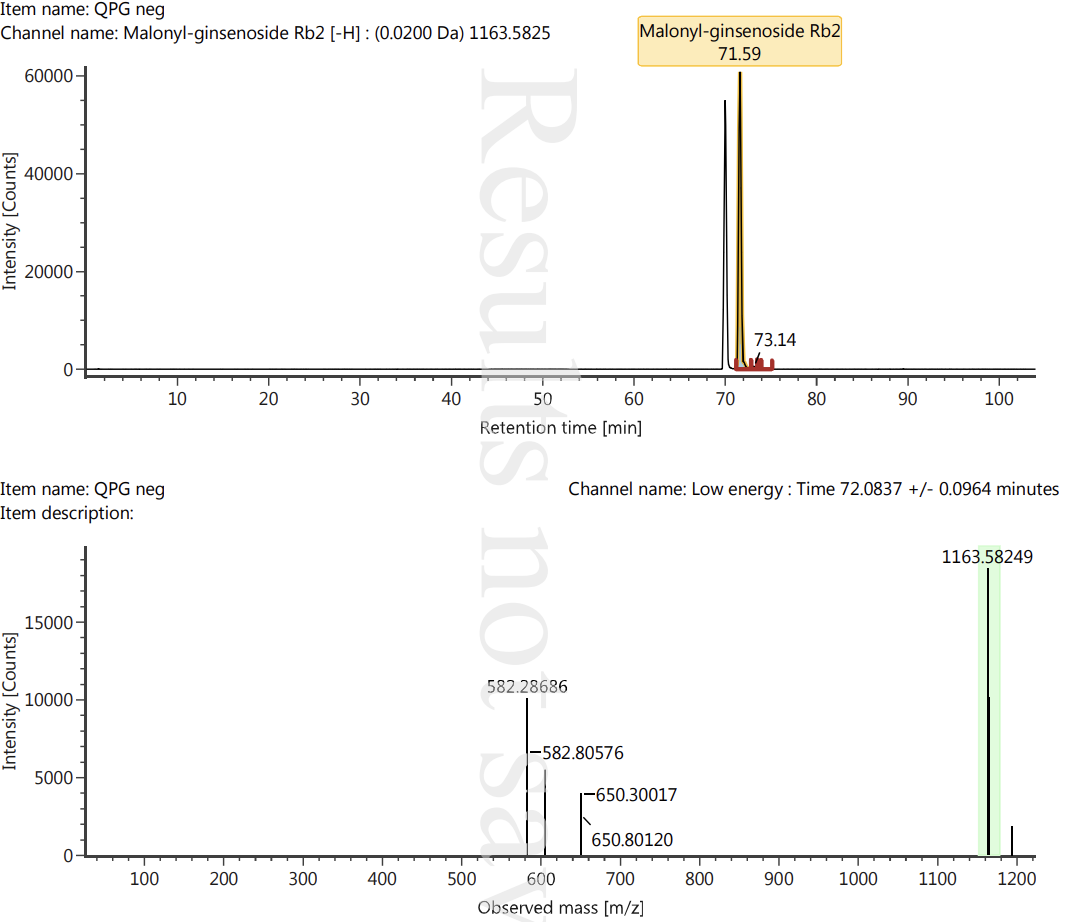


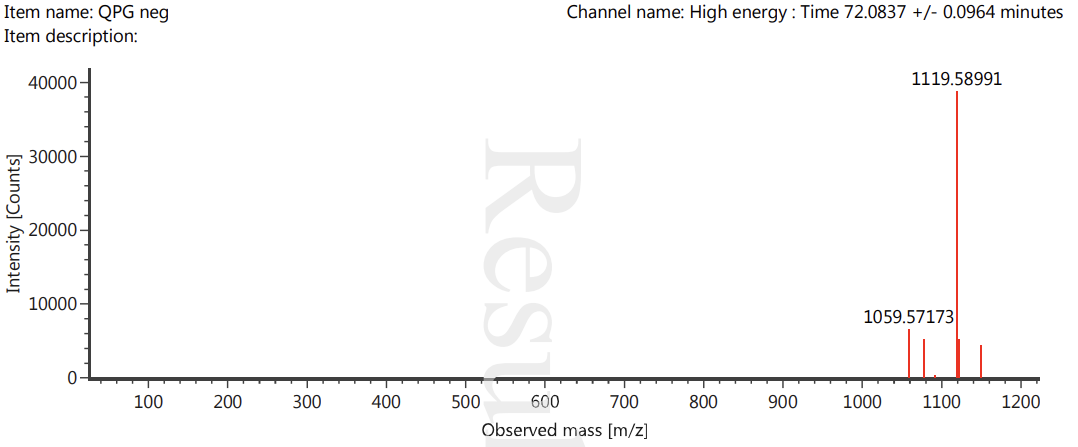


**Methyl palmitate**

Neutral mass (Da) 270.25588

Observed neutral mass (Da) 270.2542

Observed m/z 315.2524

Mass error (mDa) -1.7

Mass error (ppm) -5.3

Expected RT (min) 0

Observed RT (min) 79.05

Detector counts 14250

Response 13687

Adducts +HCOO


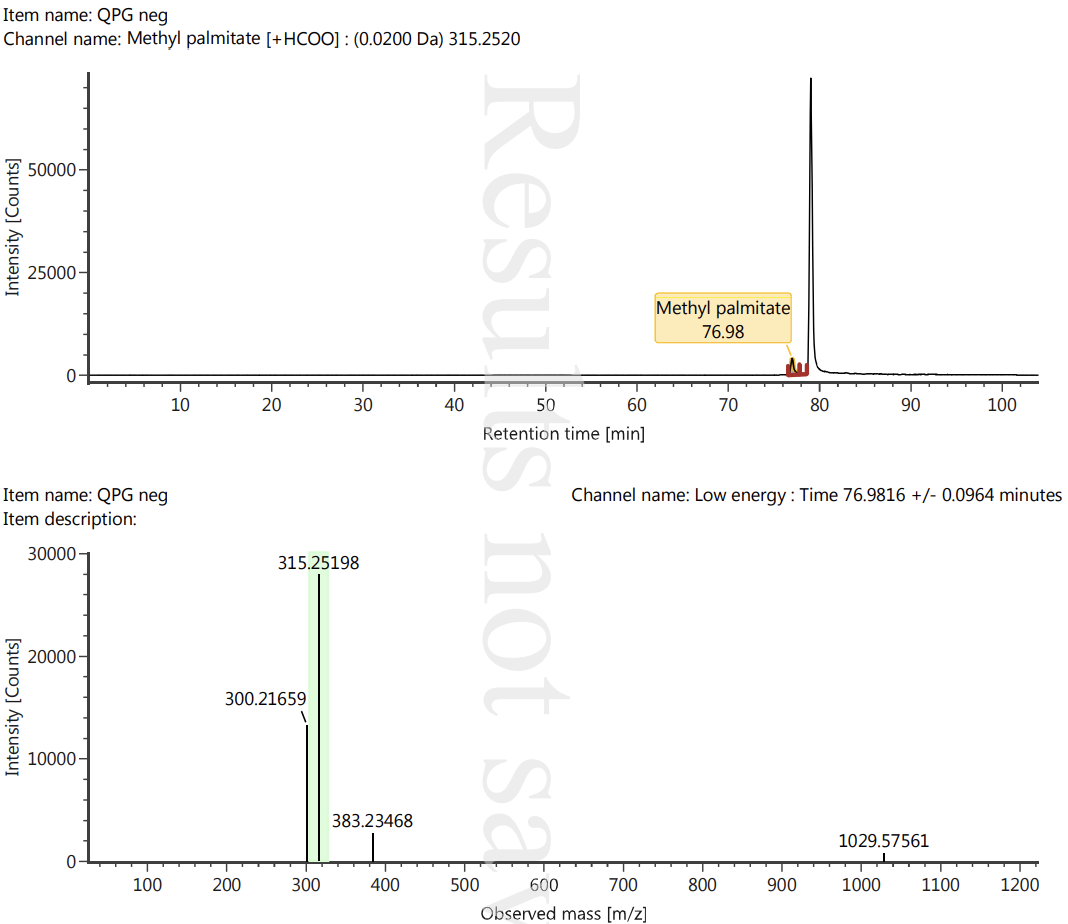


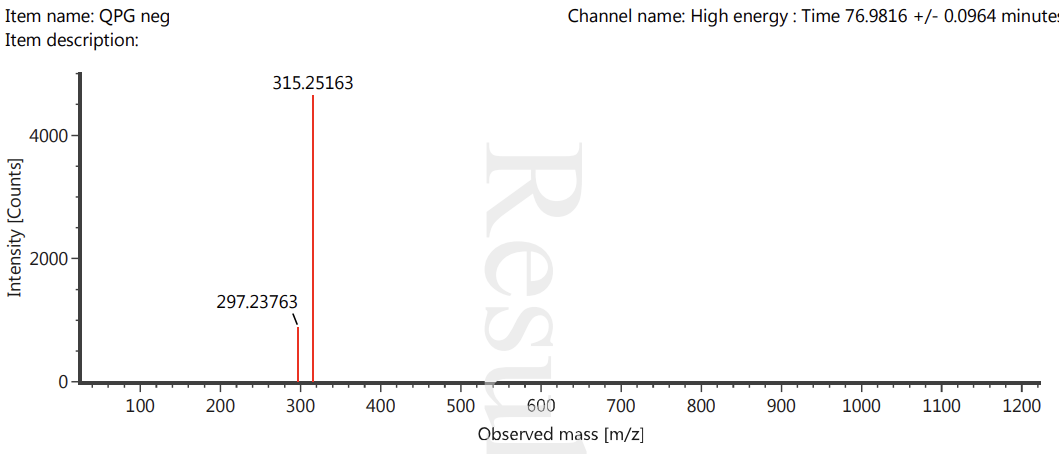


**Malonyl-floralginsenoside Re2**

Neutral mass (Da) 1032.55051

Observed neutral mass (Da) 1032.55

Observed m/z 1031.5427

Mass error (mDa) -0.5

Mass error (ppm) -0.5

Expected RT (min) 0

Observed RT (min) 74.15

Detector counts 19017

Response 12912

Adducts -H


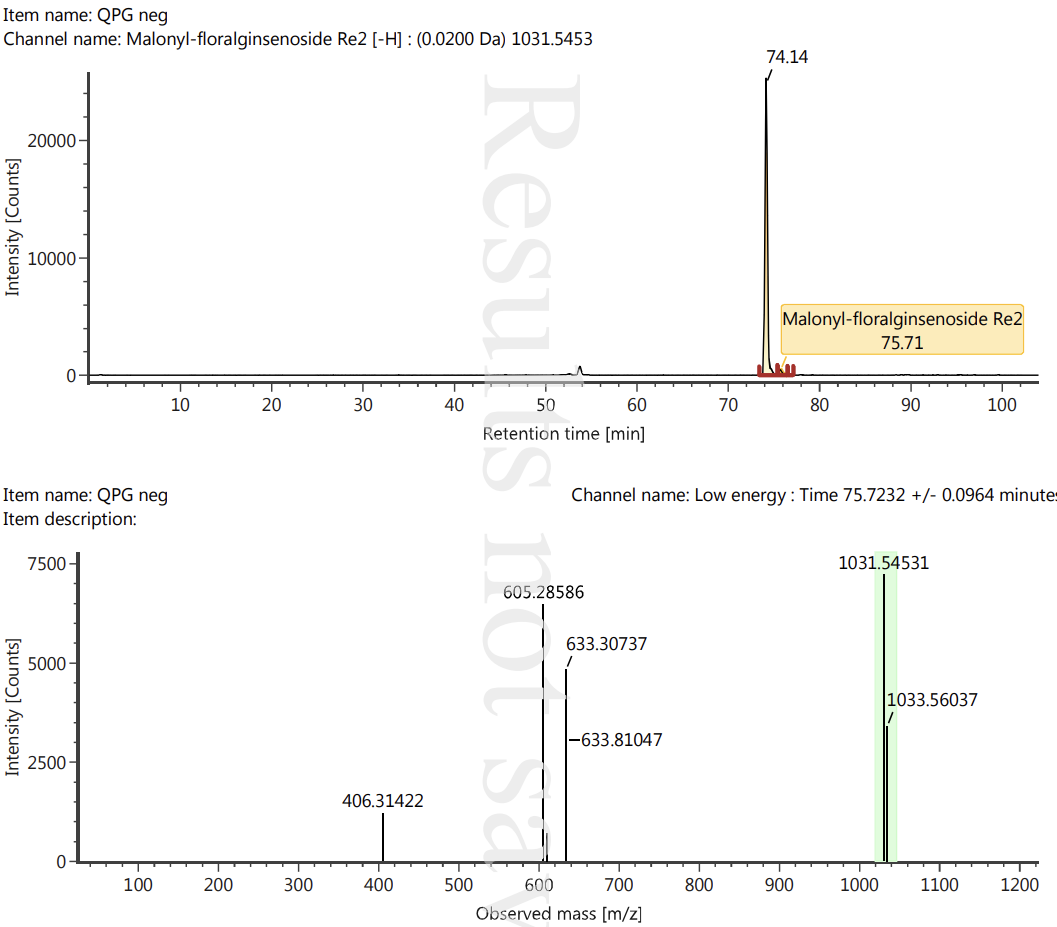


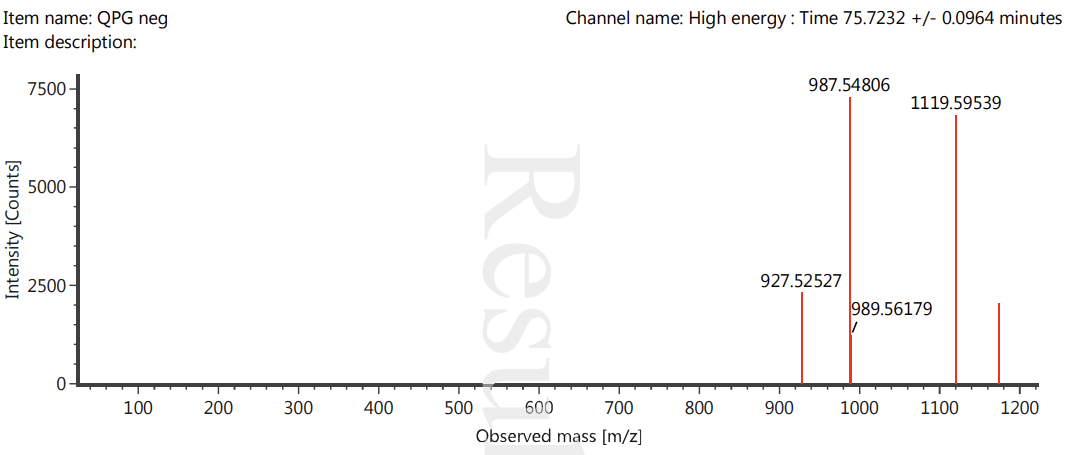


**20(s)-ginsenoside Rg3**

Neutral mass (Da) 784.49729

Observed neutral mass (Da) 784.4965

Observed m/z 829.4947

Mass error (mDa) -0.7

Mass error (ppm) -0.9

Expected RT (min) 0

Observed RT (min) 62.74

Detector counts 7960

Response 5887

Adducts +HCOO, -H


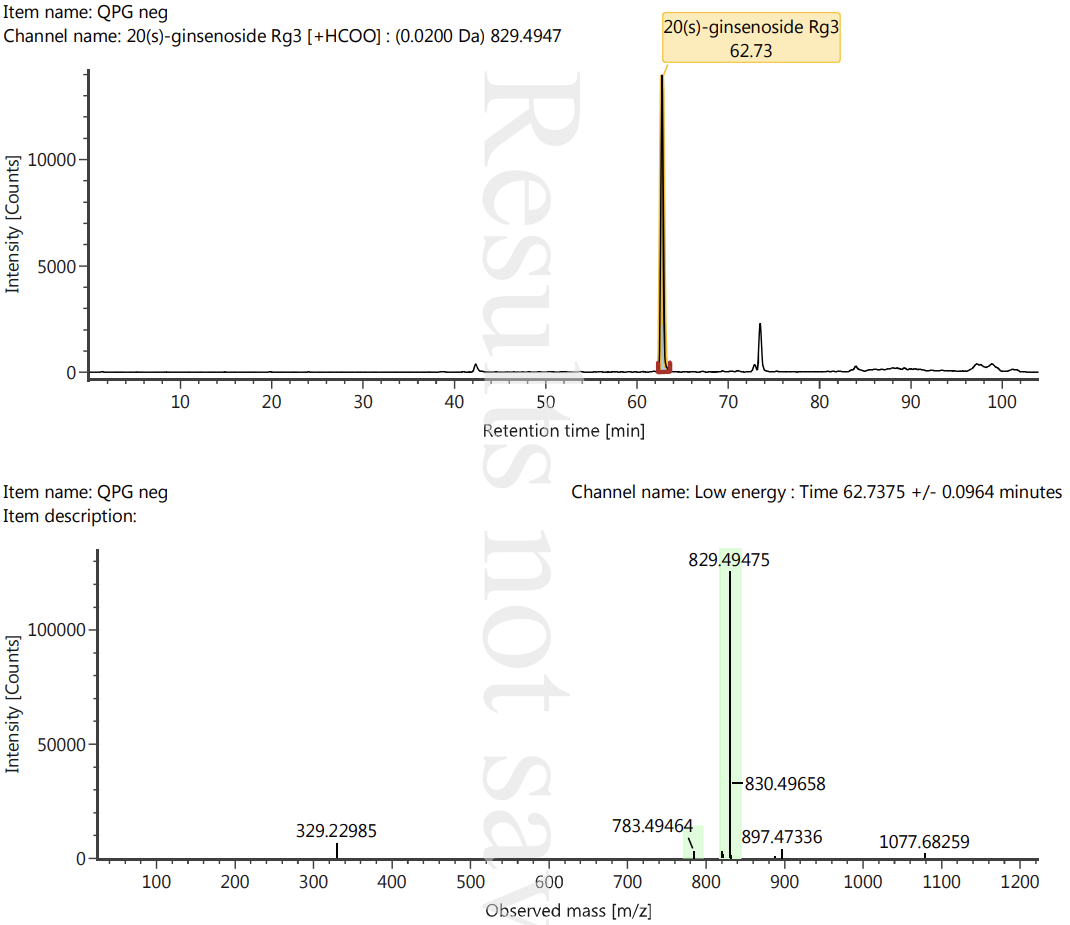


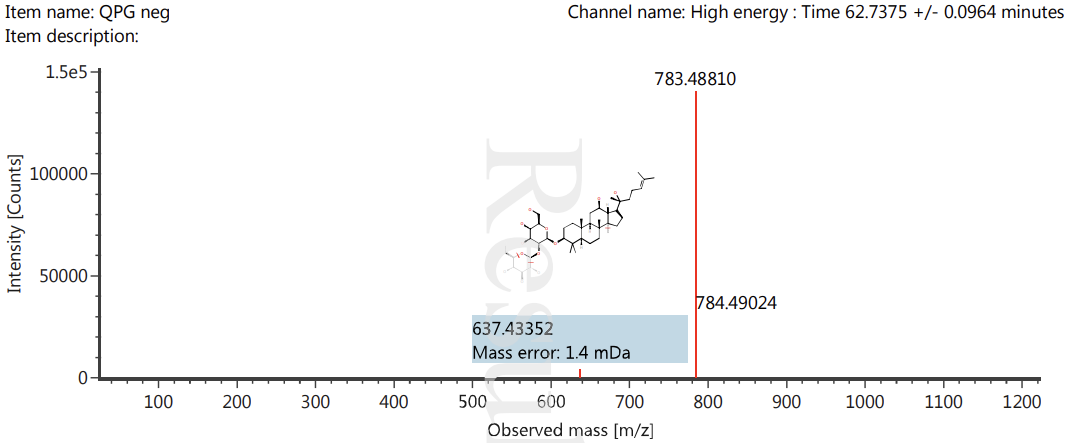


**20(R)-notoginsenoside R2**

Neutral mass (Da) 770.48164

Observed neutral mass (Da) 770.4799

Observed m/z 815.4781

Mass error (mDa) -1.8

Mass error (ppm) -2.2

Expected RT (min) 0

Observed RT (min) 60.78

Detector counts 6047

Response 4640

Adducts +HCOO


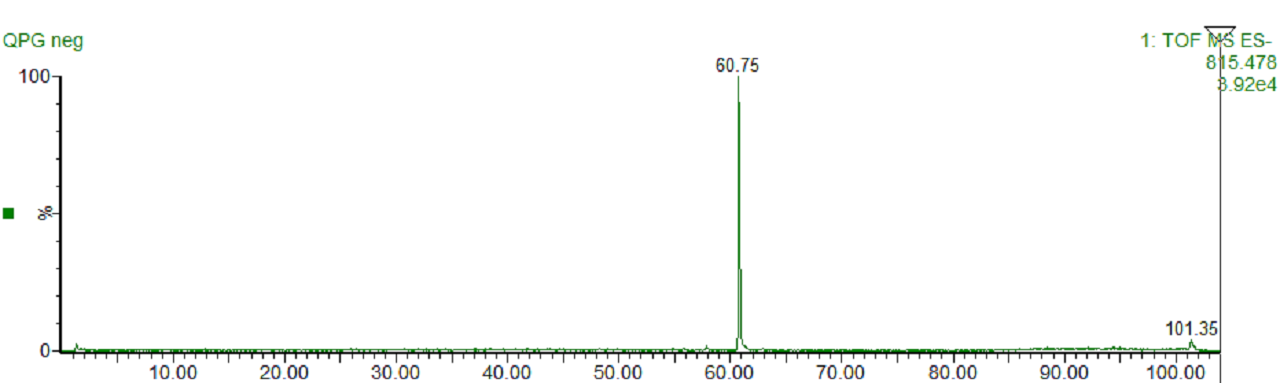

**Astragaloside III**

Neutral mass (Da) 784.46091

Observed neutral mass (Da) 784.4582

Observed m/z 829.4564

Mass error (mDa) -2.8

Mass error (ppm) -3.3

Expected RT (min) 0

Observed RT (min) 73.51

Detector counts 4958

Response 3710

Adducts +HCOO


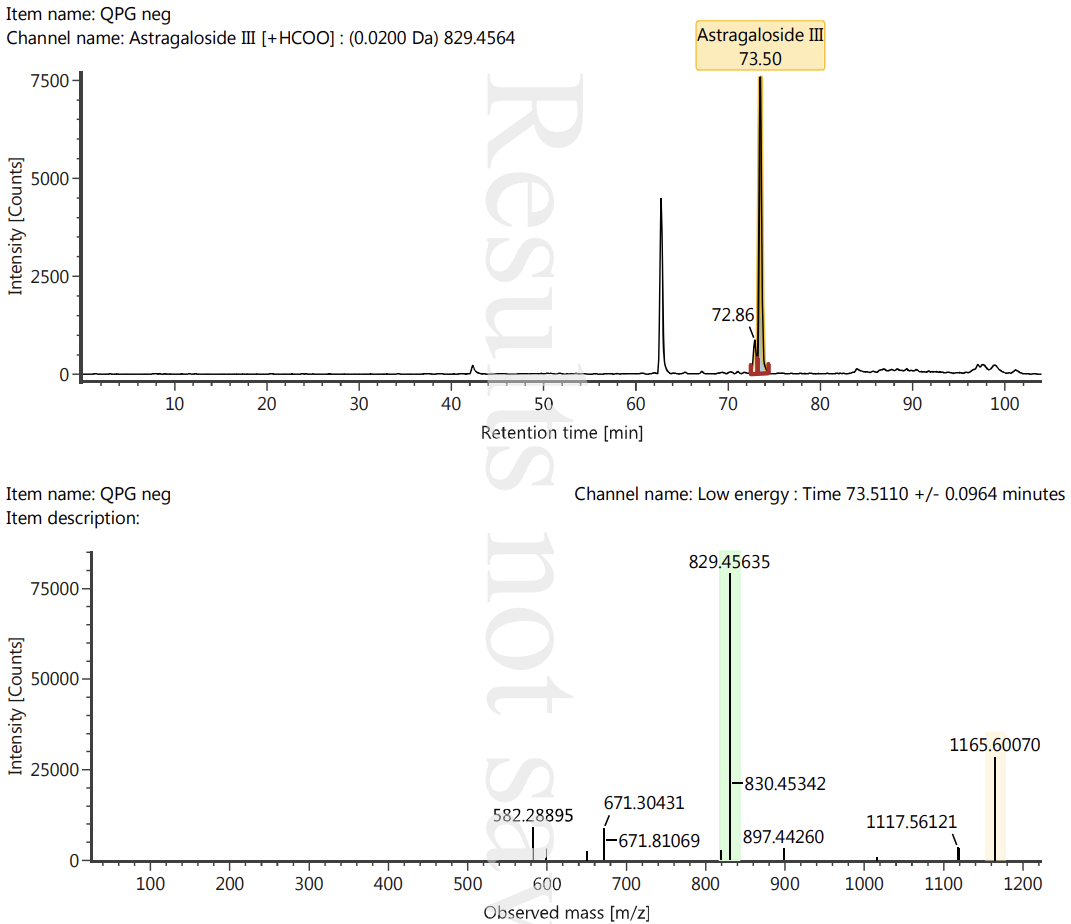


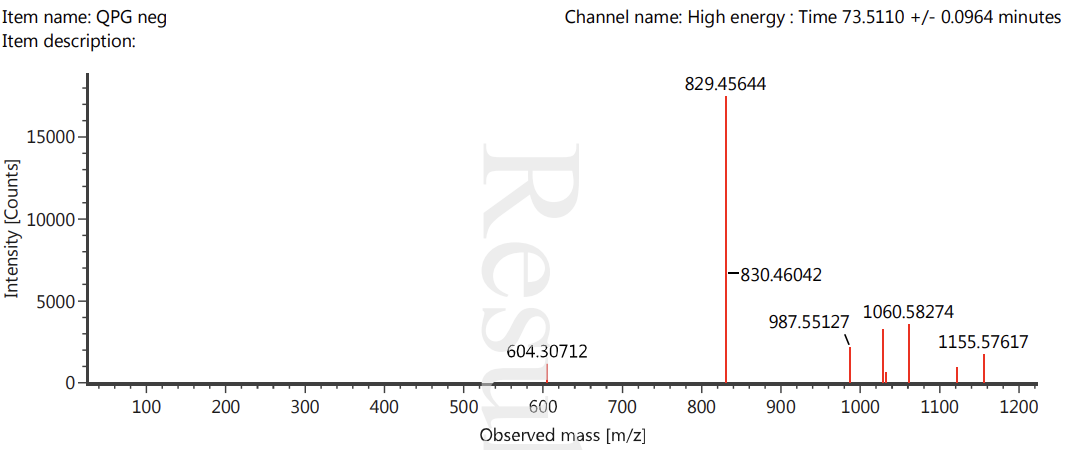


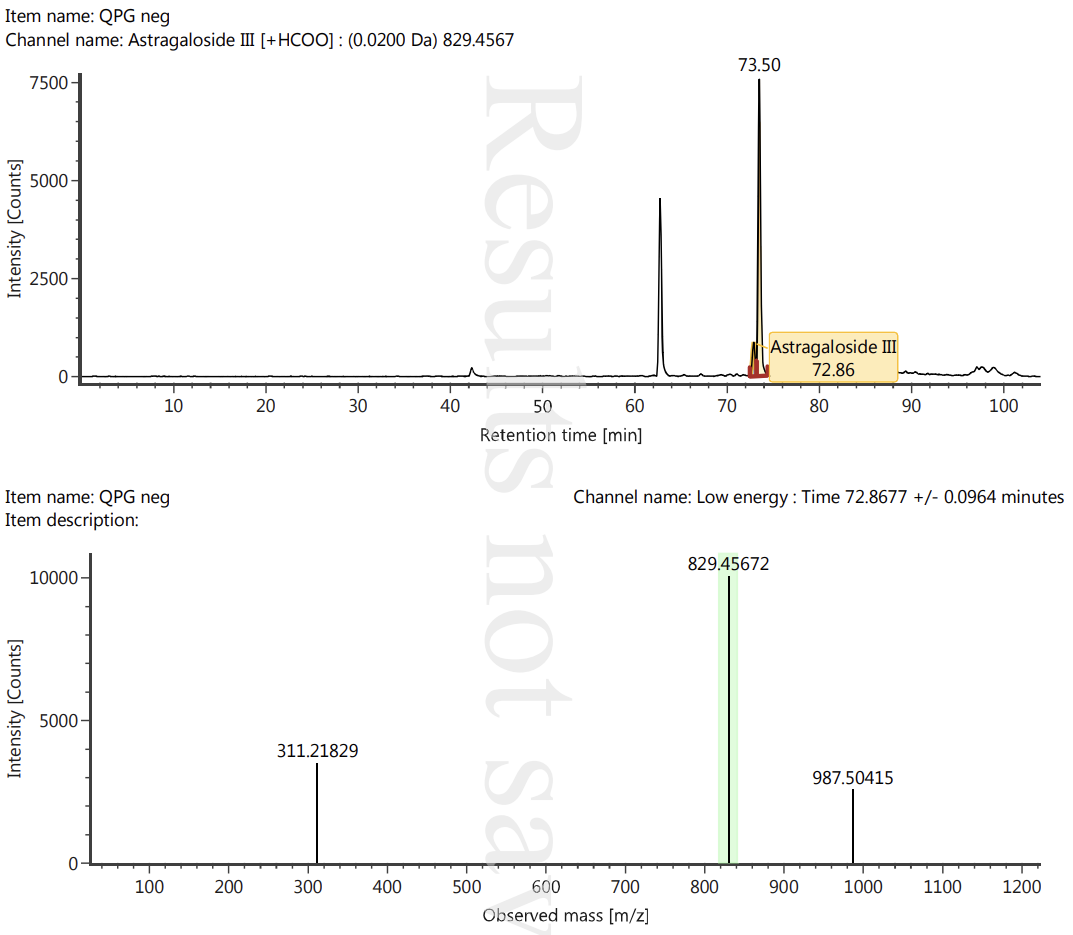


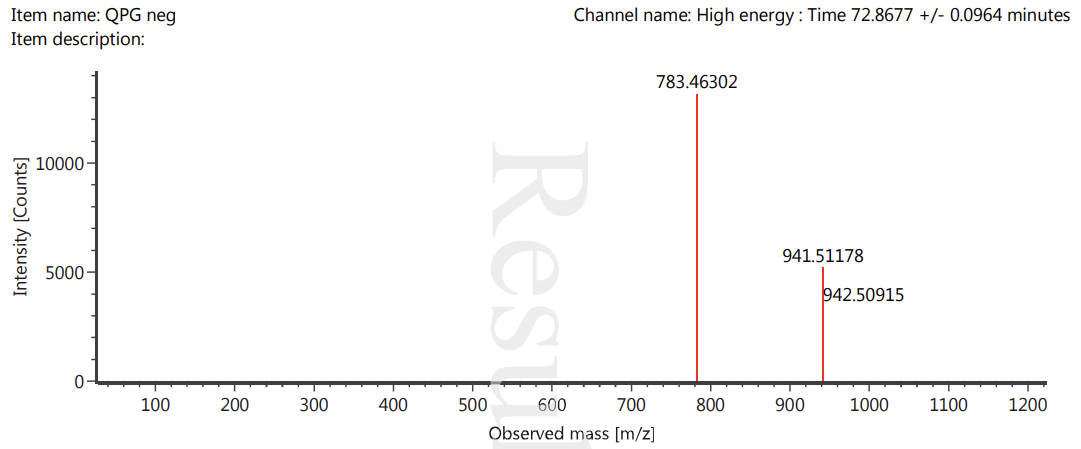


**Ginsenoside Rs1**

Neutral mass (Da) 1120.60294

Observed neutral mass (Da) 1120.601

Observed m/z 1165.5992

Mass error (mDa) -2

Mass error (ppm) -1.7

Expected RT (min) 0

Observed RT (min) 75.19

Detector counts 3808

Response 2159

Adducts +HCOO, -H


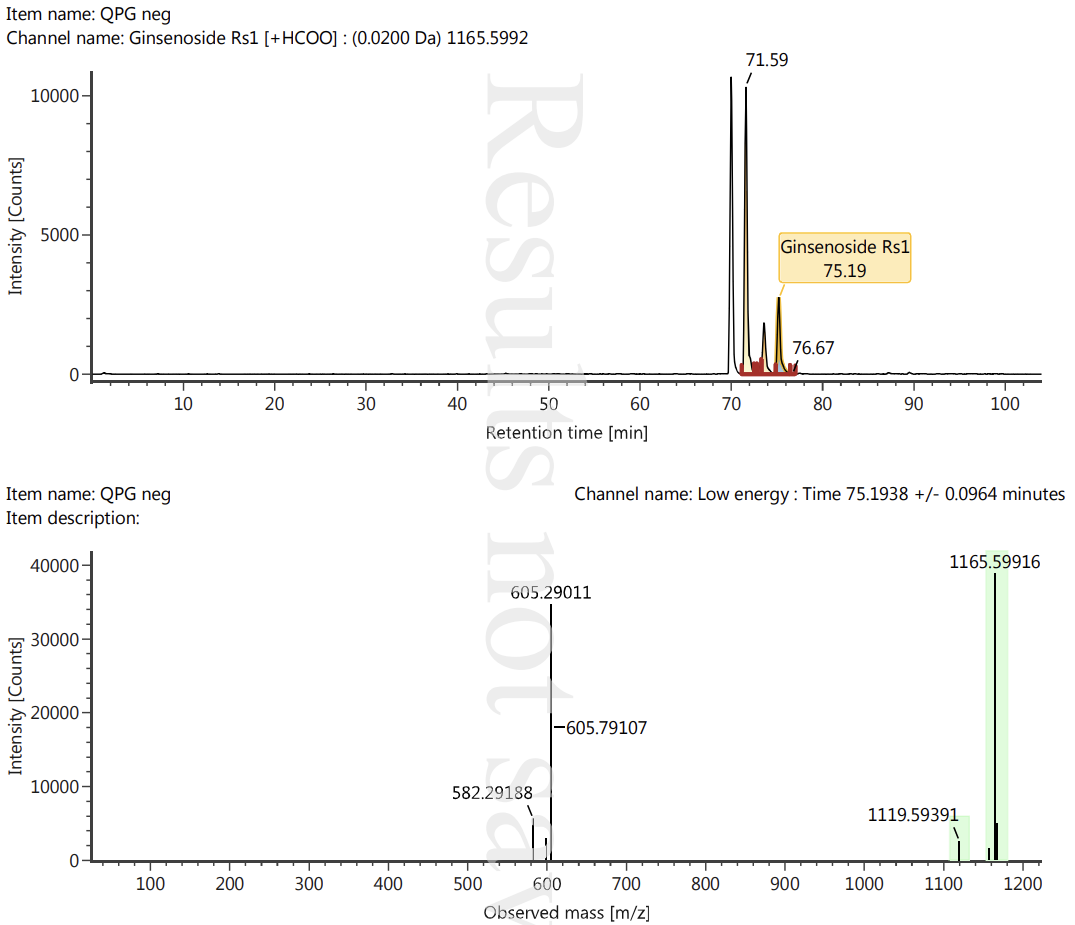


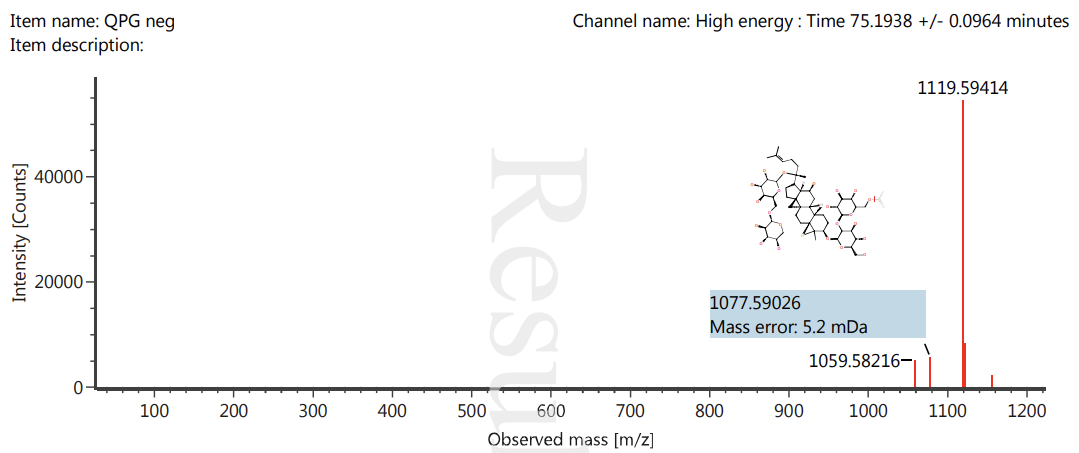


**Ginsenoside II**

Neutral mass (Da) 978.53995

Observed neutral mass (Da) 978.5377

Observed m/z 977.5304

Mass error (mDa) -2.2

Mass error (ppm) -2.3

Expected RT (min) 0

Observed RT (min) 47.89

Detector counts 2568

Response 1804

Adducts -H


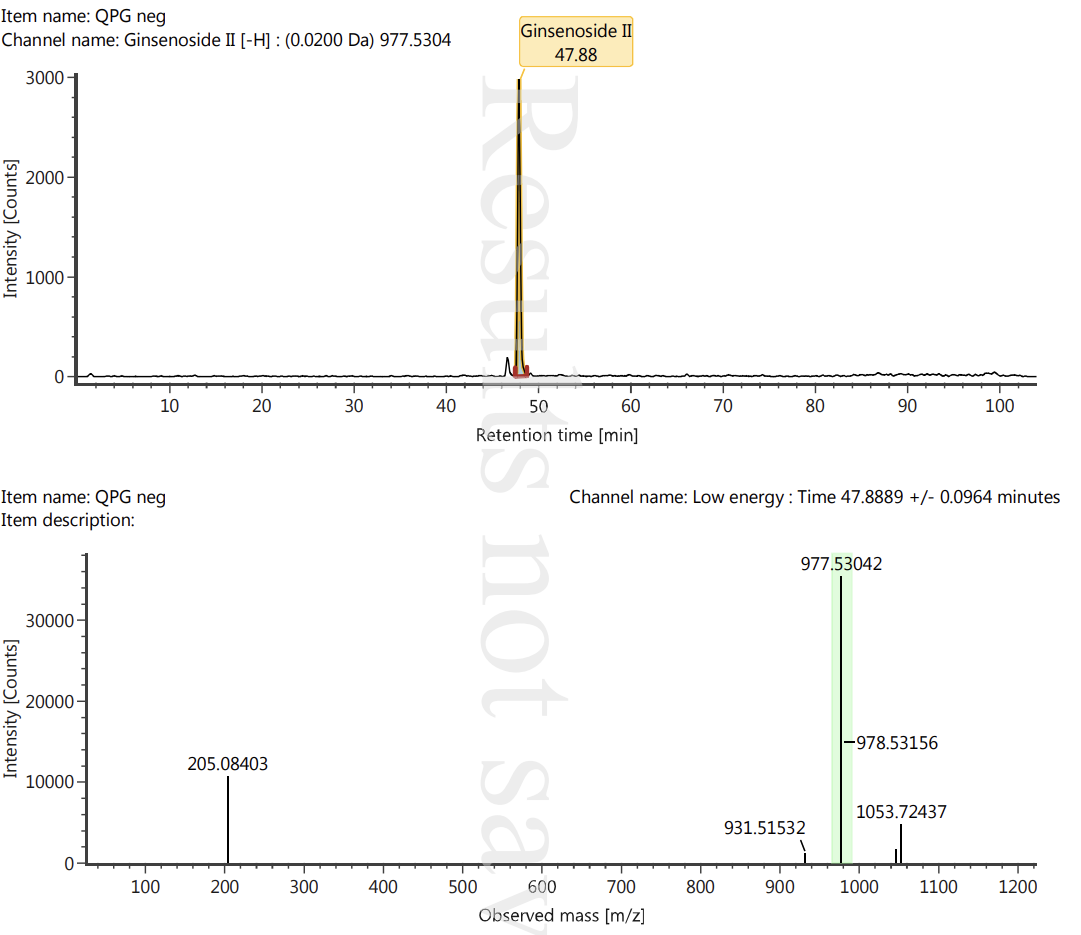


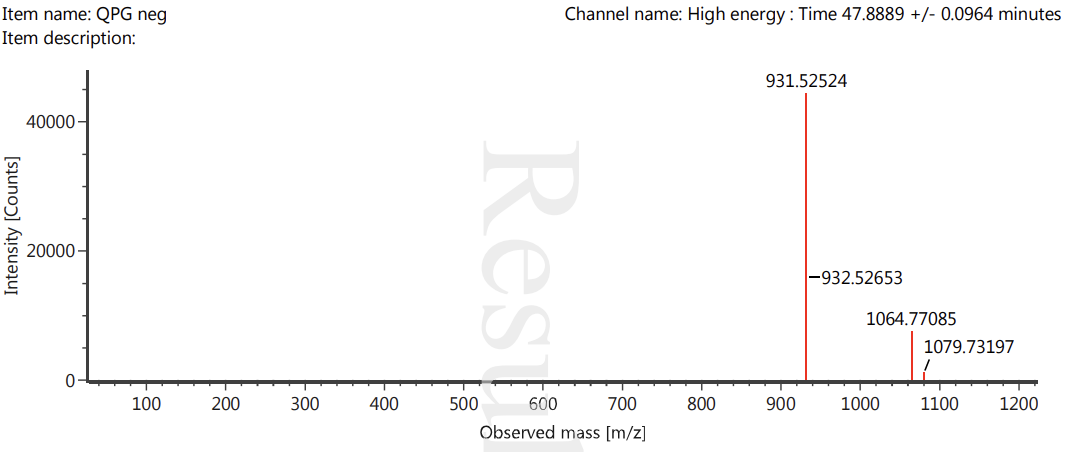


**Floralginsenoside O**

Neutral mass (Da) 1078.59237

Observed neutral mass (Da) 1078.5939

Observed m/z 1123.5921

Mass error (mDa) 1.5

Mass error (ppm) 1.3

Expected RT (min) 0

Observed RT (min) 73.23

Detector counts 3104

Response 1785

Adducts +HCOO, -H


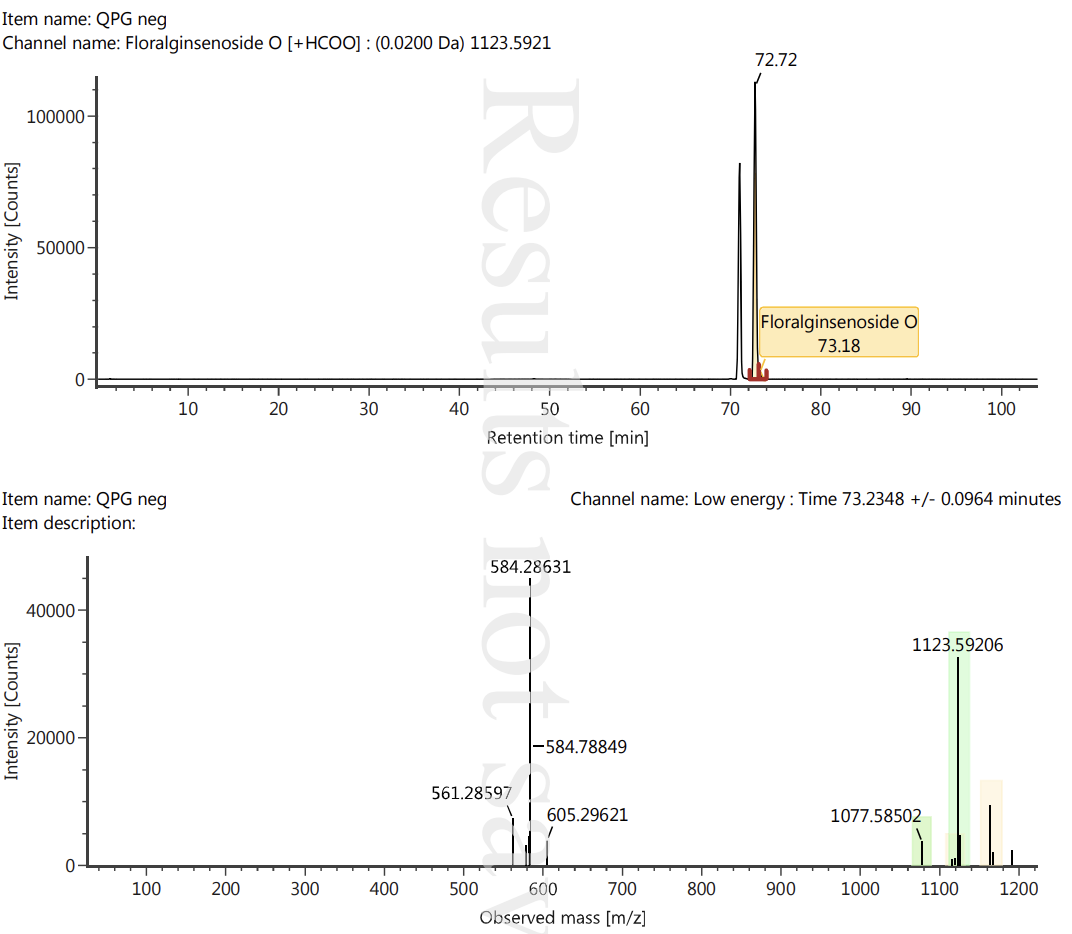

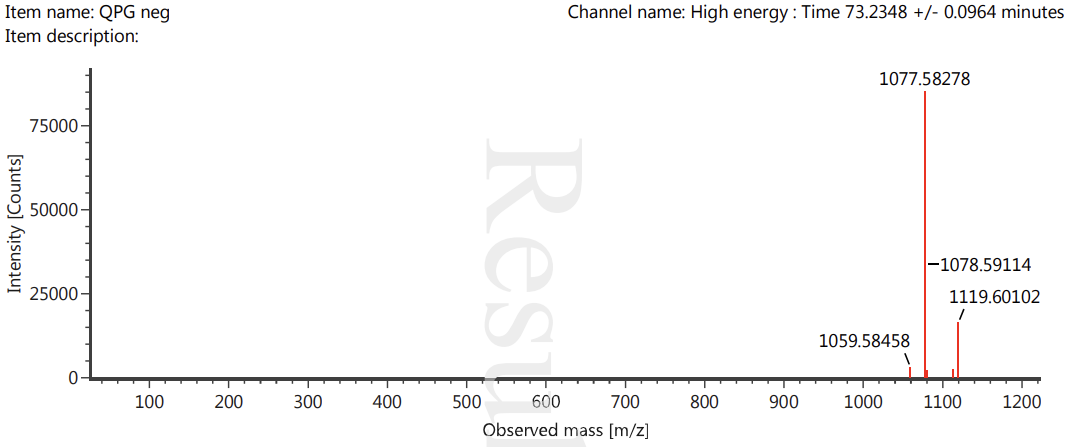


**Vinaginsenoside R8**

Neutral mass (Da) 962.54503

Observed neutral mass (Da) 962.5399

Observed m/z 1007.5381

Mass error (mDa) -5.1

Mass error (ppm) -5.1

Expected RT (min) 0

Observed RT (min) 47.21

Detector counts

Response 1667

Adducts +HCOO


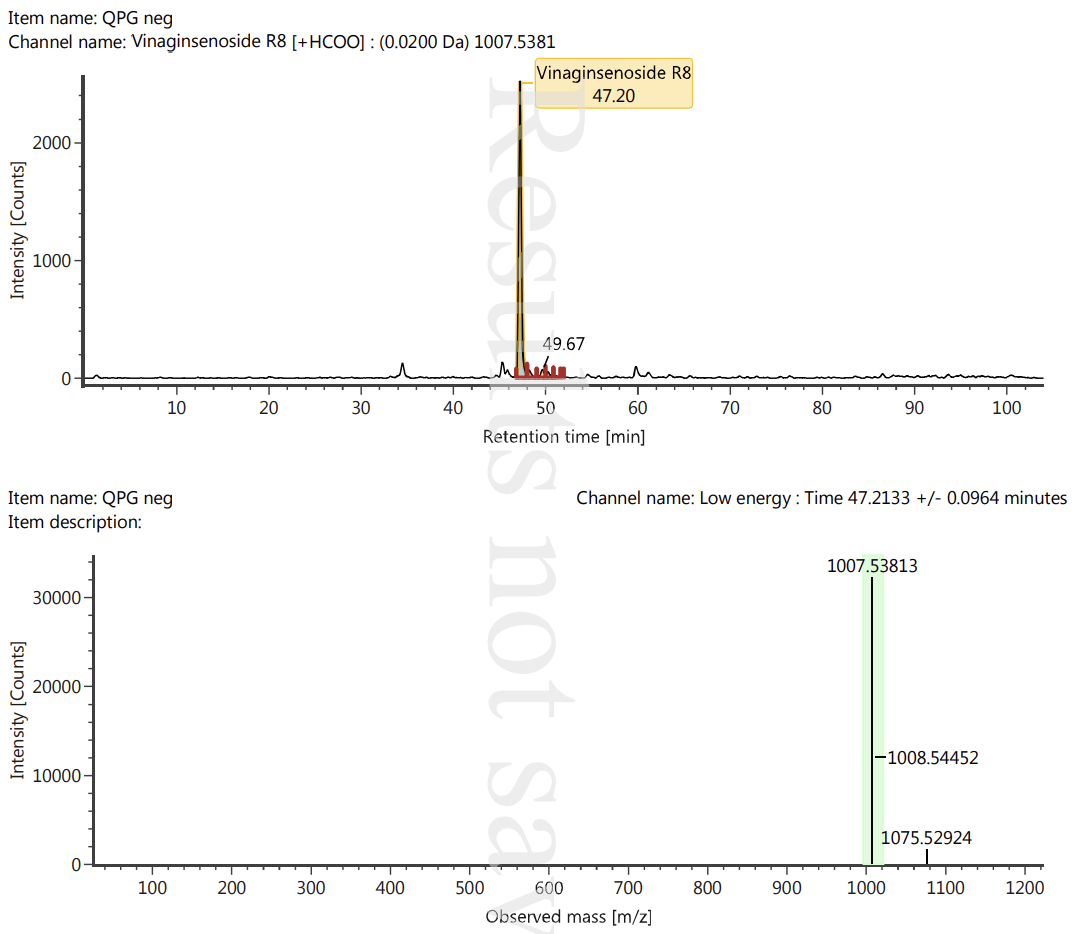


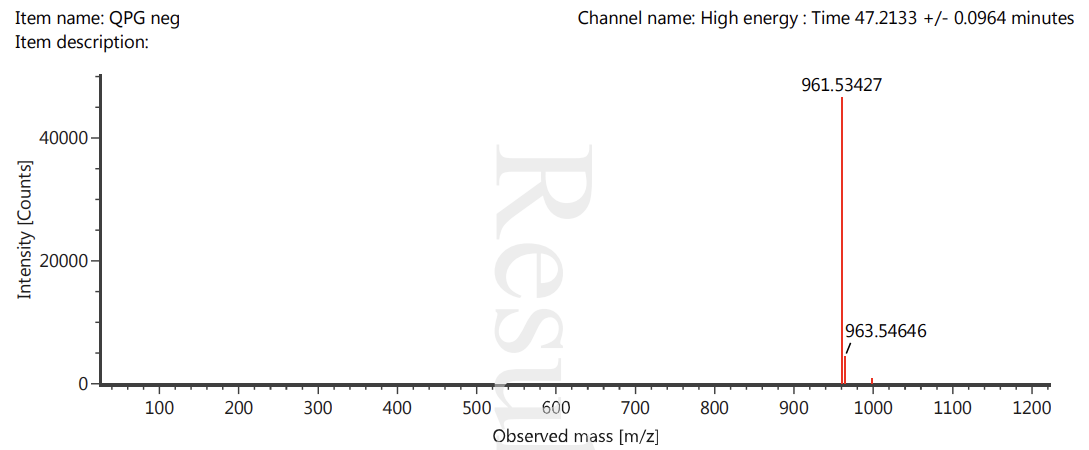


Isoastragaloside I

868.48204 868.4821 913.4803 0 0 0 77.96 2517 1660 +HCOO


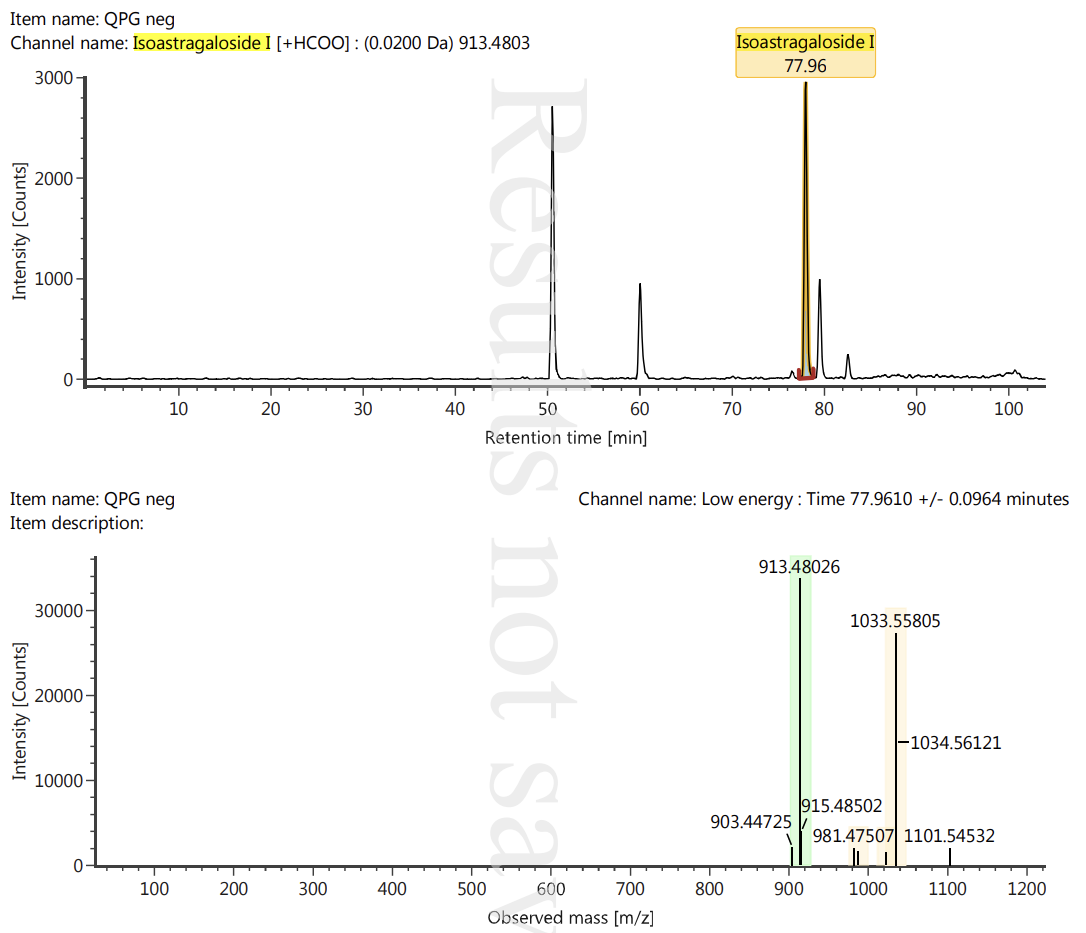


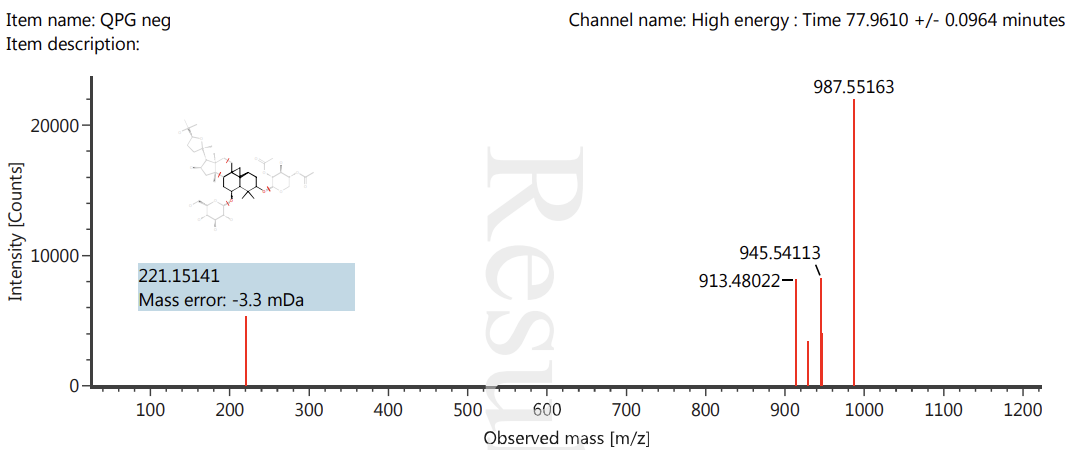


Isoastragaloside I

868.48204 868.4747 913.4729 -7.3 -8 0 50.53 2180 1589 +HCOO


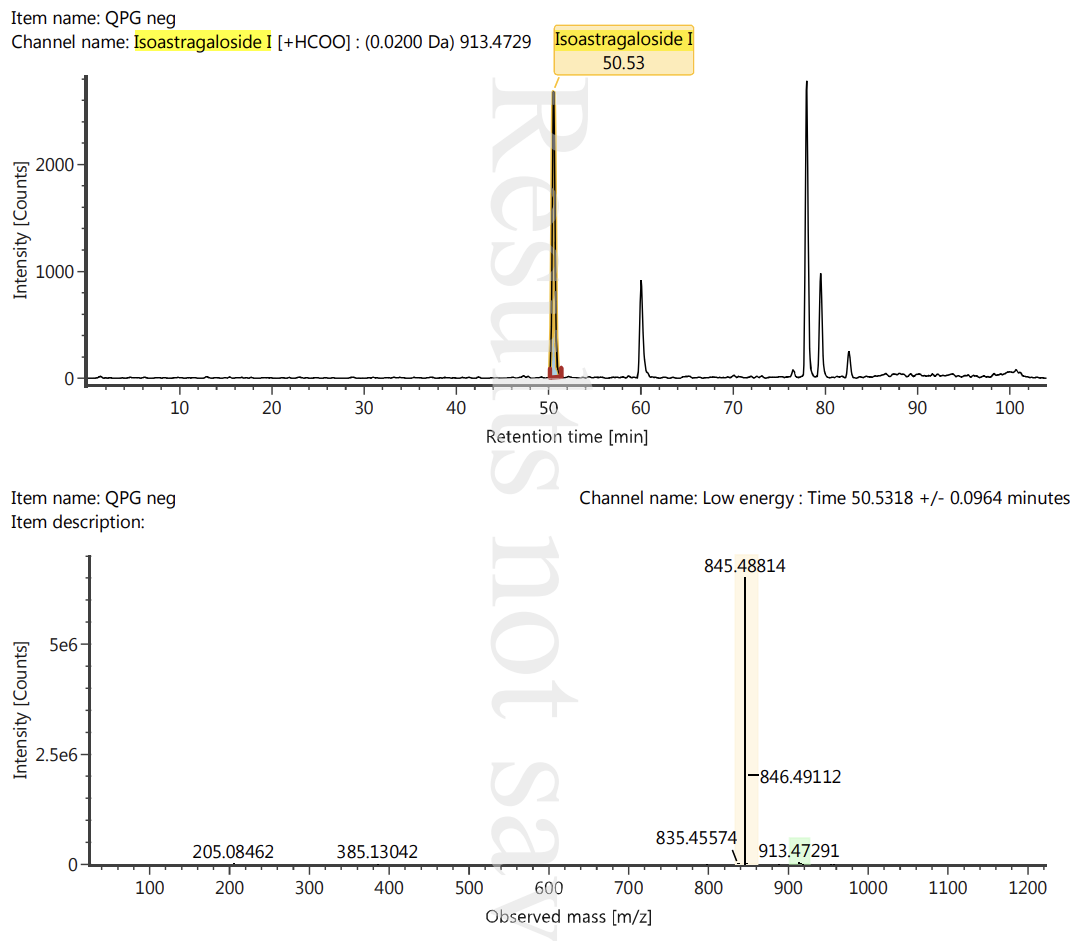


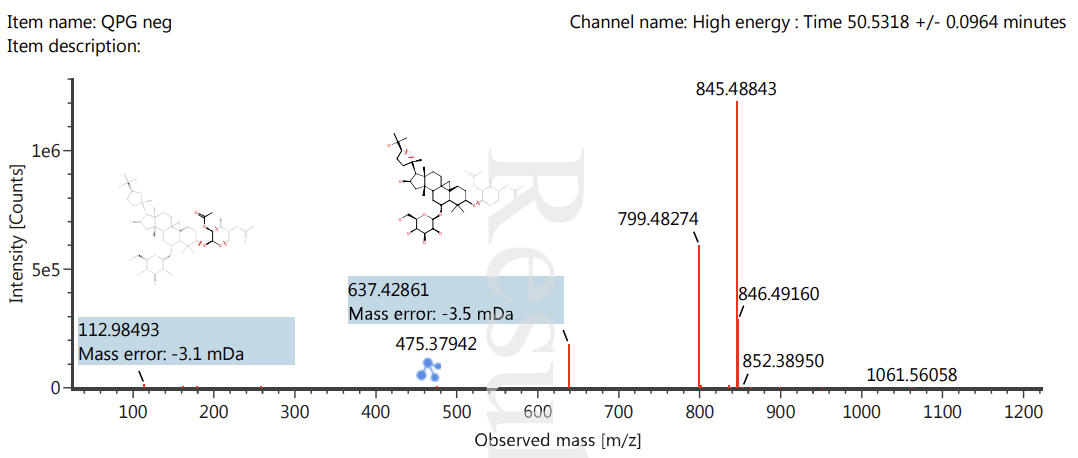


Isoastragaloside I

868.48204 868.4857 913.4839 3.7 4 0 60.03 1053 740 +HCOO


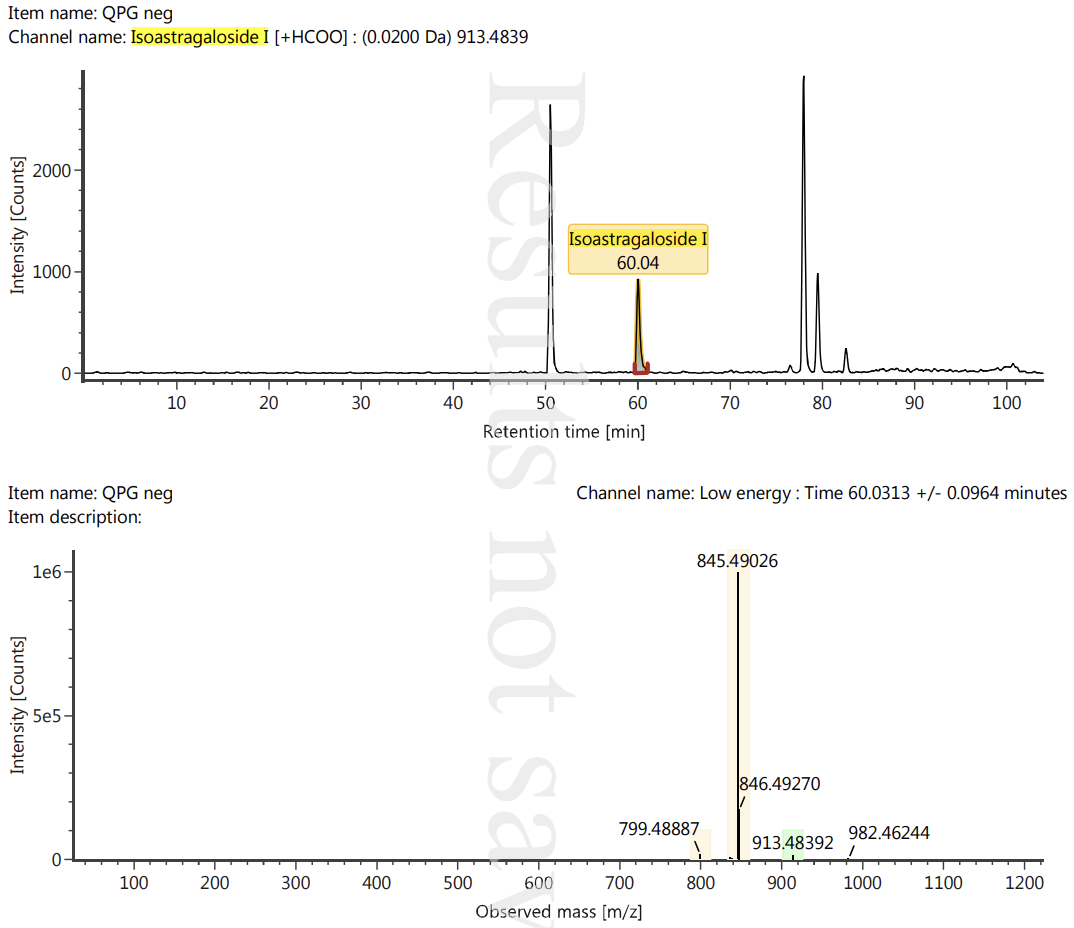


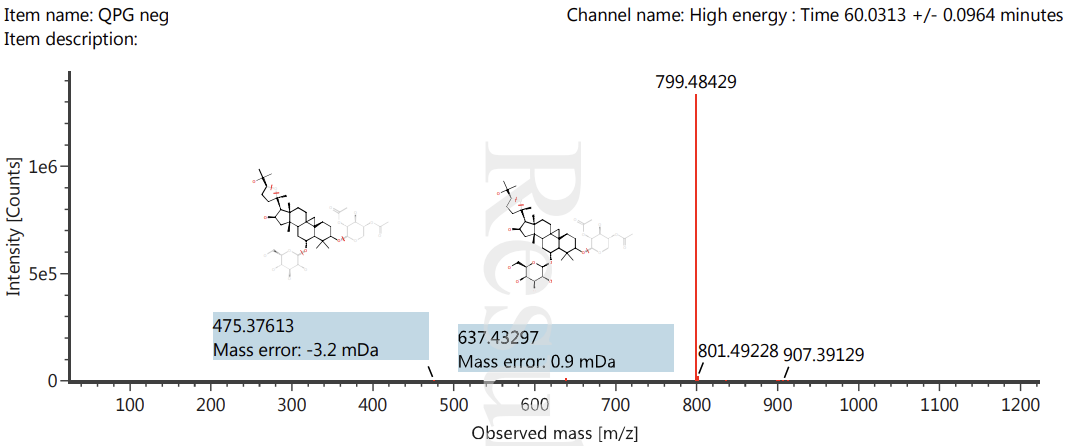


Isoastragaloside I

868.48204 868.4818 913.48 -0.2 -0.2 0 79.49 997 674 +HCOO


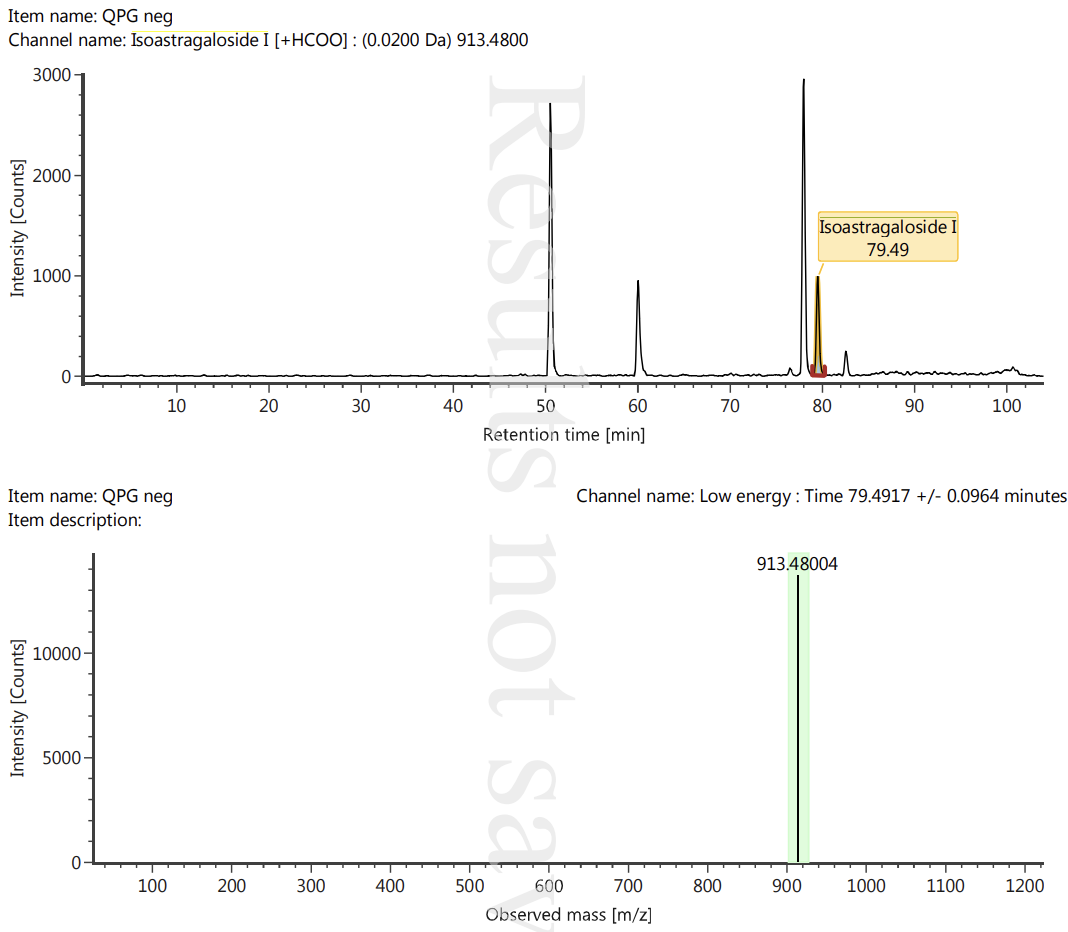


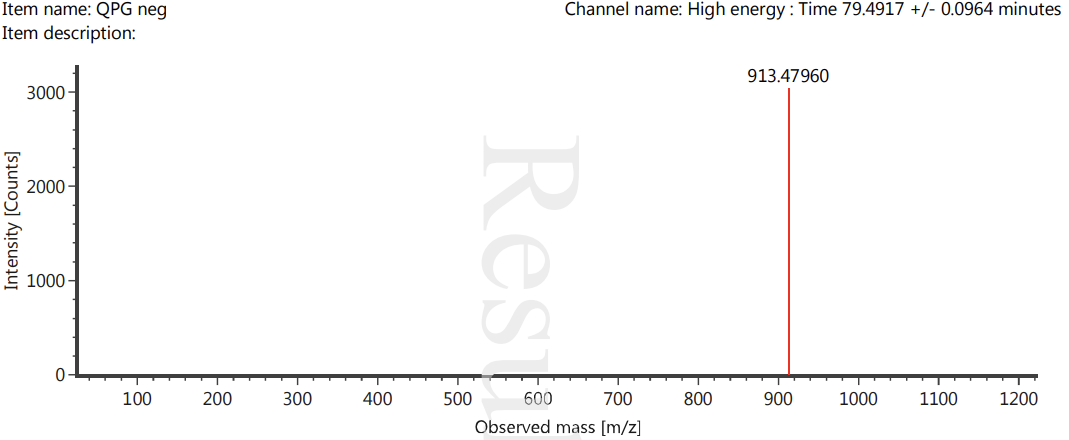


Ginsenoside Rs1

1120.60294 1120.6025 1165.6007 -0.4 -0.4 0 73.61 2679 1583 +HCOO

Ginsenoside Rs1

1120.60294 1120.6048 1119.5976 1.9 1.7 0 71.6 1118 636 -H


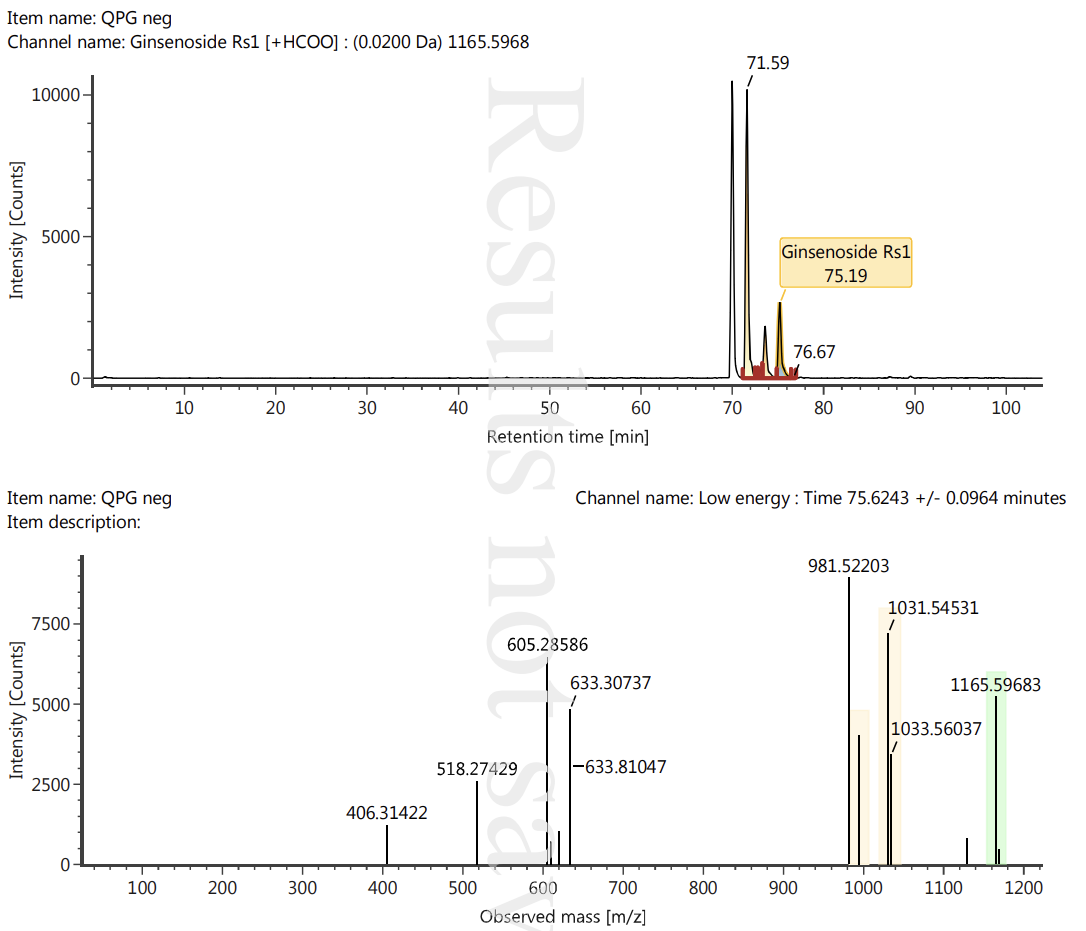


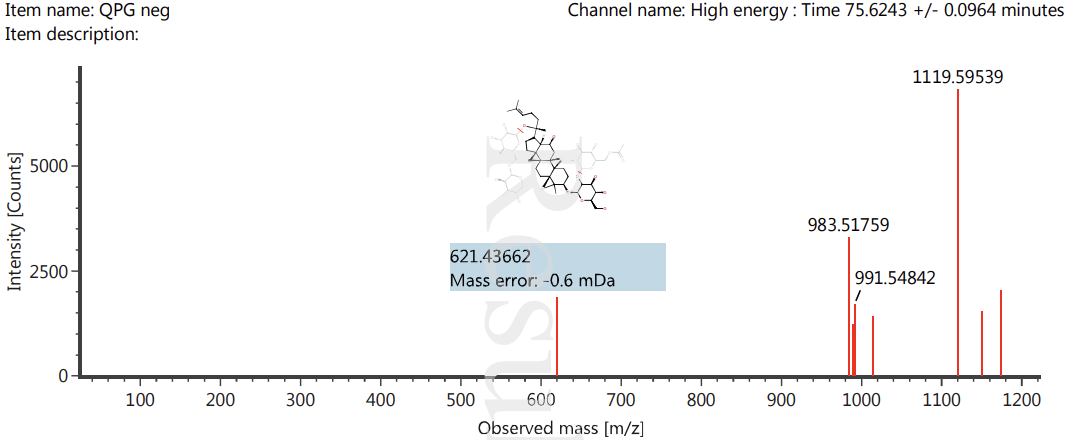


Ginsenoside Rs1

1120.60294 1120.5967 1119.5894 -6.2 -5.6 0 70.01 942 597 -H


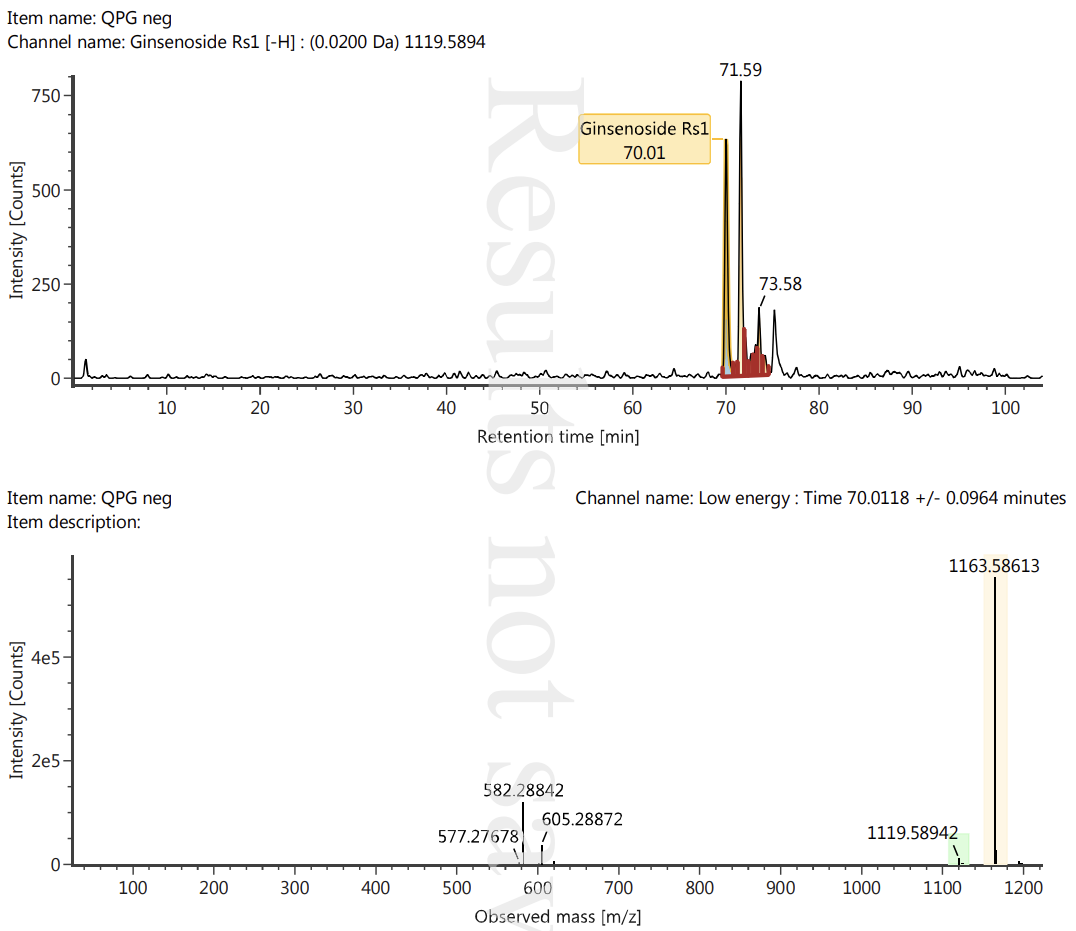


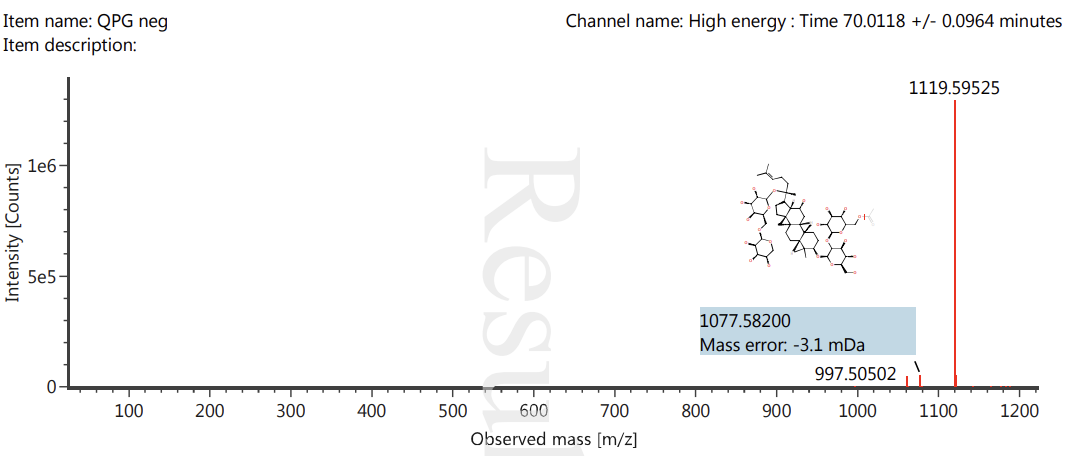


Formononetin

268.07356 268.0761 267.0688 2.5 9.5 0 3.64 1073 1073 -H


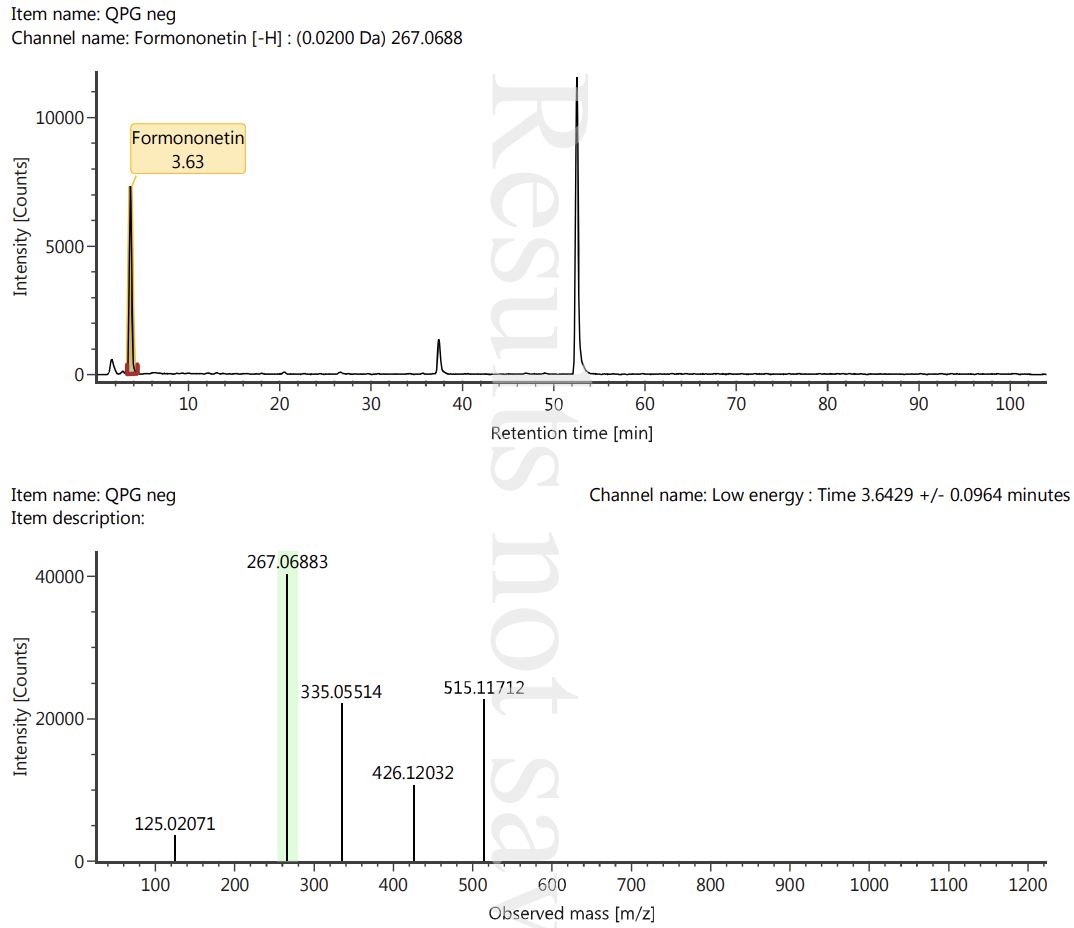


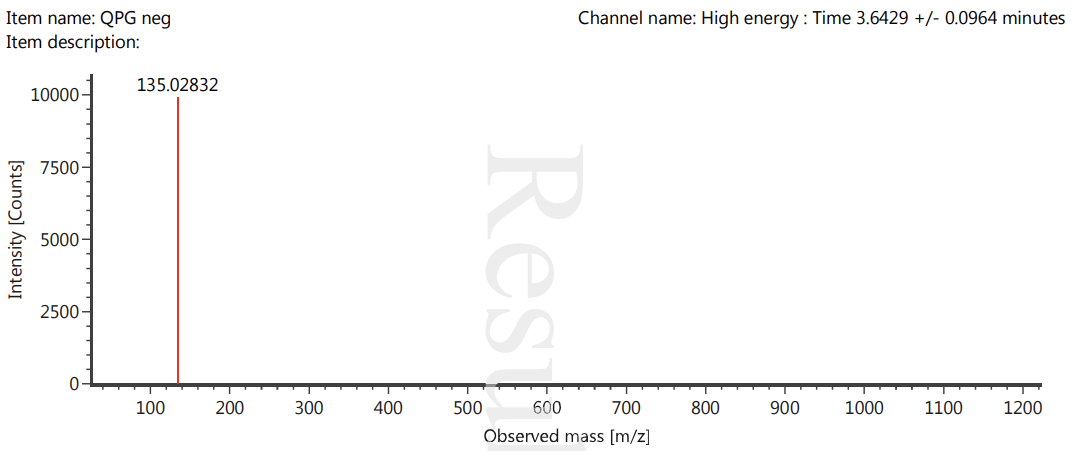


Malonyl-ginsenoside Rb2

1164.59277 1164.5898 1163.5825 -3 -2.6 0 72.08 2492 1024 -H


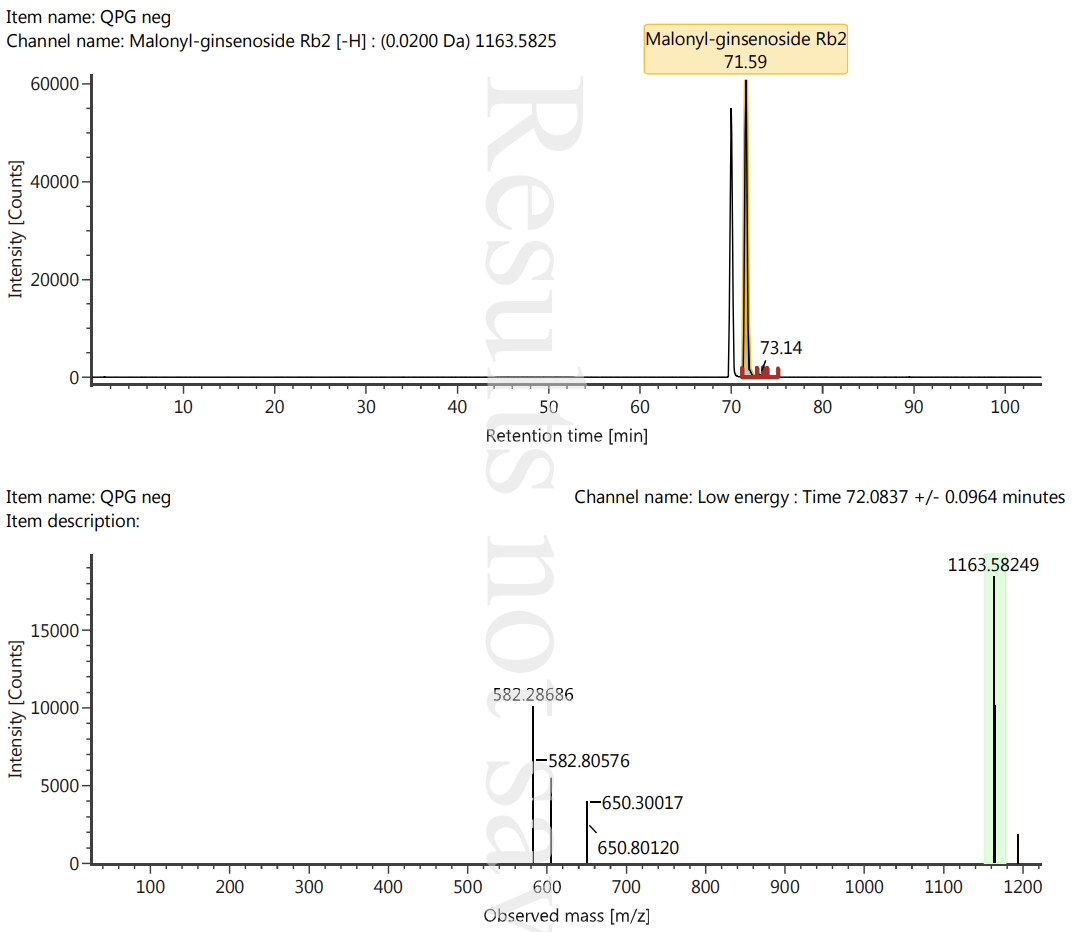


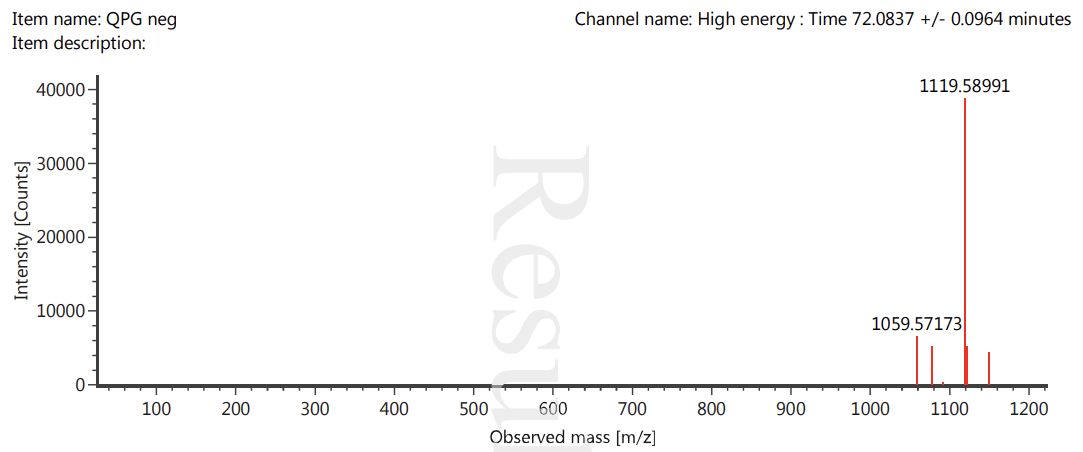


Malonyl-ginsenoside Rb2

1164.59277 1164.5885 1163.5812 -4.3 -3.7 0 73.15 1203 522 -H


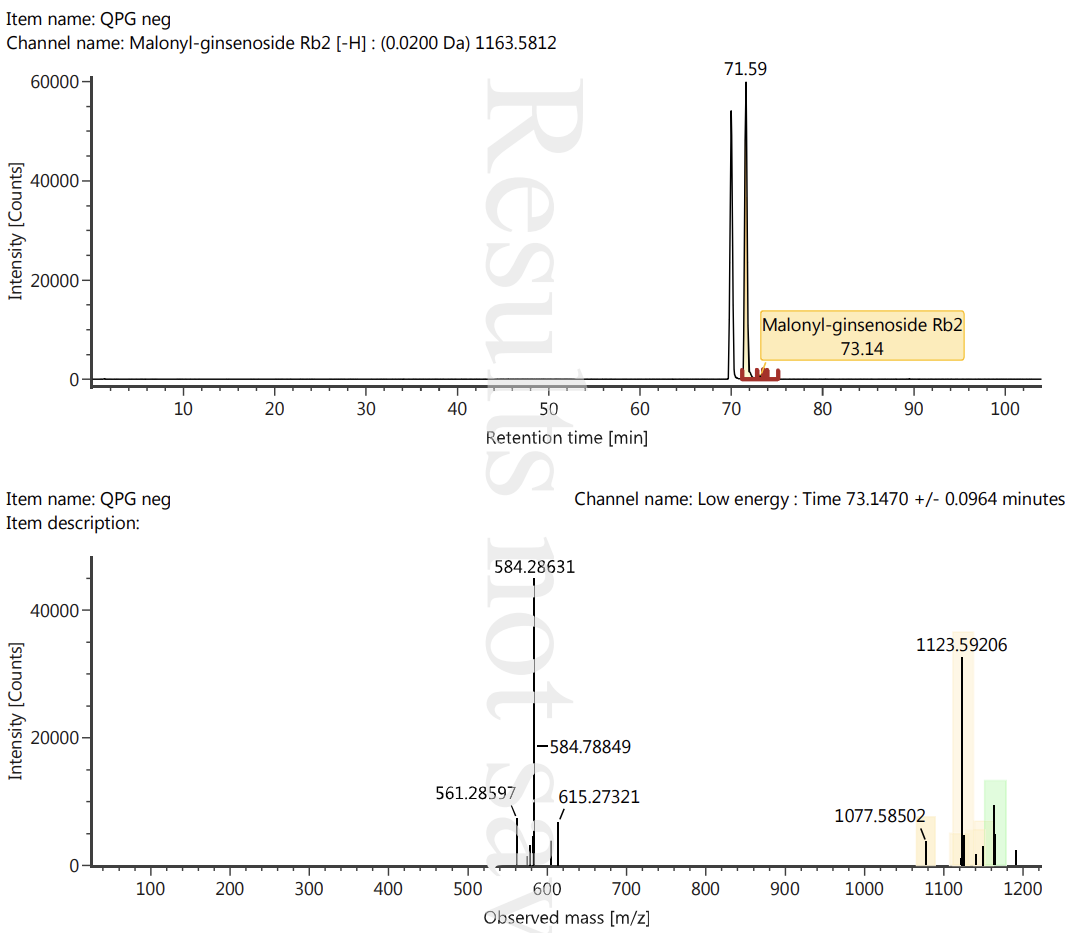


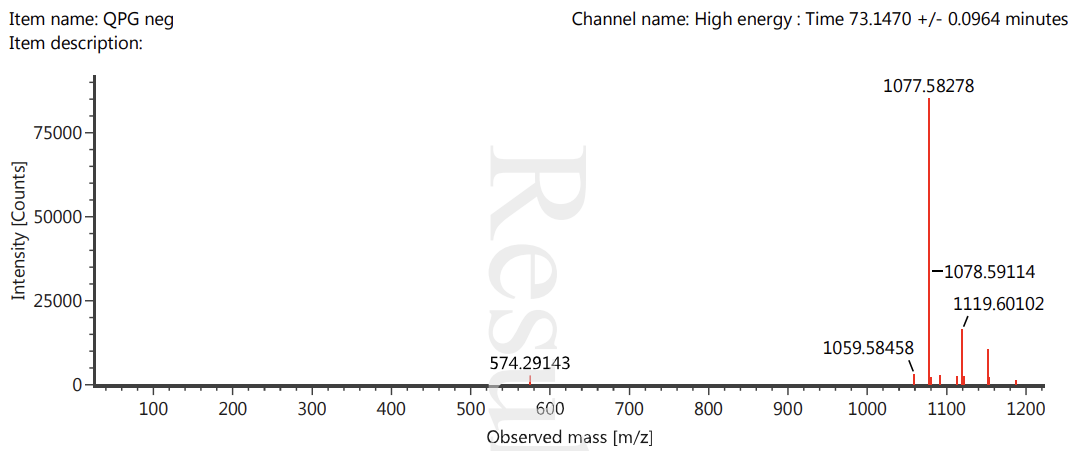


Methyl palmitate

270.25588 270.2538 315.252 -2.1 -6.7 0 76.98 909 808 +HCOO


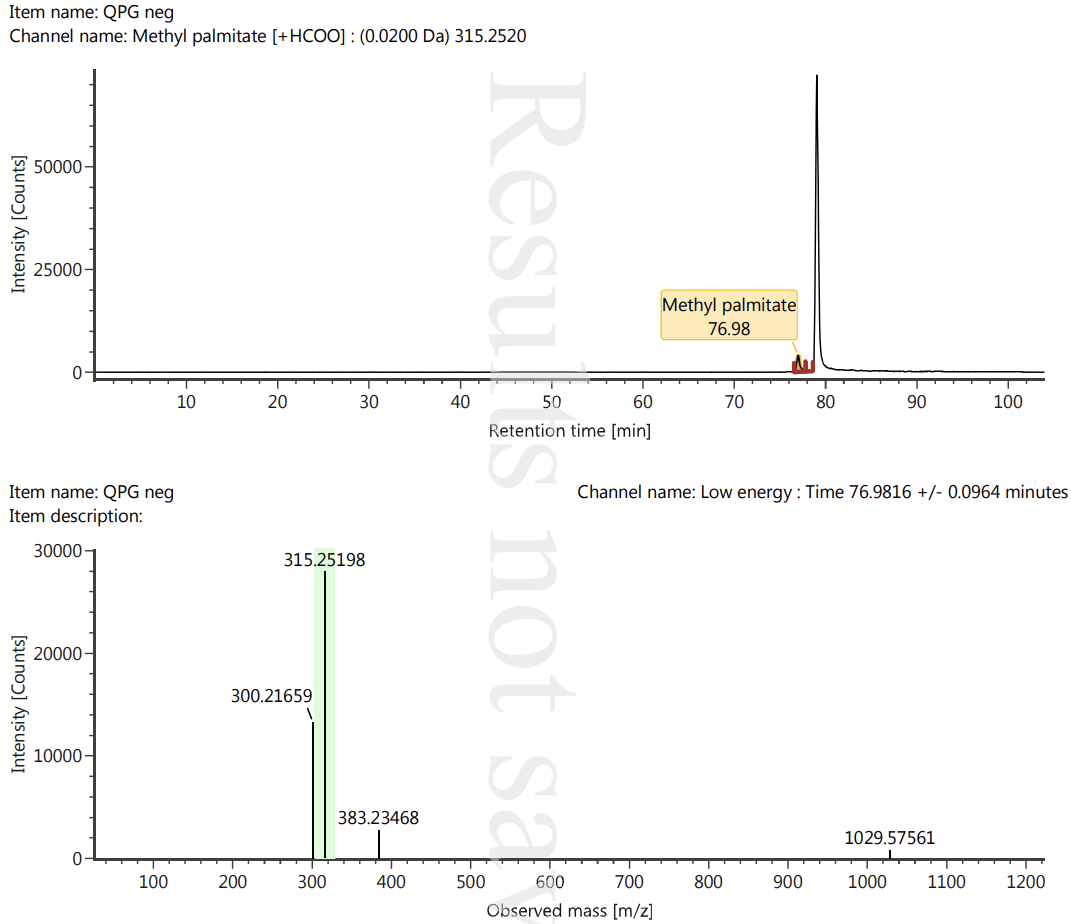


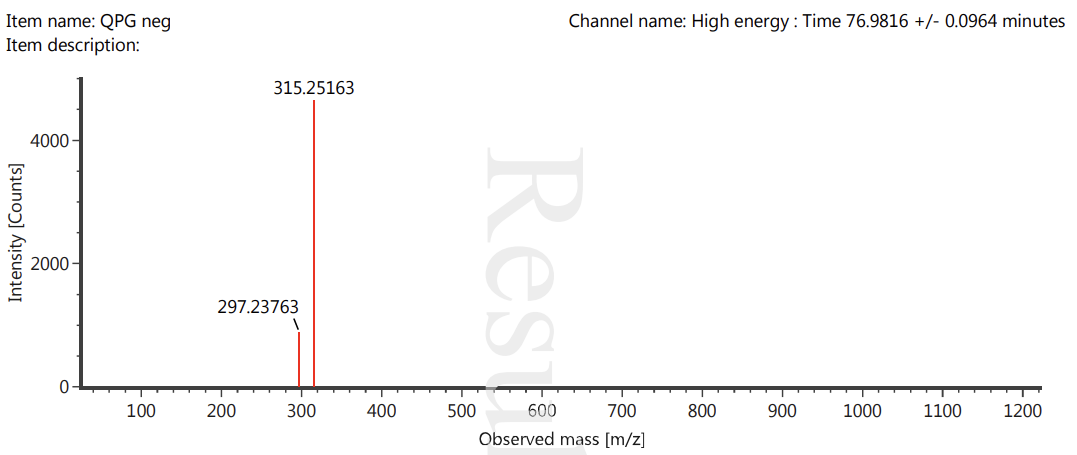


Gypenoside-V

1104.60802 1104.6112 1149.6094 3.2 2.8 0 69.46 1271 714 +HCOO


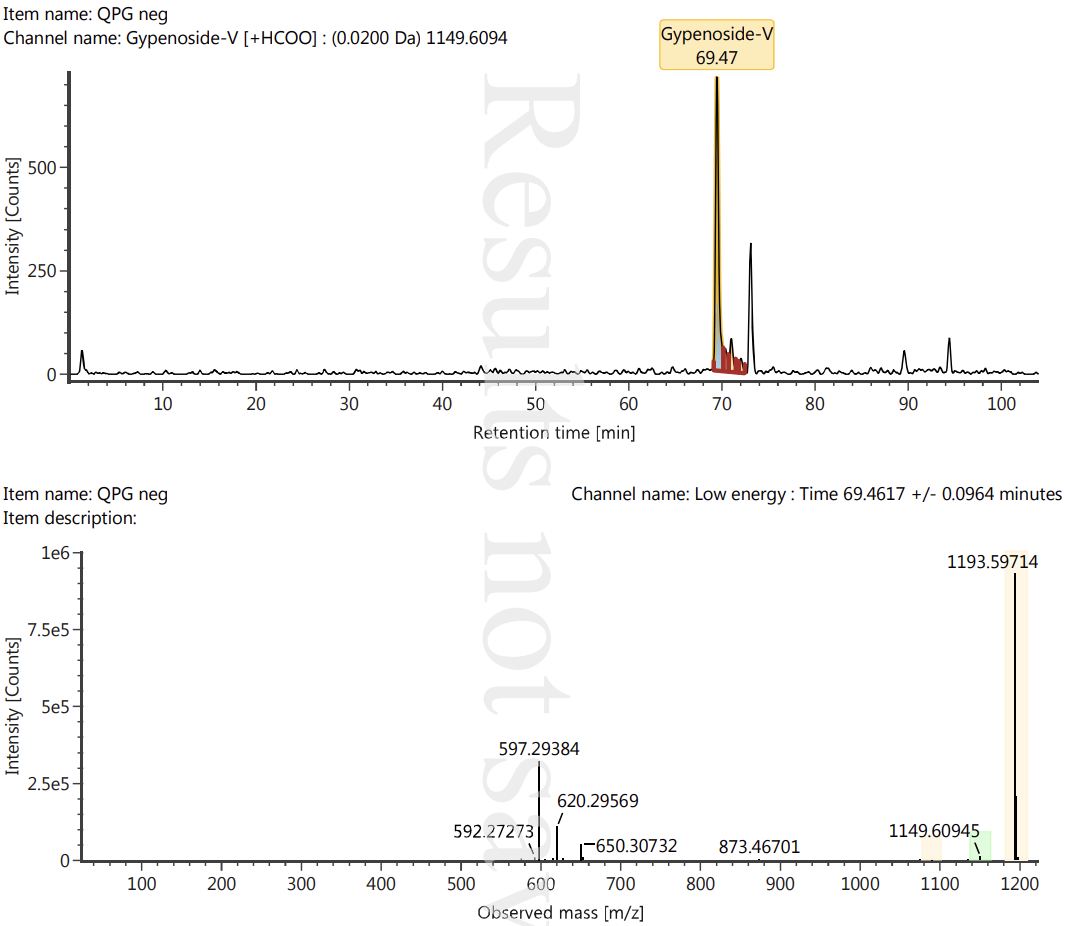


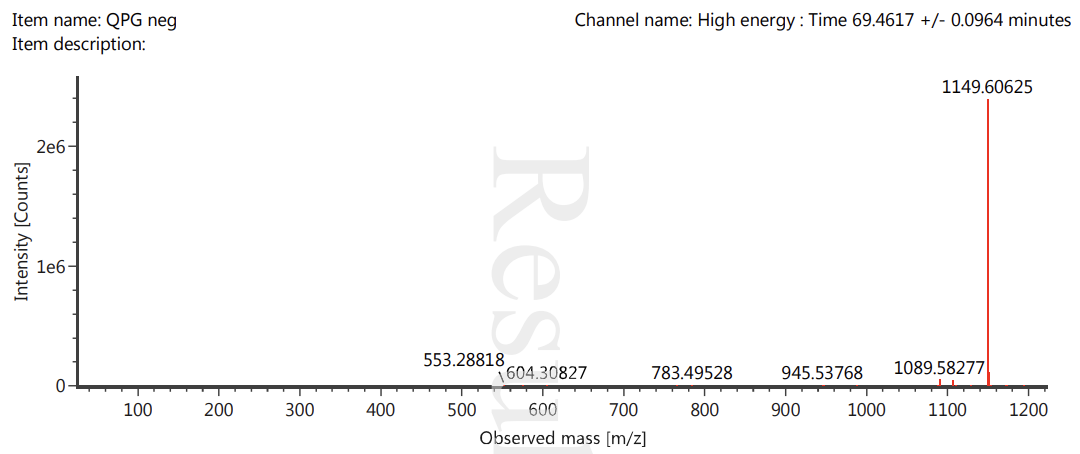


Gypenoside-V

1104.60802 1104.6059 1149.6041 -2.1 -1.8 0 73.05 435 268 +HCOO


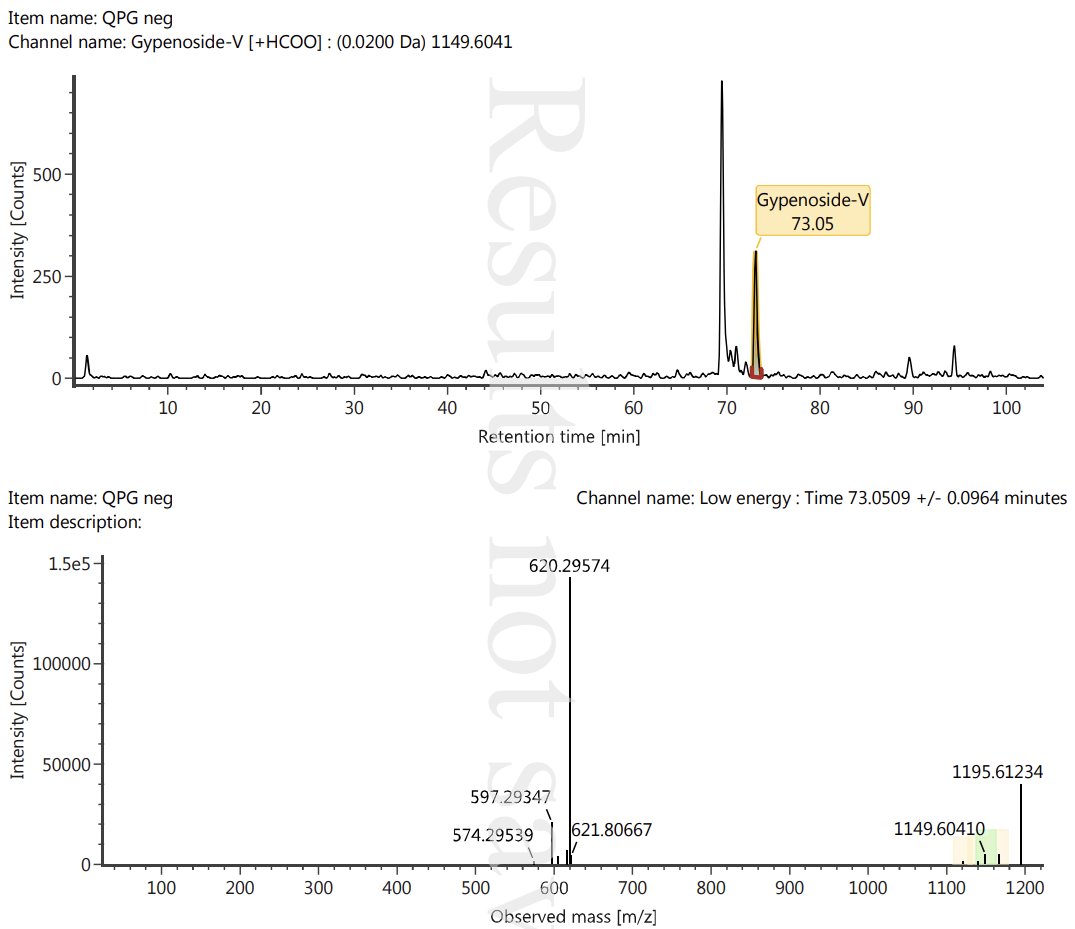


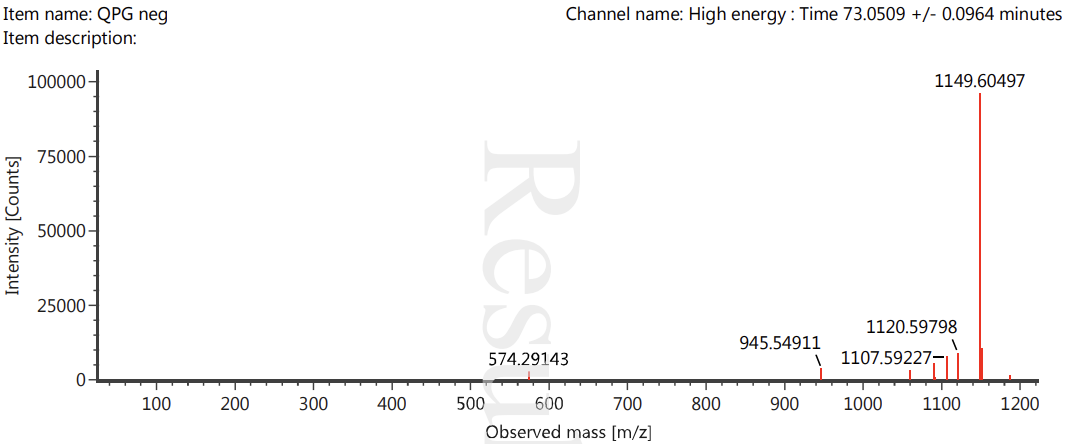


Notoginsenoside Rt

842.50277 842.5019 887.5001 -0.9 -1 0 55.81 803 576 +HCOO


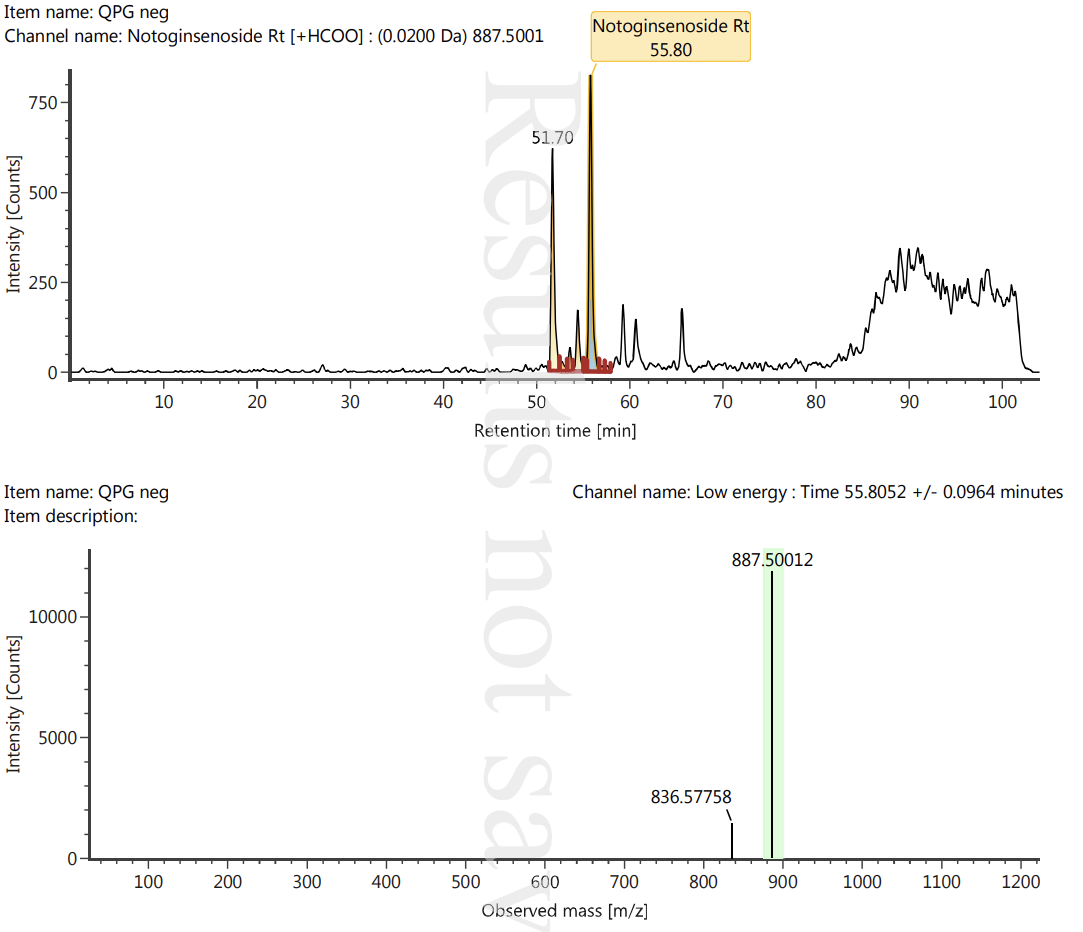

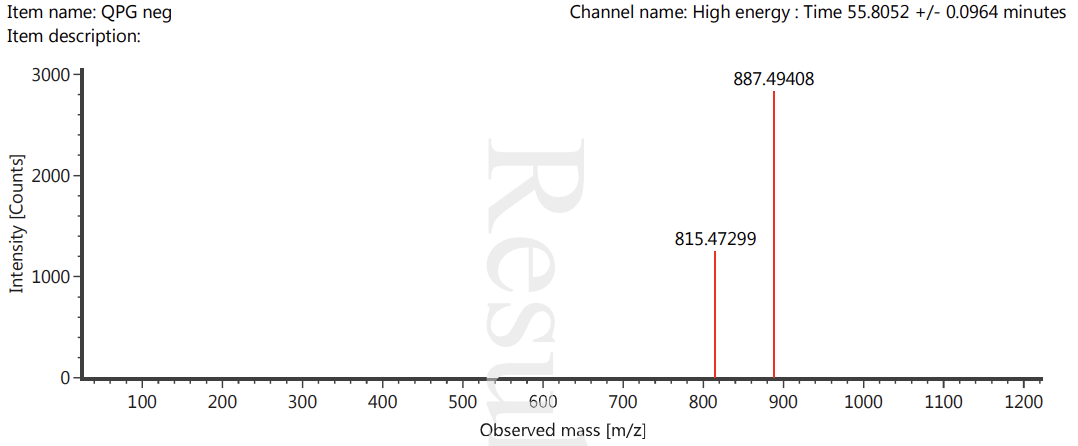


Notoginsenoside Rt

842.50277 842.5083 887.5065 5.5 6.2 0 59.29 112 112 +HCOO


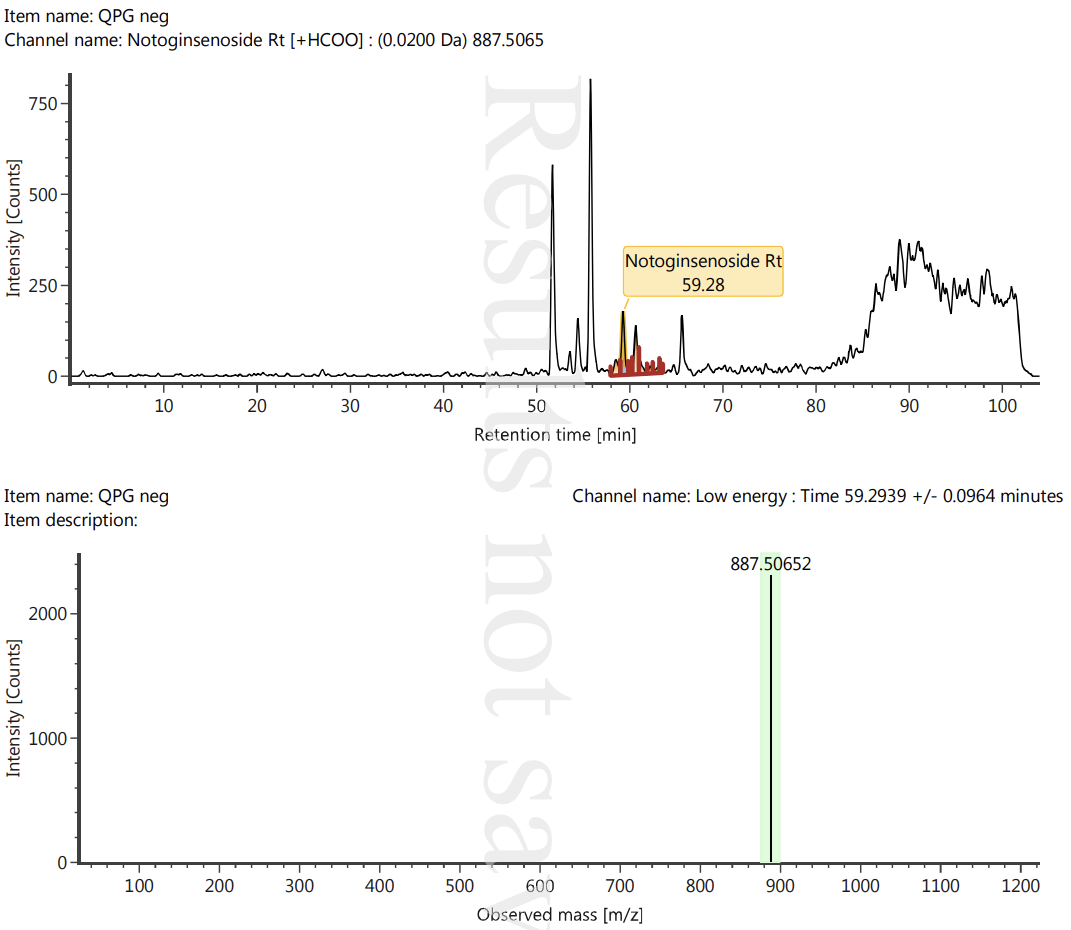

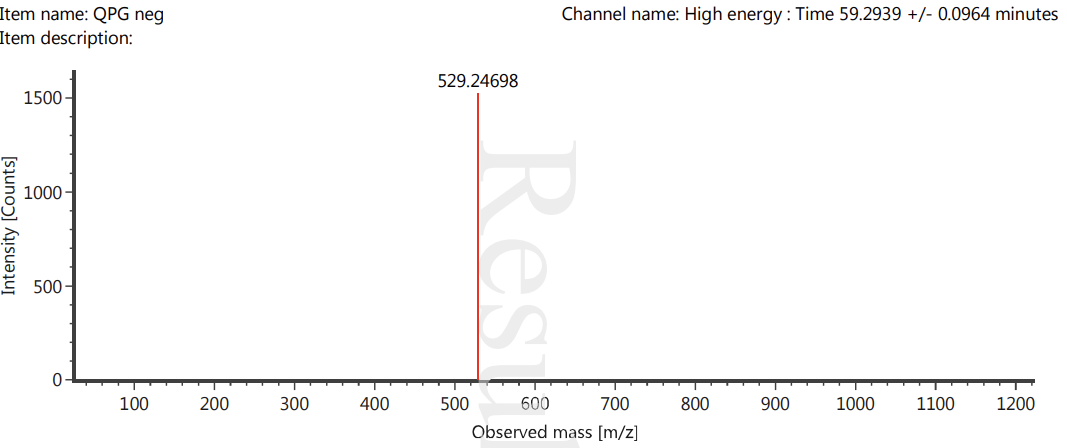


Notoginsenoside Rt

842.50277 842.4975 887.4957 -5.3 -5.9 0 65.6 98 98 +HCOO


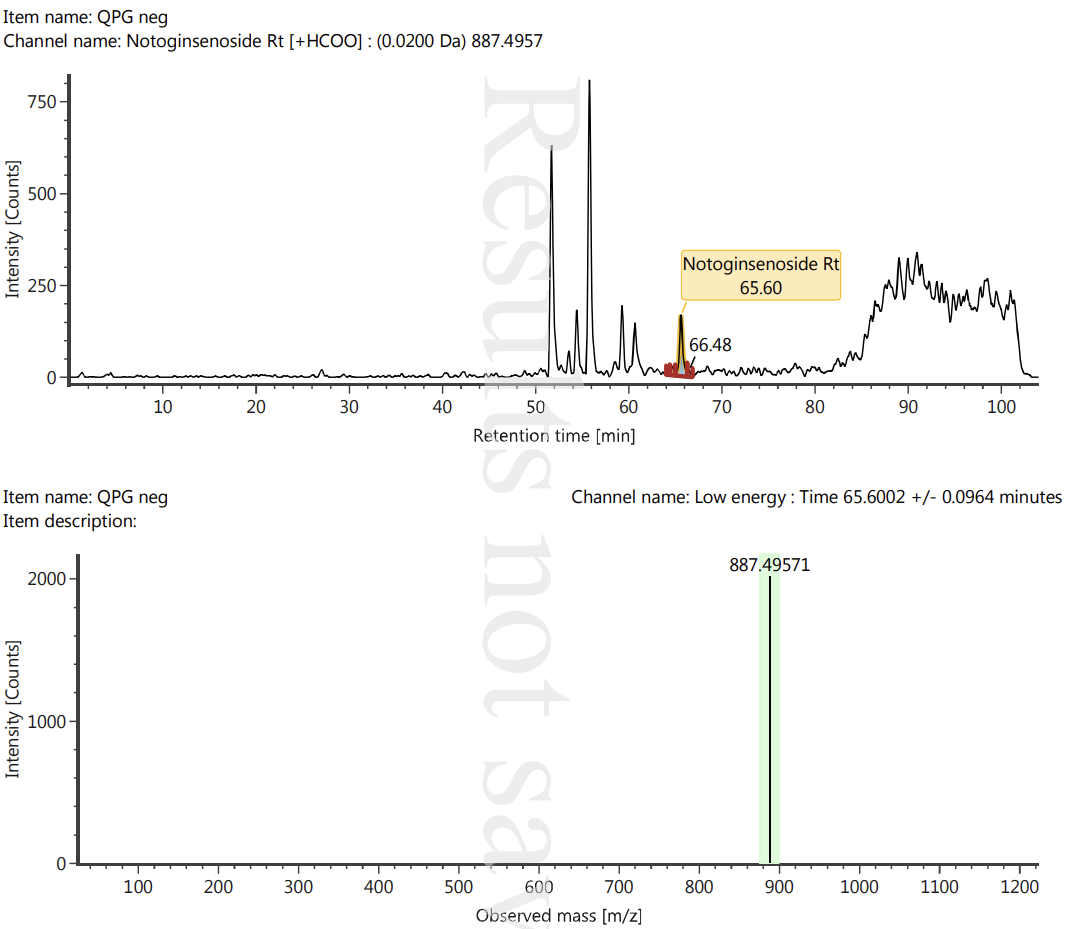


CAFTARIC ACID

312.04813 312.0471 311.0399 -1 -3.2 0 26.59 20903 395 -H


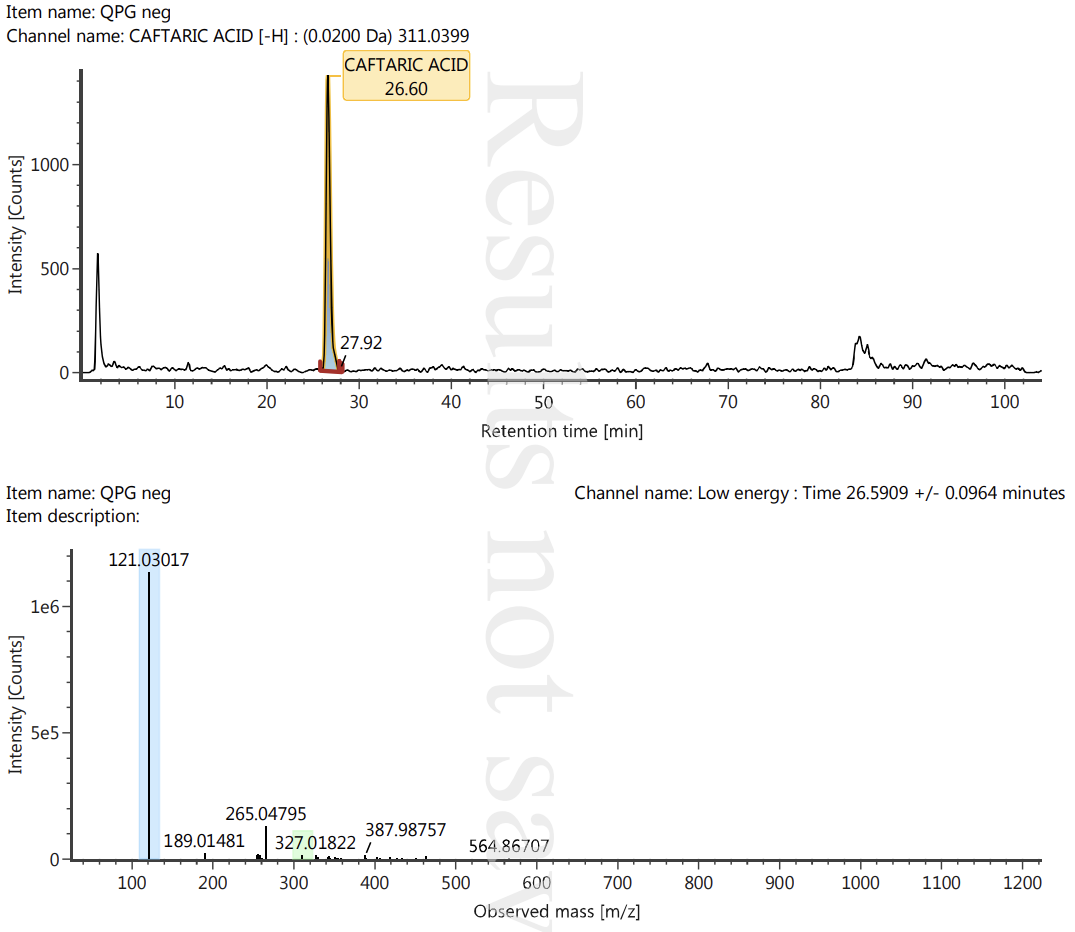


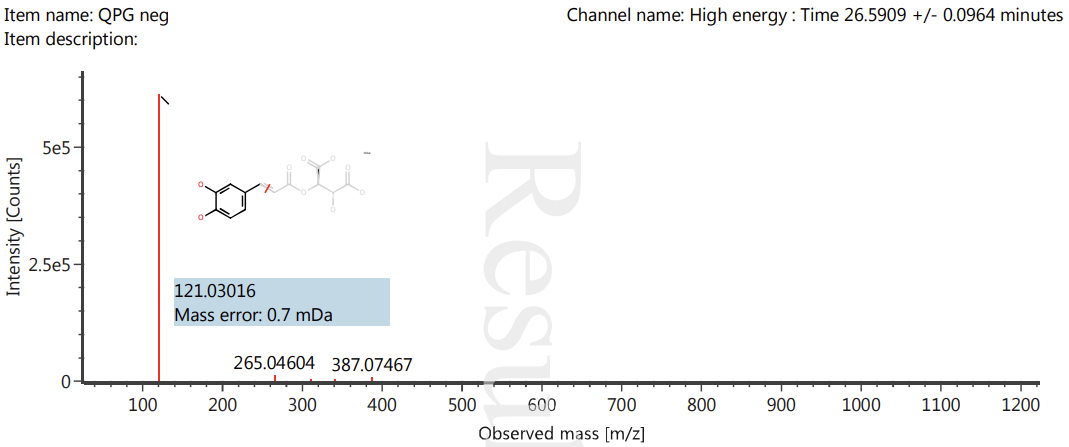


Astraisoflavan-7--O--β-D-glucoside

464.16825 464.1646 463.1573 -3.6 -7.9 0 42.83 726 362 -H, +HCOO


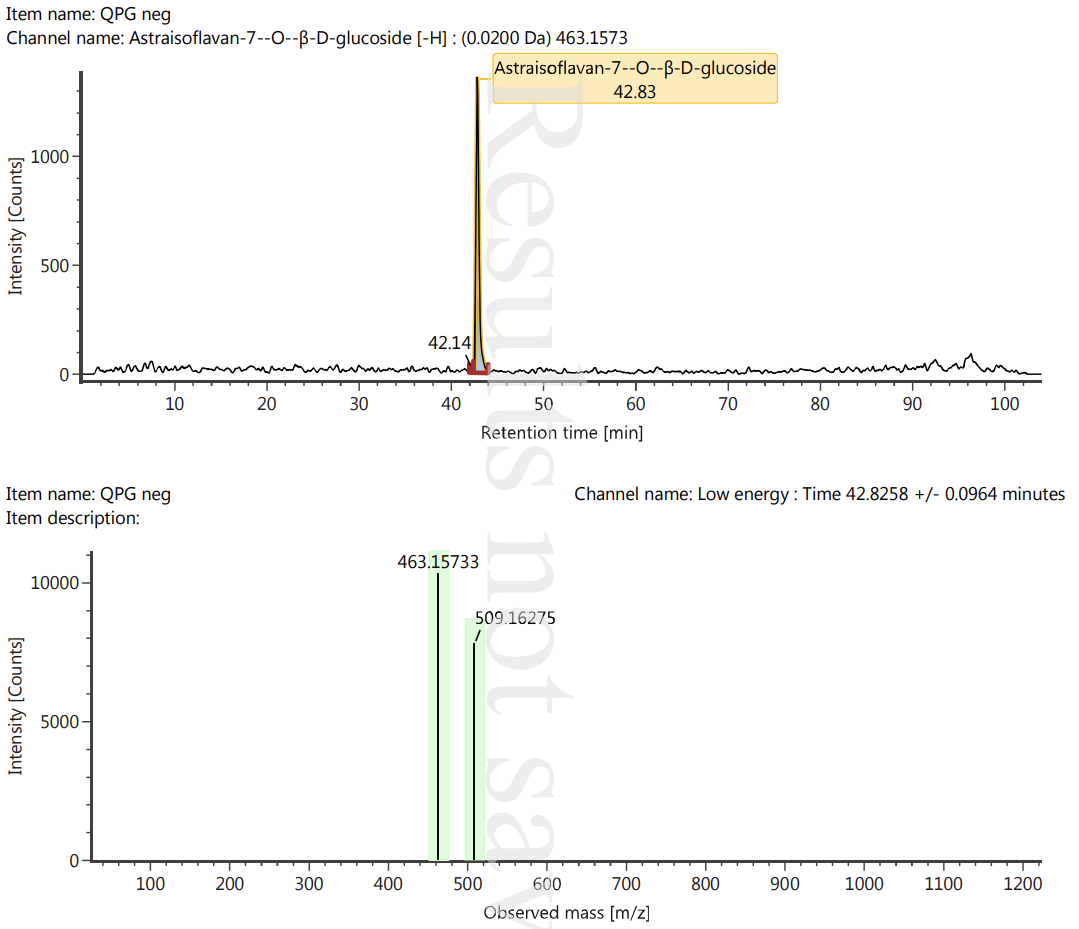


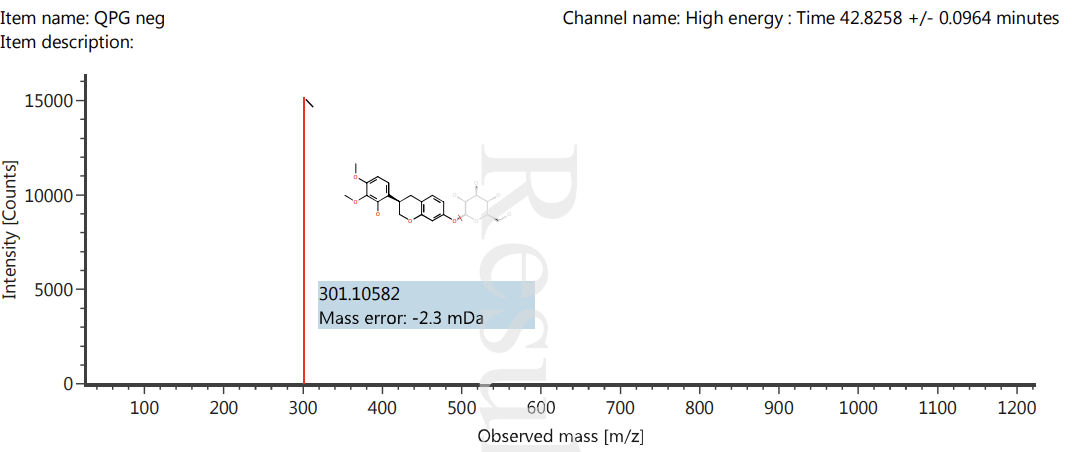


Protocatechualdehyde

138.03169 138.0313 137.024 -0.4 -2.8 0 27.17 358 358 -H


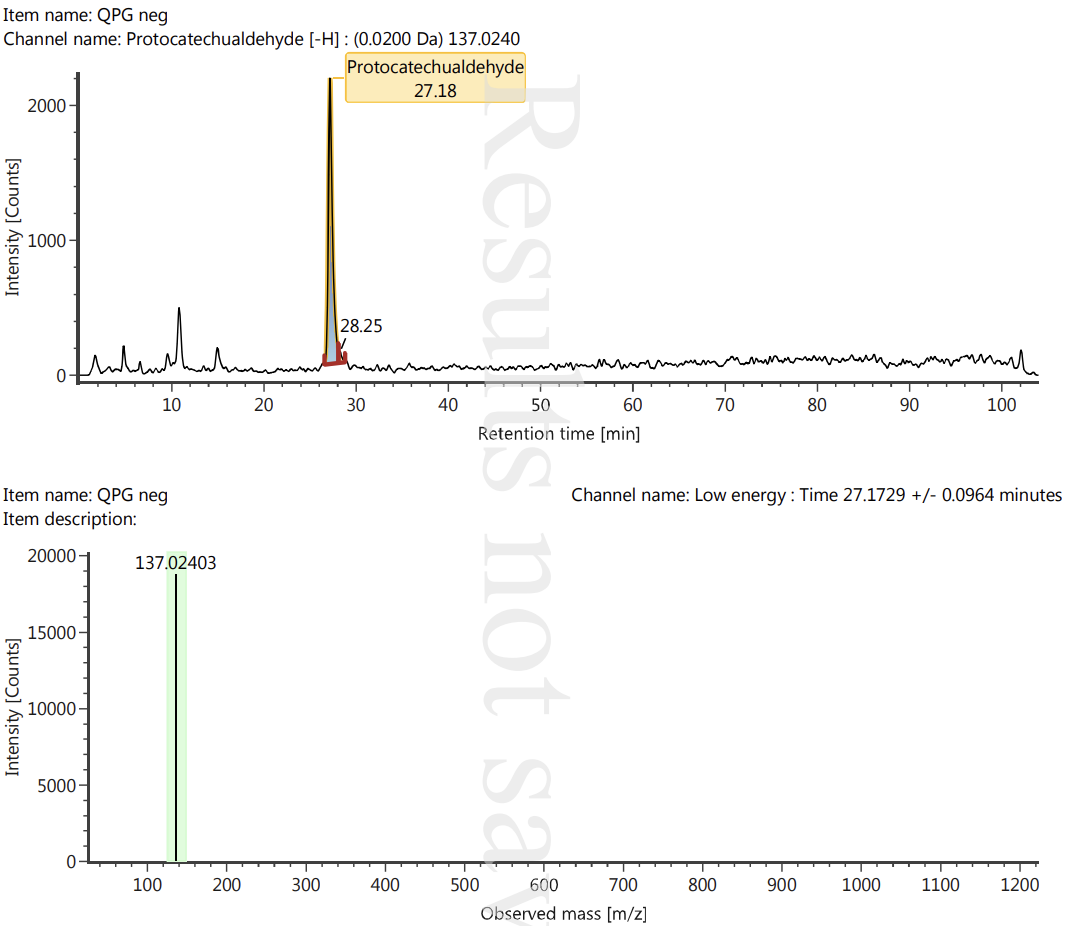


20(S)-ginsenoside Rh1

638.43938 638.434 683.4322 -5.4 -7.9 0 62.95 351 249 +HCOO


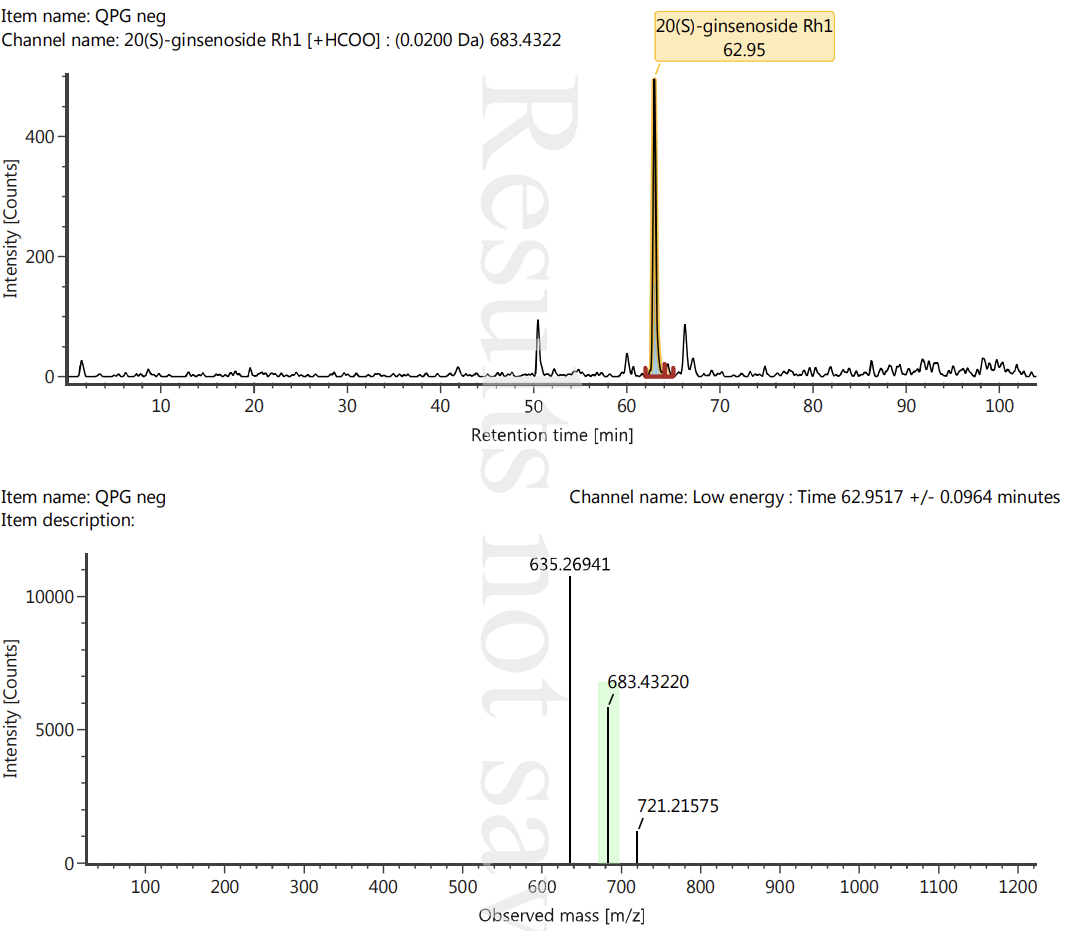


Ginsenoside F2

784.49729 784.5043 829.5025 7 8.4 0 83.95 169 169 +HCOO


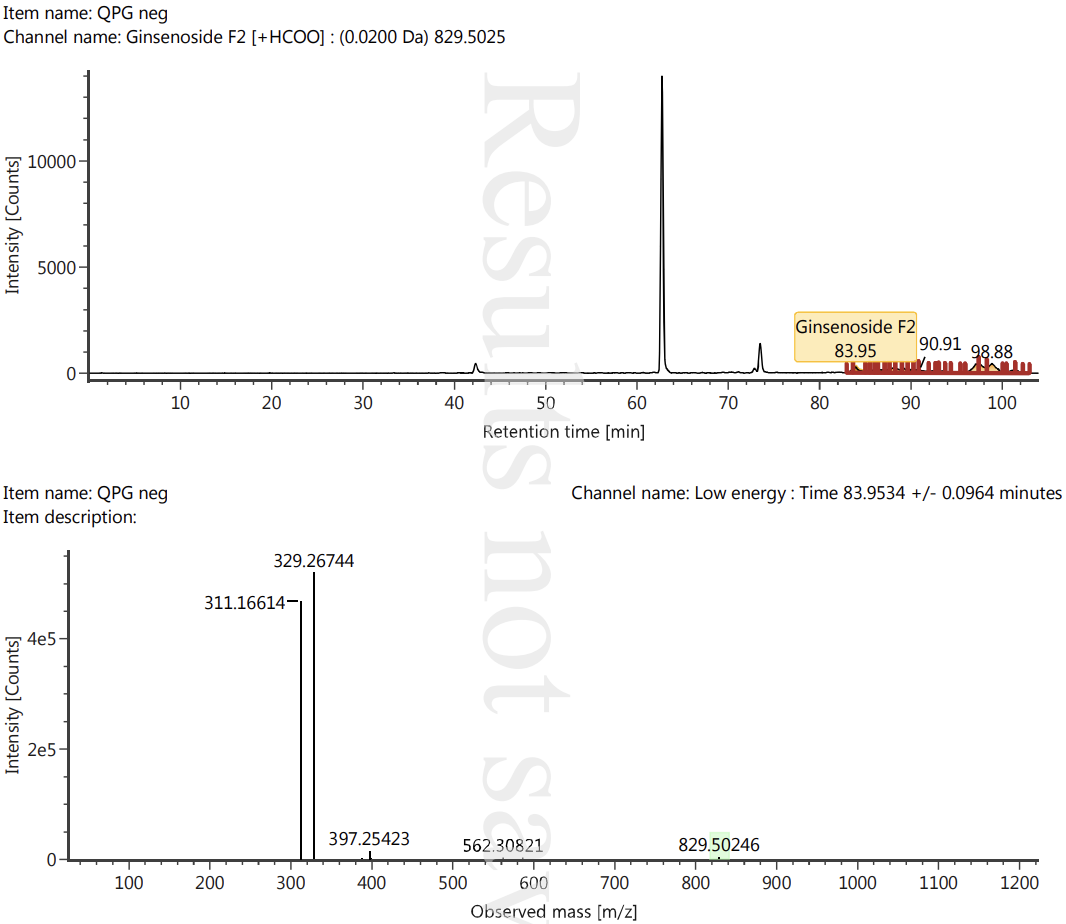


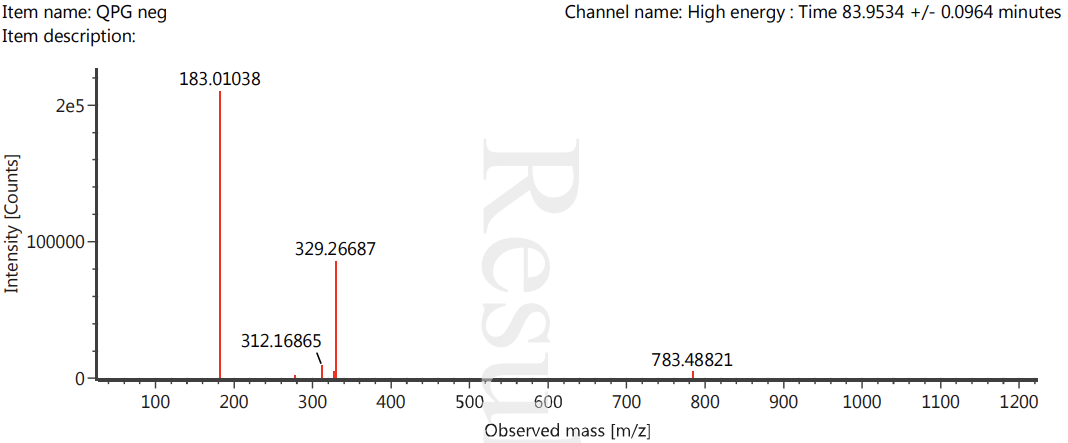


Floralginsenoside Ta

652.41865 652.4248 697.423 6.2 8.9 0 74.26 158 102 +HCOO


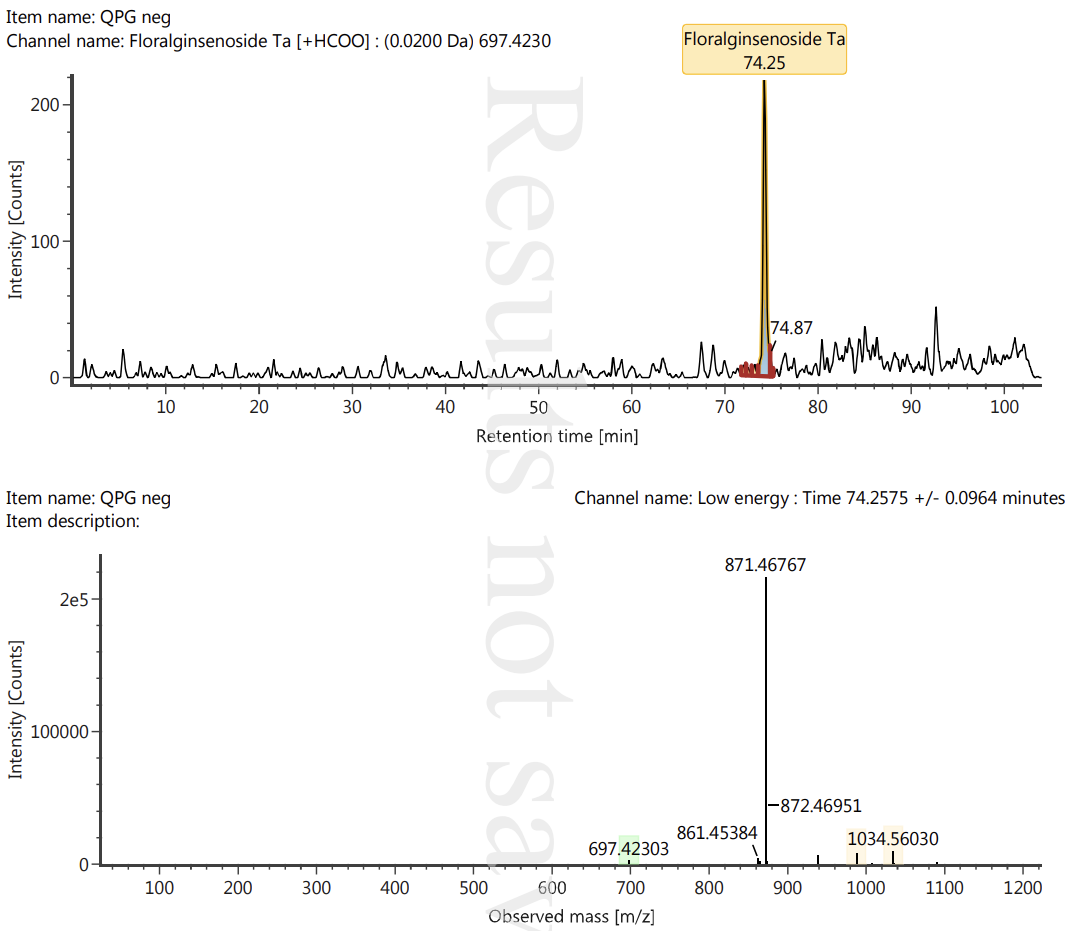

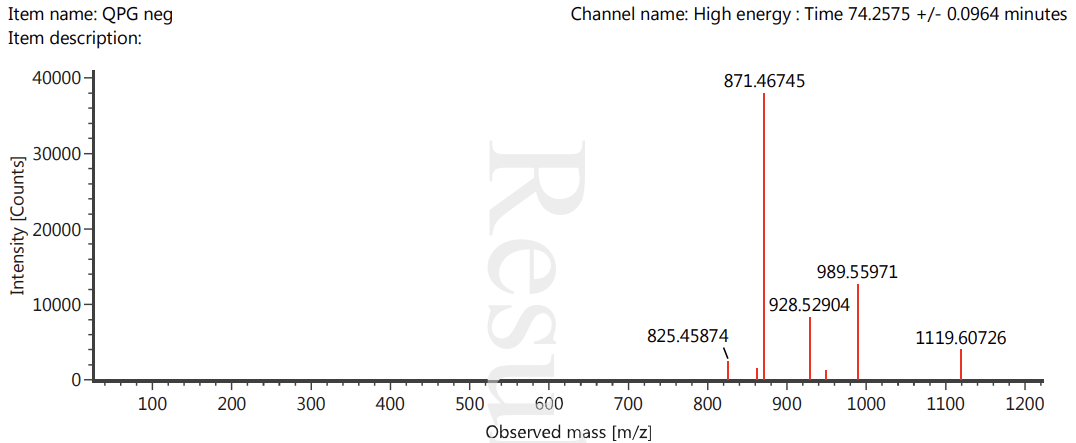


**A105**

给药后0.5小时样本

**Methyl palmitate**

Neutral mass (Da) 270.25588 270.25588

Observed neutral mass (Da) 270.2577 270.2587

Observed m/z 315.2559 315.2569

Mass error (mDa) 1.8 2.8

Mass error (ppm) 5.8 8.9

Expected RT (min) 0 0

Observed RT (min) 76.68 78.75

Detector counts 2251 35633

Response 2034 34504

Adducts +HCOO +HCOO


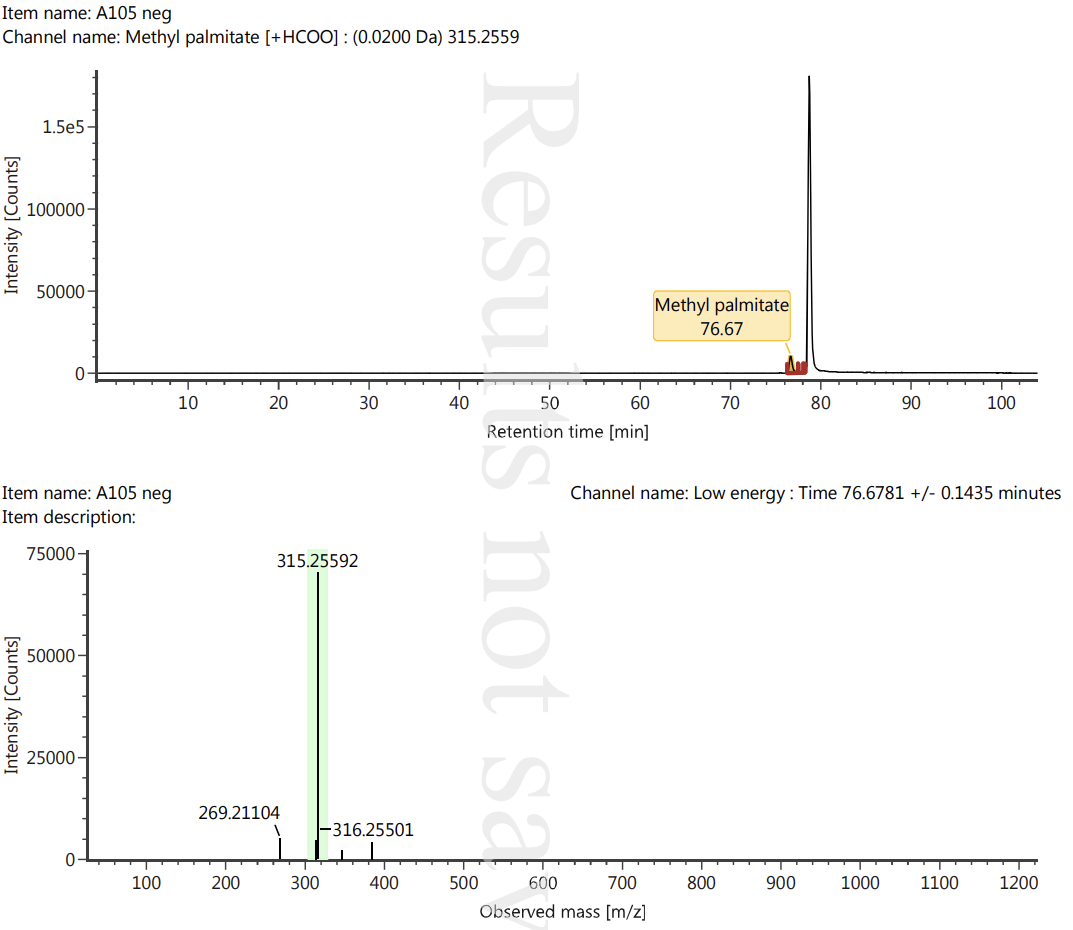

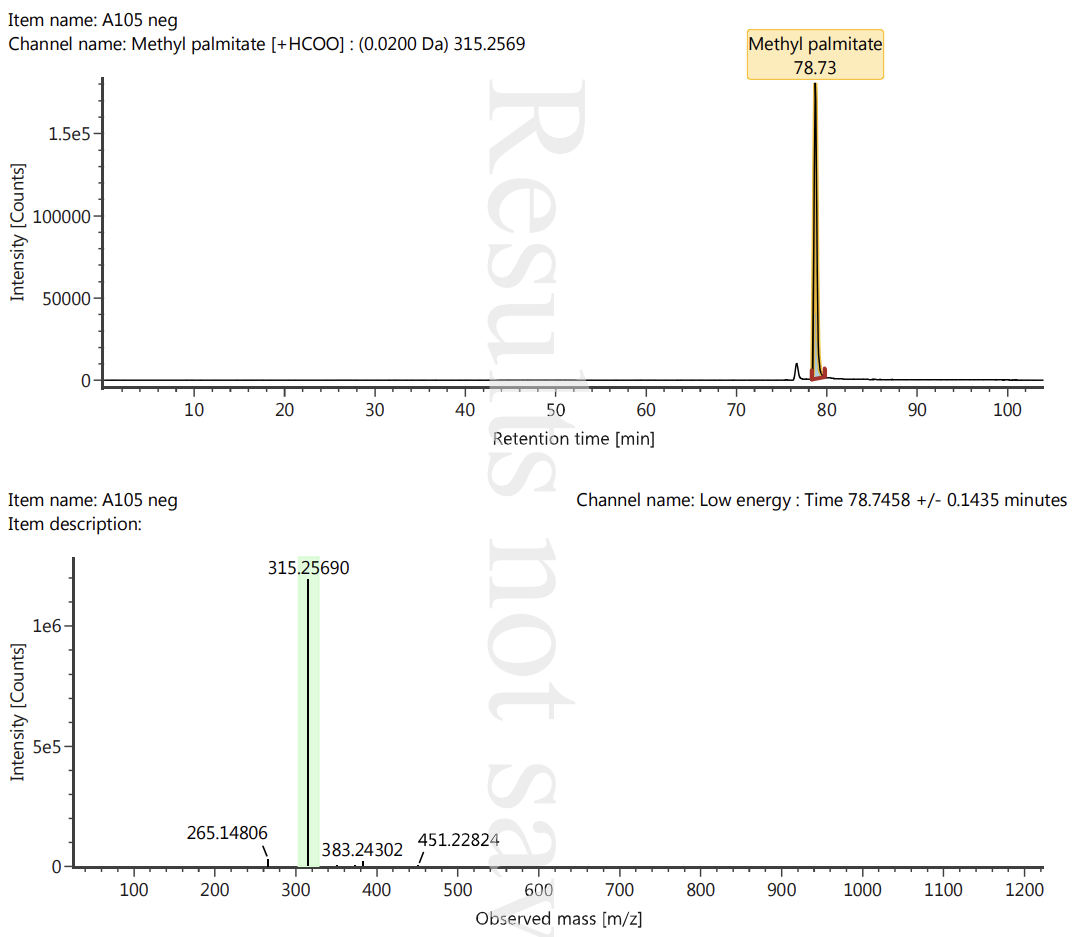


**Formononetin**

Neutral mass (Da) 268.07356

Observed neutral mass (Da) 268.0735

Observed m/z 313.0717

Mass error (mDa) -0.1

Mass error (ppm) -0.3

Expected RT (min) 0

Observed RT (min) 99.58

Detector counts 132

Response 132

Adducts +HCOO


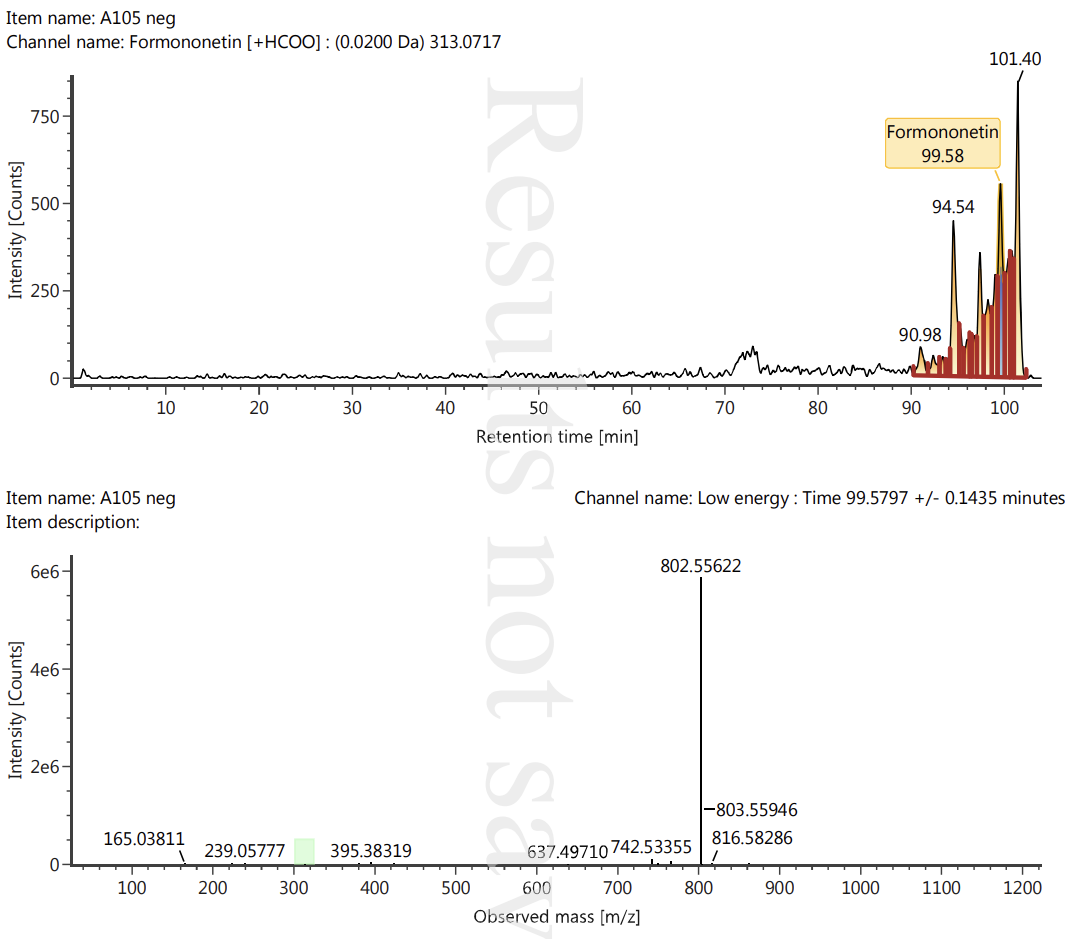


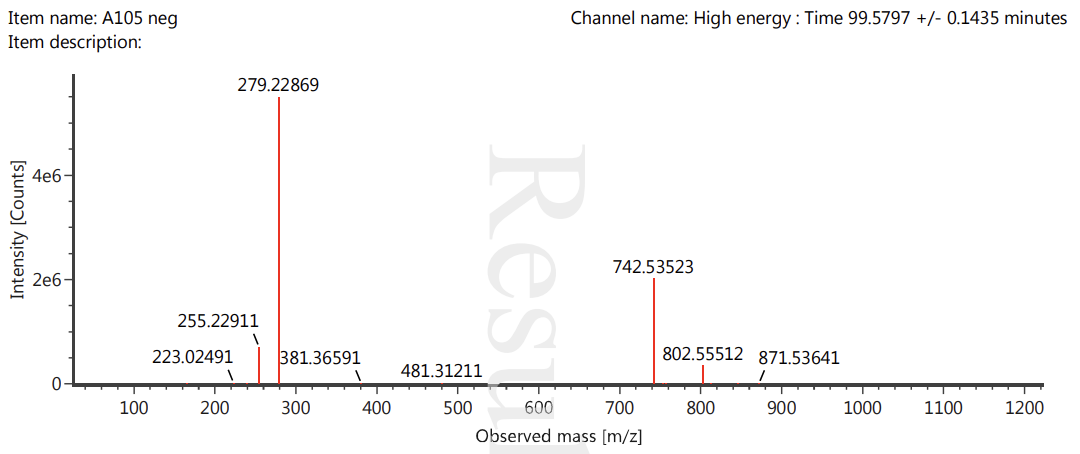


**A31**

给药后1小时样本

**Methyl palmitate**

Neutral mass (Da) 270.25588

Observed neutral mass (Da) 270.2582

Observed m/z 315.2564

Mass error (mDa) 2.3

Mass error (ppm) 7.4

Expected RT (min) 0

Observed RT (min) 76.74

Detector counts 1331

Response 1331

Adducts +HCOO


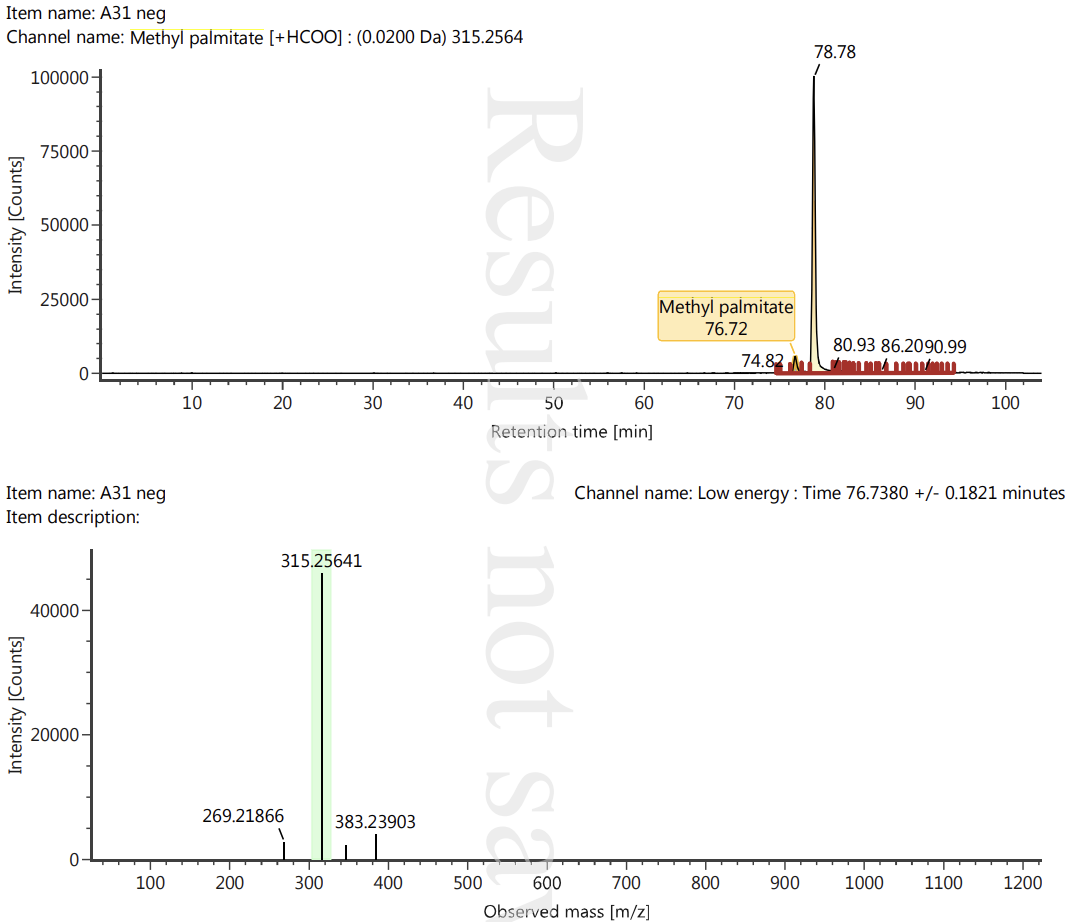


**20(S)-protopanaxadiol**

Neutral mass (Da) 460.39165

Observed neutral mass (Da) 460.3876

Observed m/z 459.3803

Mass error (mDa) -4

Mass error (ppm) -8.8

Expected RT (min) 0

Observed RT (min) 101.68

Detector counts 256

Response 256

Adducts -H


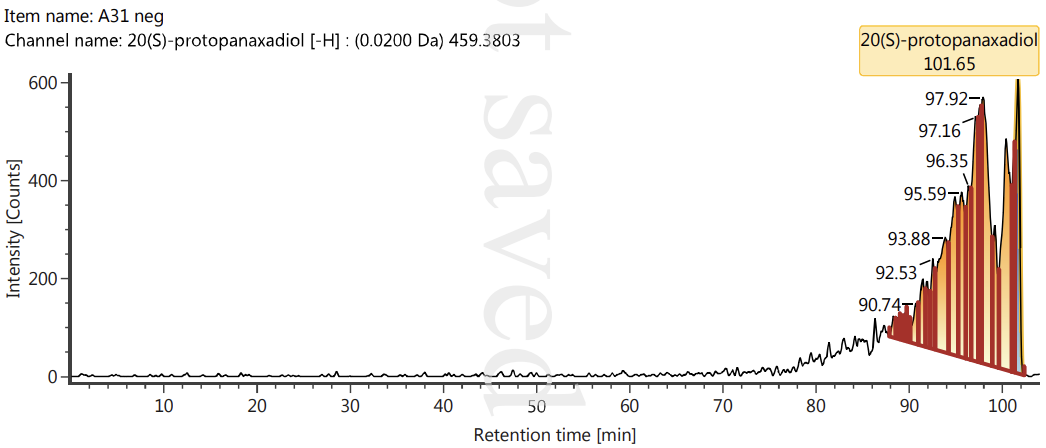


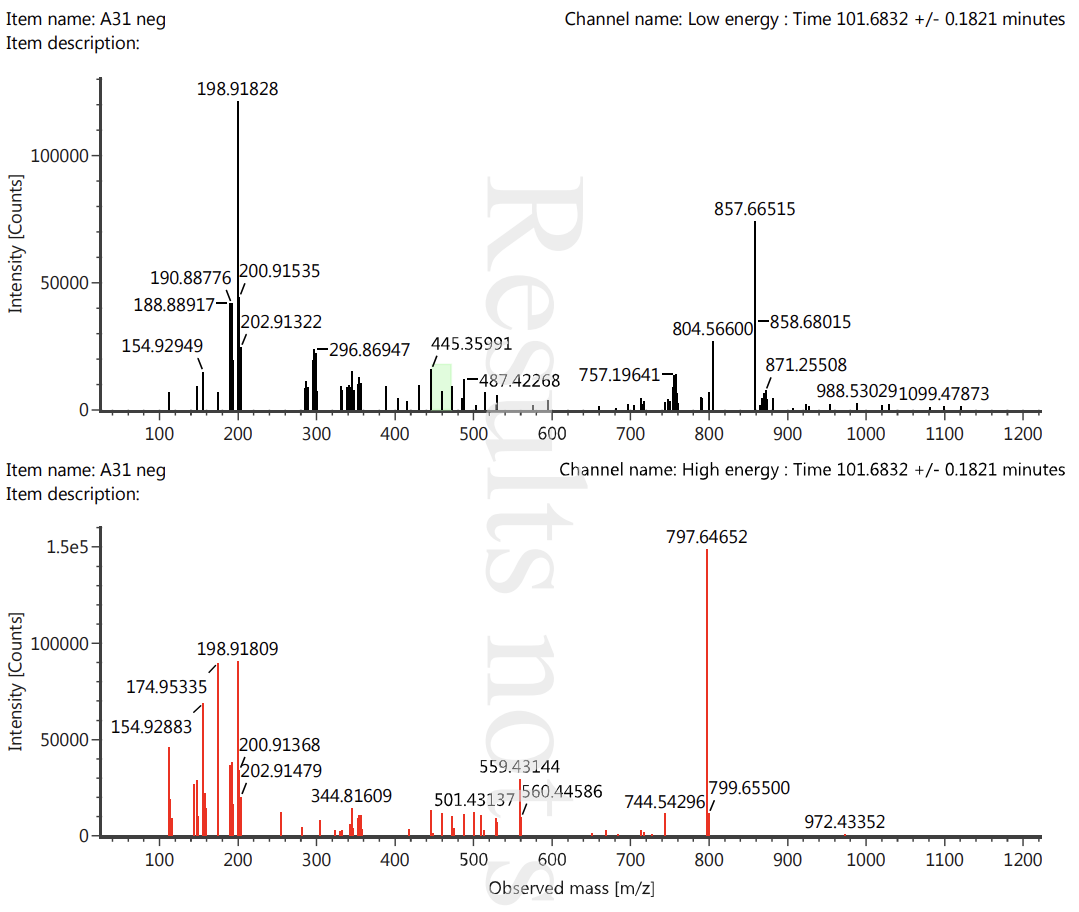


**Palmitic acid**

Neutral mass (Da) 256.24023

Observed neutral mass (Da) 256.2391

Observed m/z 255.2318

Mass error (mDa) -1.2

Mass error (ppm) -4.5

Expected RT (min) 0

Observed RT (min) 89.37

Detector counts 64

Response 64

Adducts -H


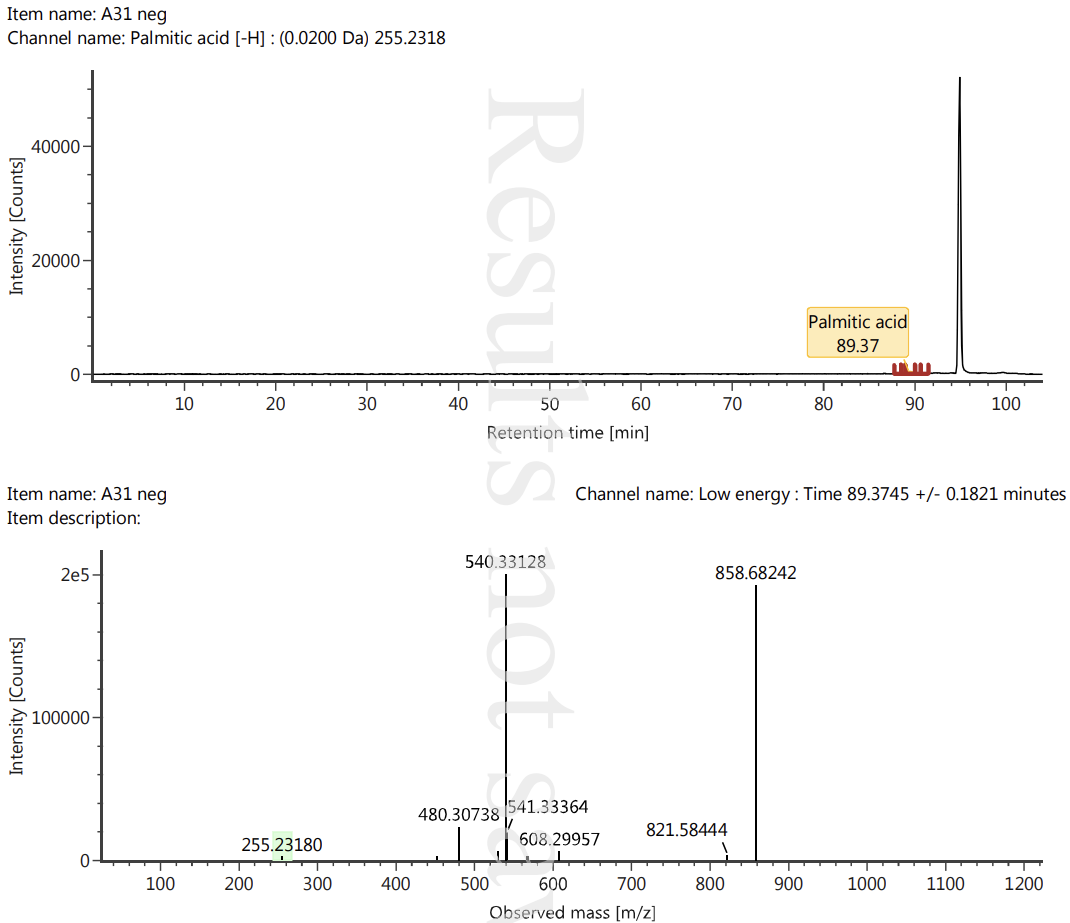


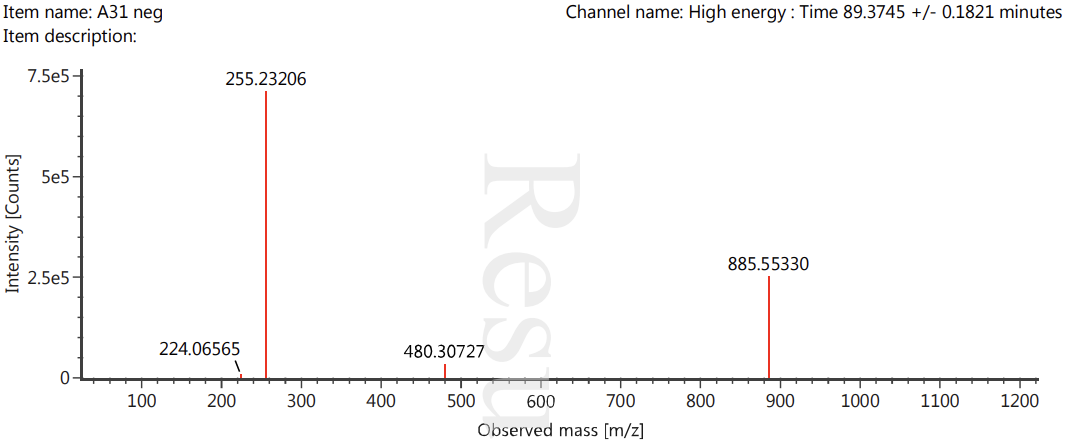


**Linolenic acid**

Neutral mass (Da) 278.22458

Observed neutral mass (Da) 278.2252

Observed m/z 277.2179

Mass error (mDa) 0.6

Mass error (ppm) 2.3

Expected RT (min) 0

Observed RT (min) 90.32

Detector counts 62

Response 62

Adducts -H


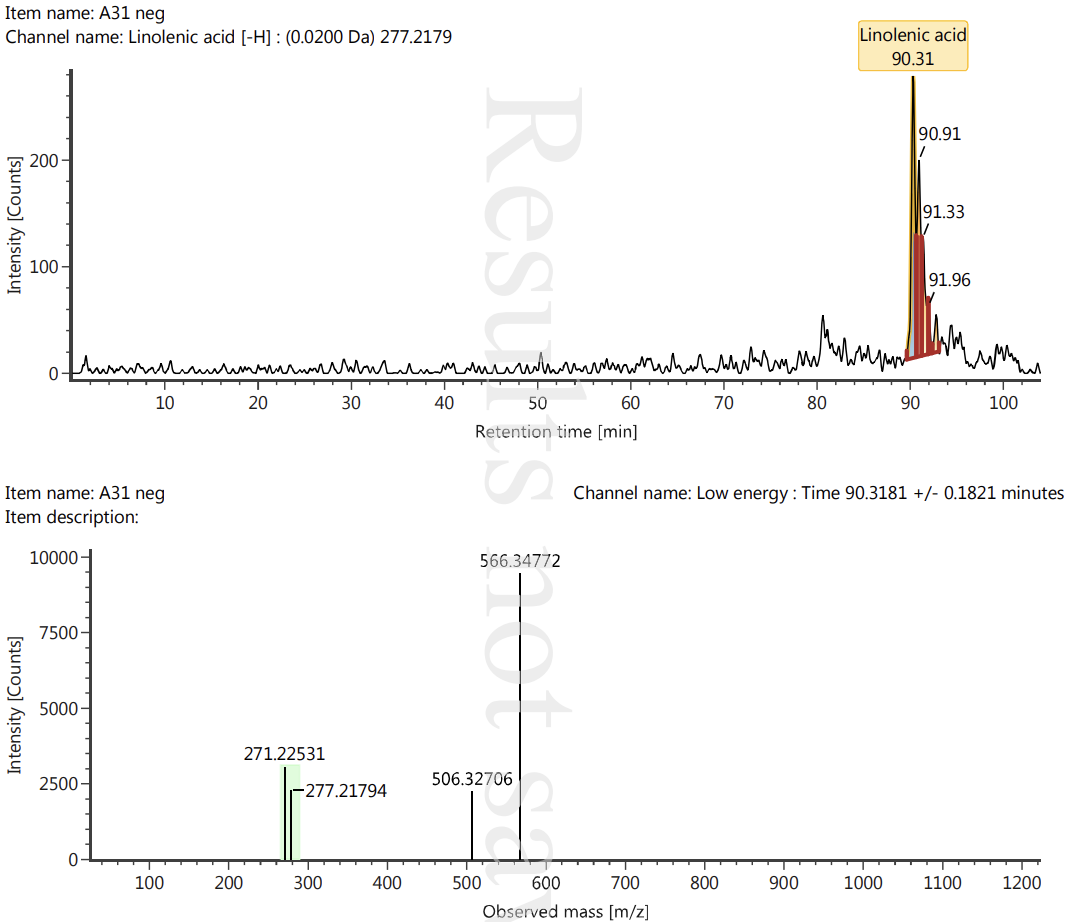

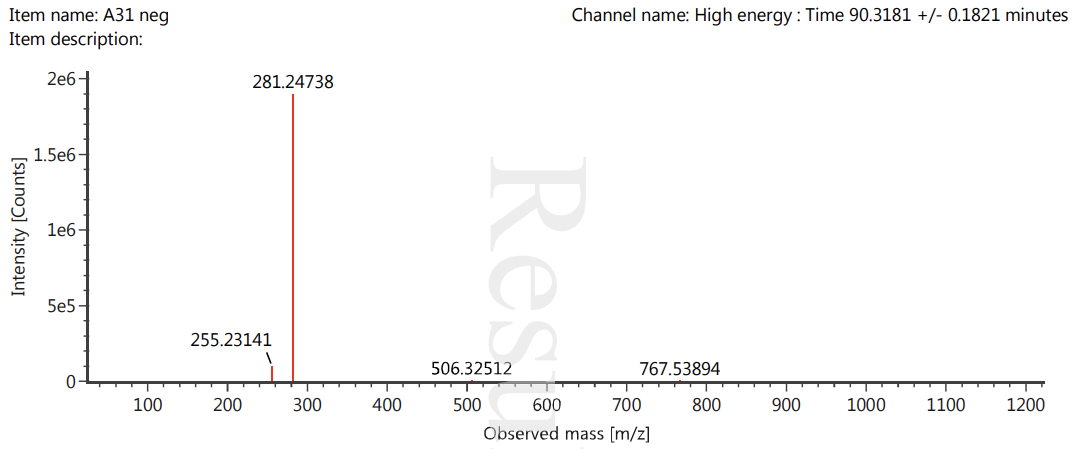


**A52**

给药后2小时样本

**Ginsenoside Rb1**

Neutral mass (Da) 1108.60294

Observed neutral mass (Da) 1108.607

Observed m/z 1107.5997

Mass error (mDa) 4

Mass error (ppm) 3.6

Expected RT (min) 0

Observed RT (min) 86.94

Detector counts 66447

Response 48316

Adducts -H


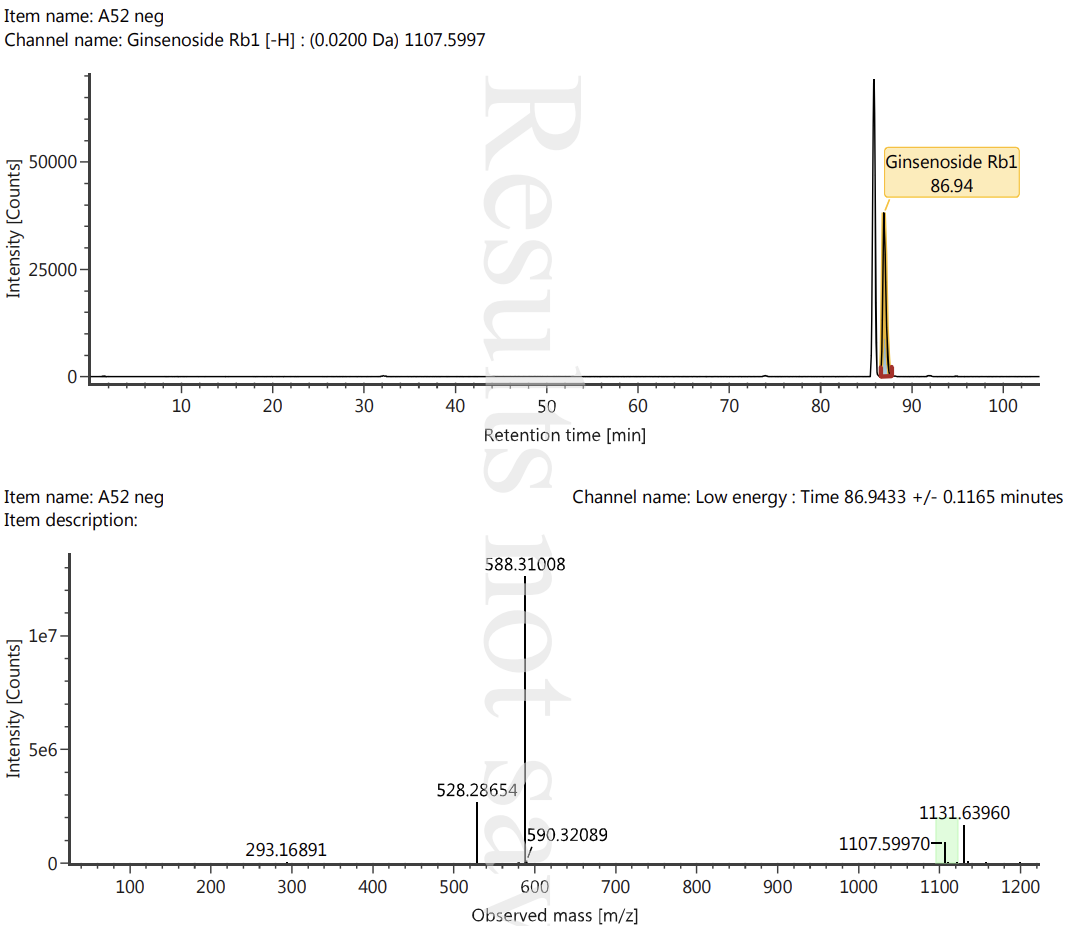


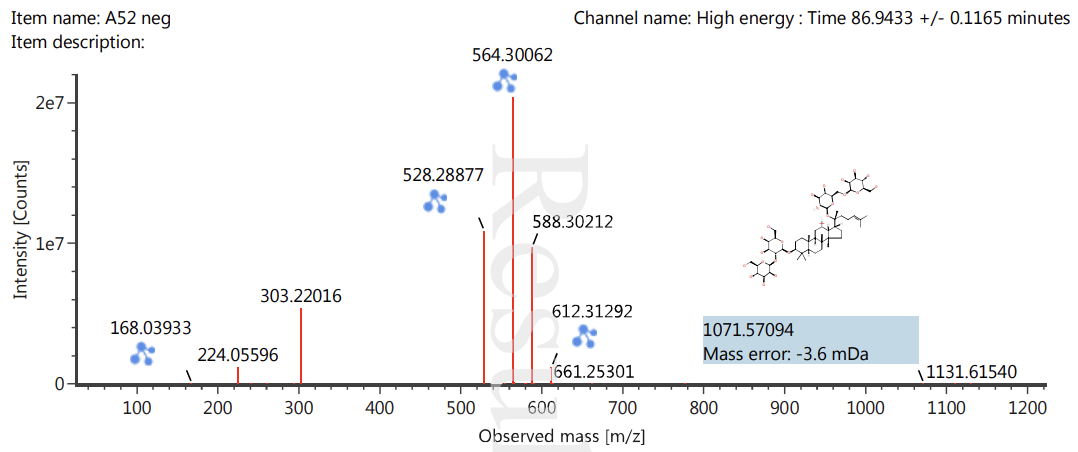


**20(R)-notoginsenoside R2**

Neutral mass (Da) 770.48164

Observed neutral mass (Da) 770.4766

Observed m/z 815.4748

Mass error (mDa) -5.1

Mass error (ppm) -6.2

Expected RT (min) 0

Observed RT (min) 74.31

Detector counts 30212

Response 28560

Adducts +HCOO


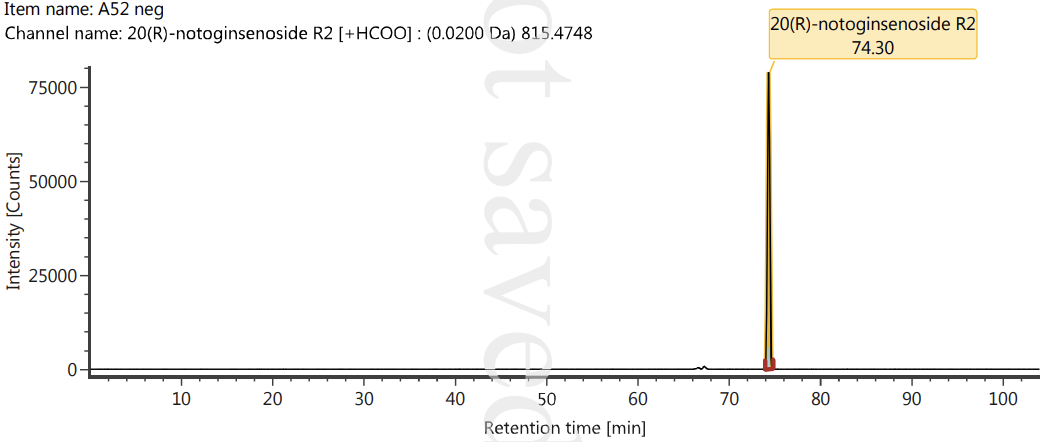


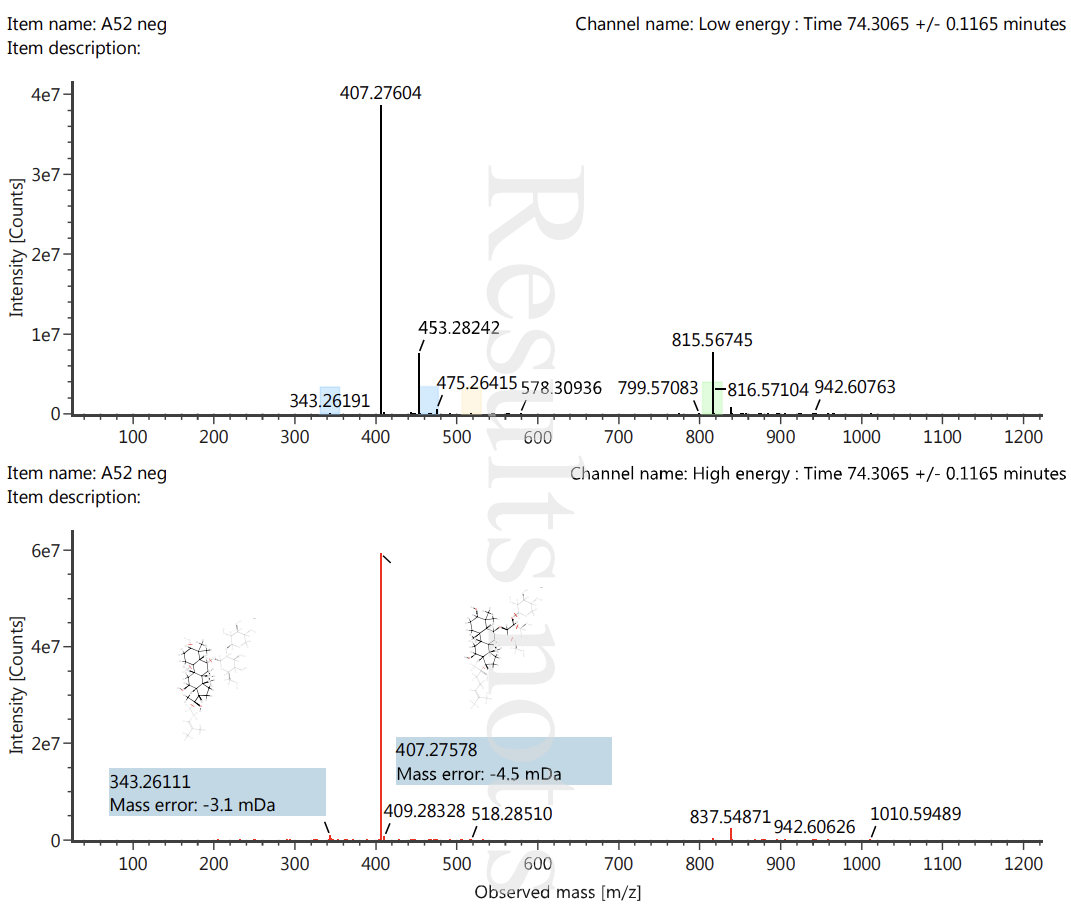


**Methyl palmitate**

Neutral mass (Da) 270.25588 270.25588

Observed neutral mass (Da) 270.2535 270.2534

Observed m/z 315.2517 315.2516

Mass error (mDa) -2.4 -2.5

Mass error (ppm) -7.7 -7.9

Expected RT (min) 0 0

Observed RT (min) 78.8 76.71

Detector counts 20715 6030

Response 20009 5692

Adducts +HCOO +HCOO


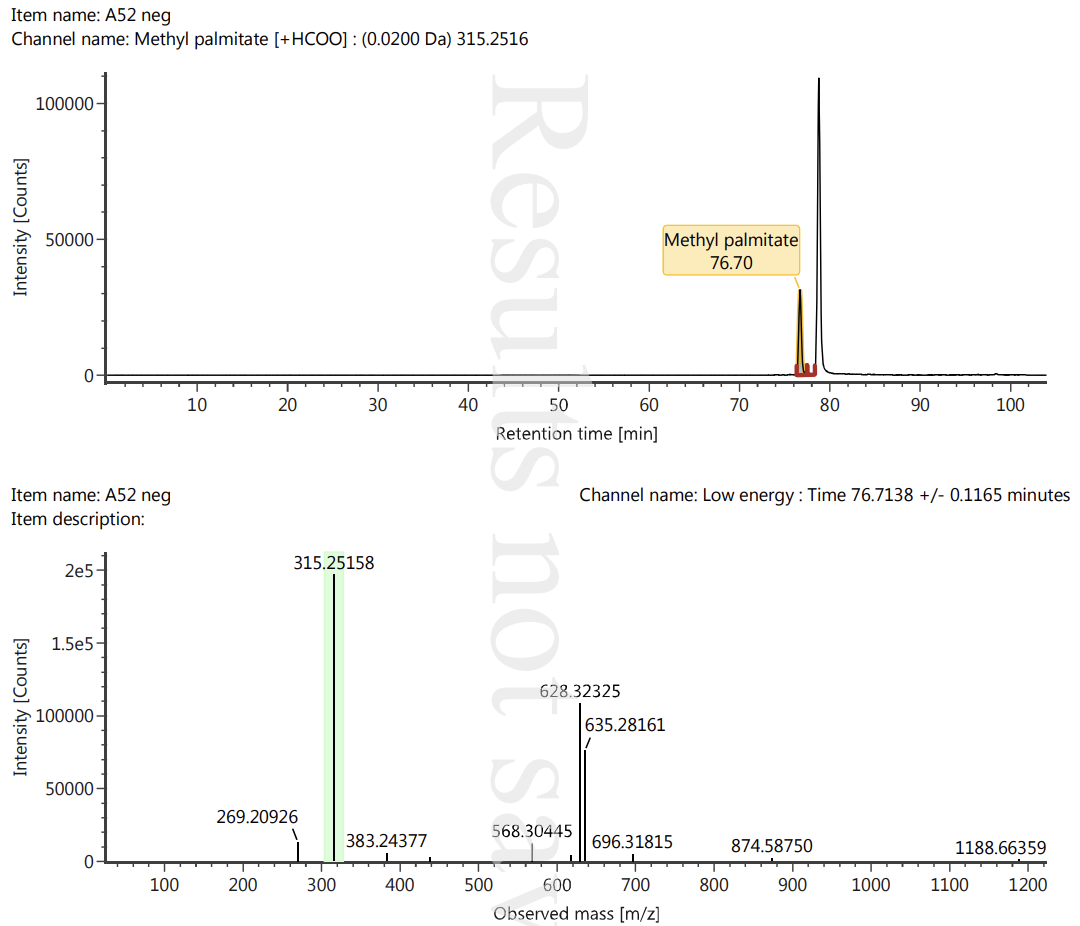


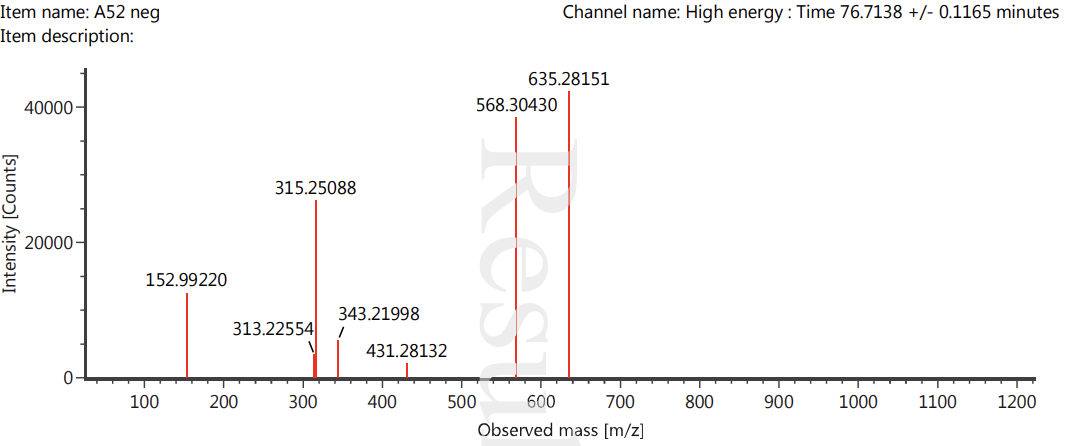


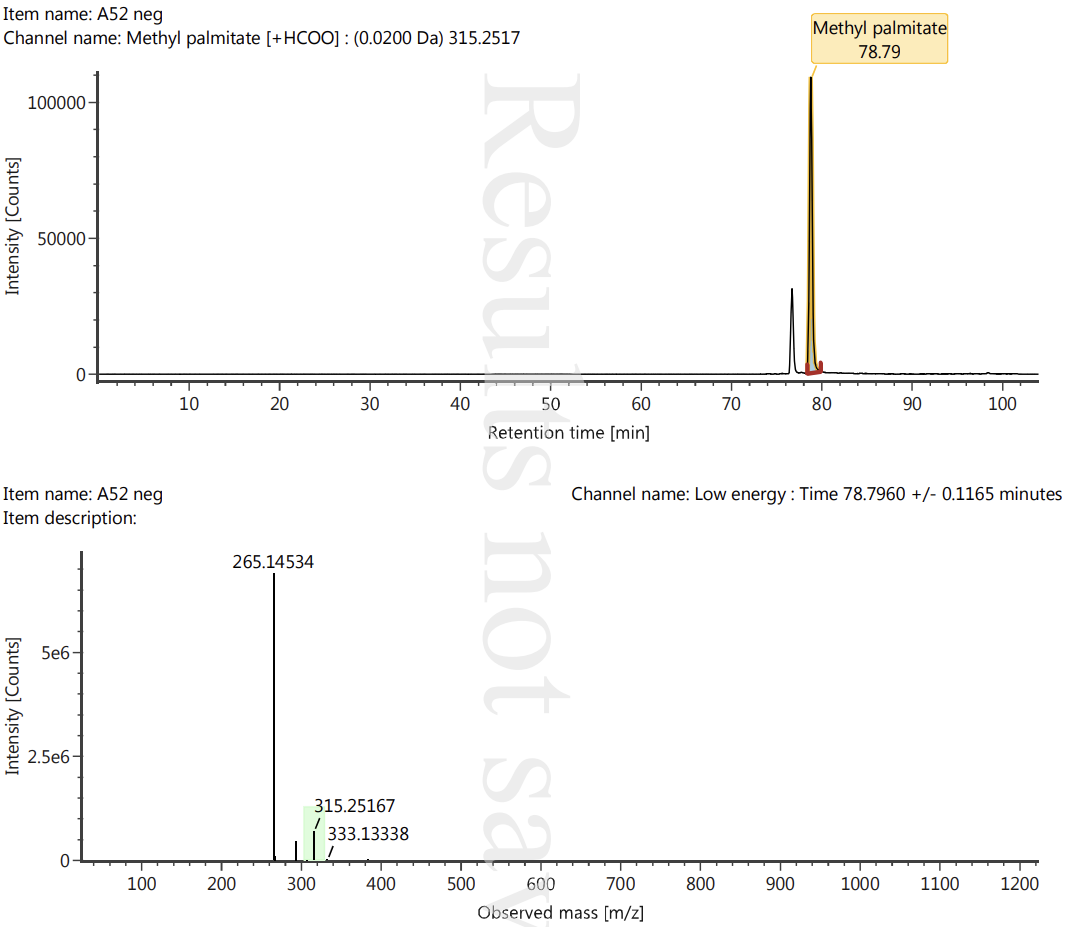


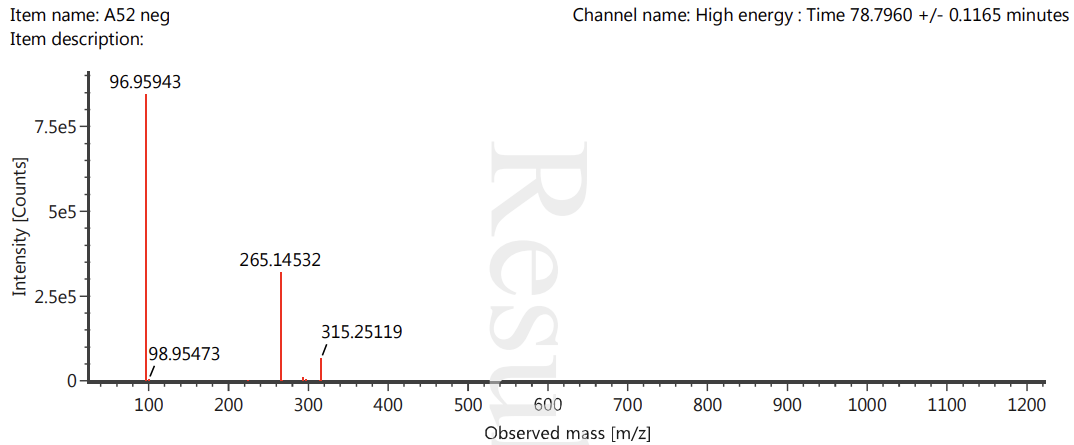


**Astragaloside III**

Neutral mass (Da) 784.46091

Observed neutral mass (Da) 784.4629

Observed m/z 829.4611

Mass error (mDa) 1.9

Mass error (ppm) 2.3

Expected RT (min) 0

Observed RT (min) 73.25

Detector counts 45250

Response 199

Adducts +HCOO


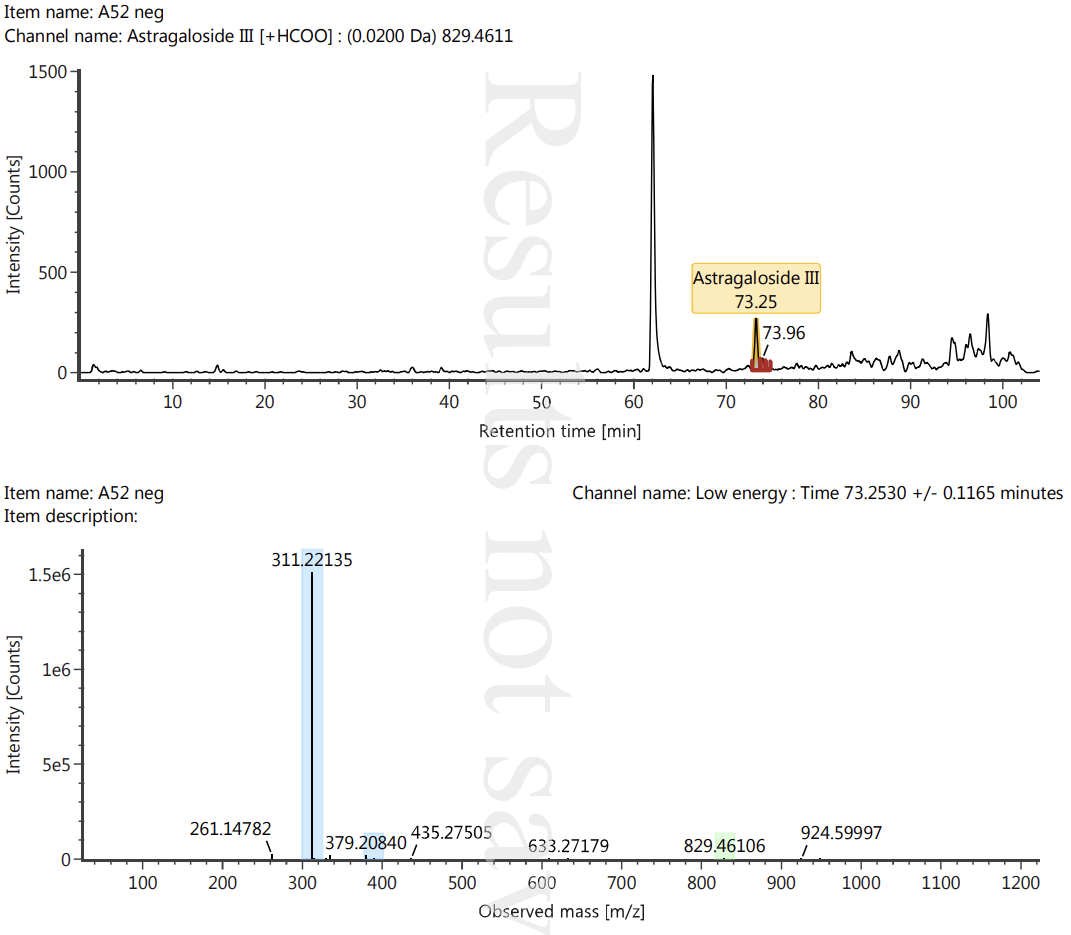

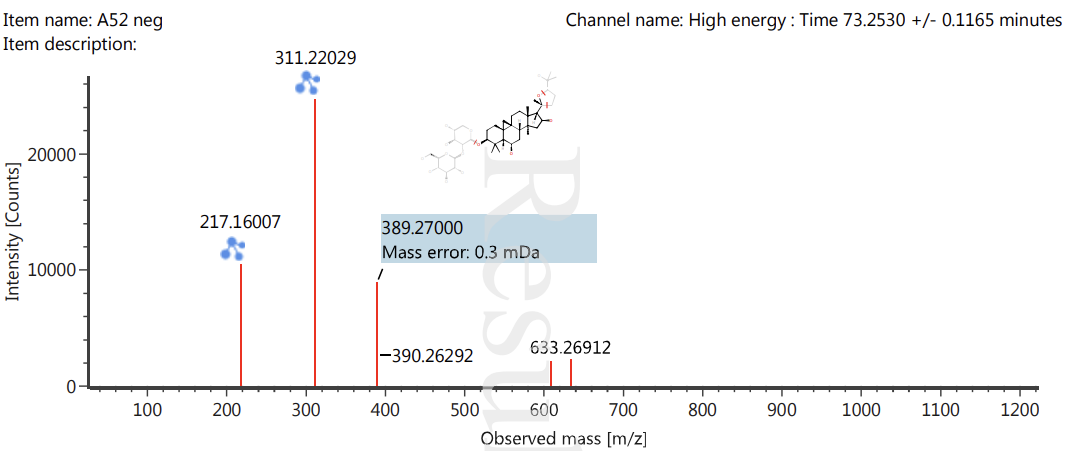


Ginsenoside Rb3

Neutral mass (Da)

Observed neutral mass (Da)

Observed m/z

Mass error (mDa)

Mass error (ppm)

Expected RT (min)

Observed RT (min)

Detector counts

Response

Adducts

1078.59237 1078.5938 1123.592 1.5 1.3 0 70.8 7191 89 +HCOO


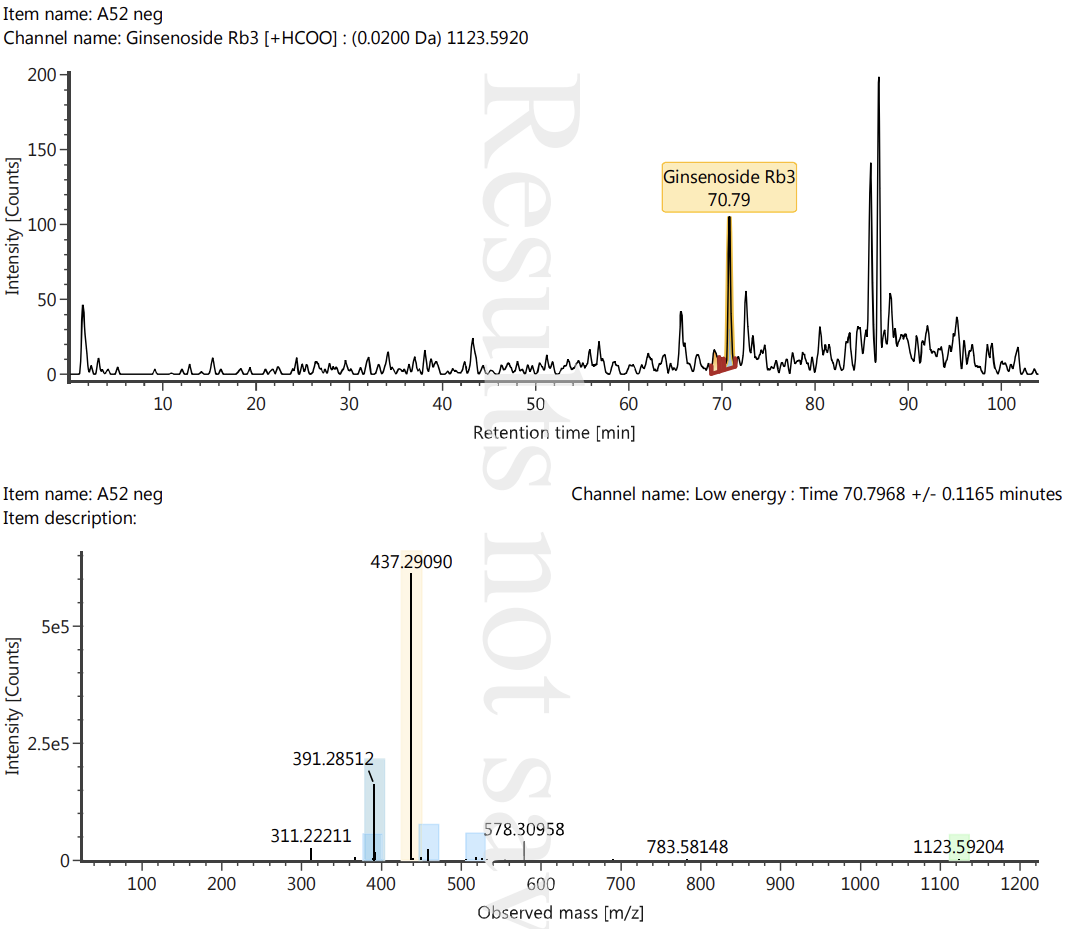

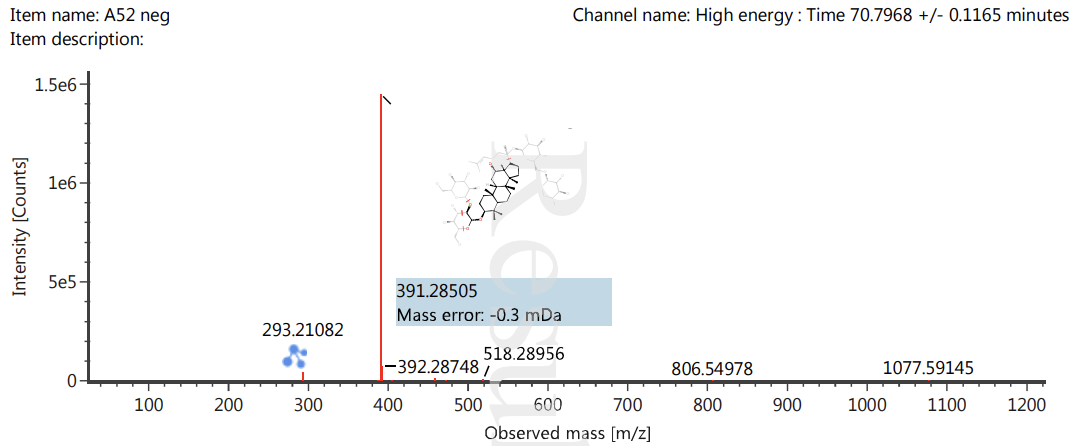


Ginsenoside Rg10

Neutral mass (Da)

Observed neutral mass (Da)

Observed m/z

Mass error (mDa)

Mass error (ppm)

Expected RT (min)

Observed RT (min)

Detector counts

Response

Adducts

781.47382 781.4709 780.4636 -2.9 -3.8 0 99.03 66 66 -H


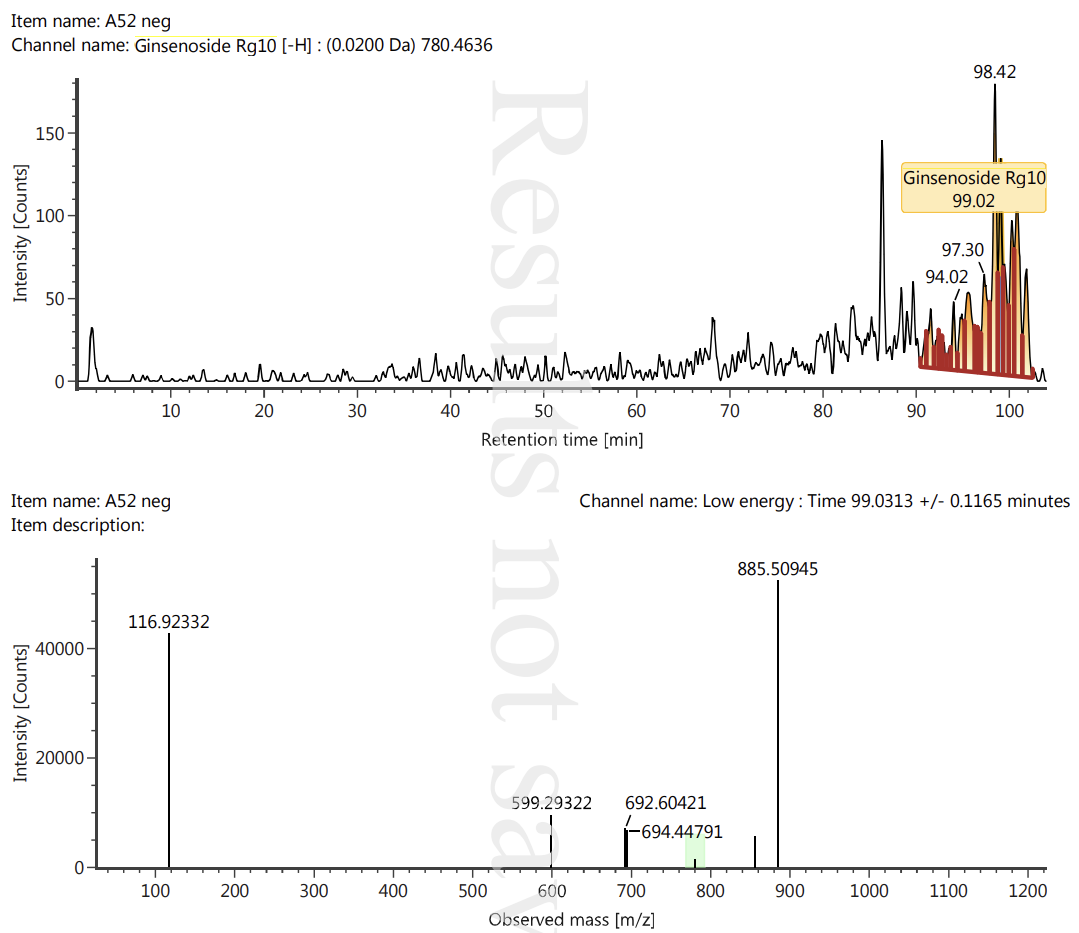


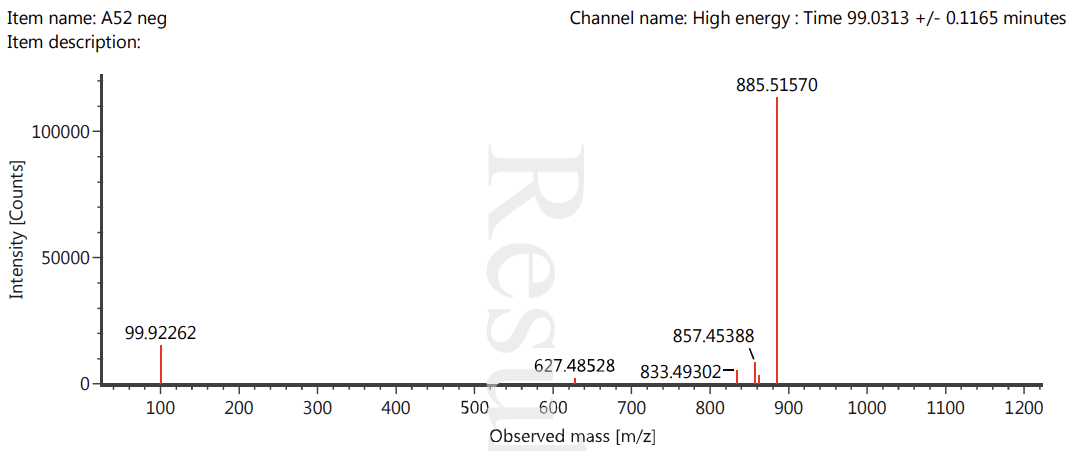


Malonyl-ginsenoside Rd6

Neutral mass (Da)

Observed neutral mass (Da)

Observed m/z

Mass error (mDa)

Mass error (ppm)

Expected RT (min)

Observed RT (min)

Detector counts

Response

Adducts

1119.55873 1119.5698 1118.5625 11 9.9 0 36.5 41 41 -H


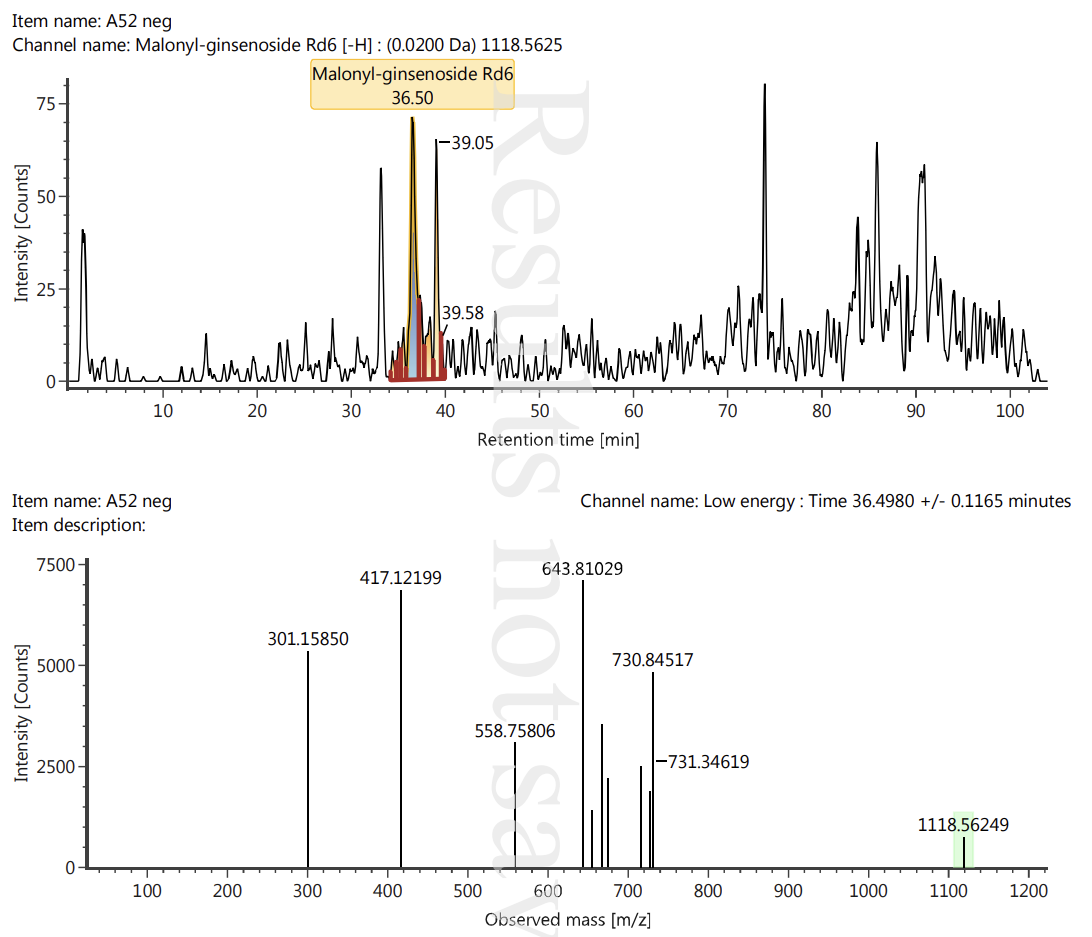


**A73**

给药后4小时样本

Ginsenoside Rb1

Neutral mass (Da)

Observed neutral mass (Da)

Observed m/z

Mass error (mDa)

Mass error (ppm)

Expected RT (min)

Observed RT (min)

Detector counts

Response

Adducts

1108.60294 1108.6038 1153.602 0.8 0.7 0 70.21 172 108 +HCOO

1108.60294 1108.5984 1107.5912 -4.5 -4.1 0 86.97 1521234 43971 -H
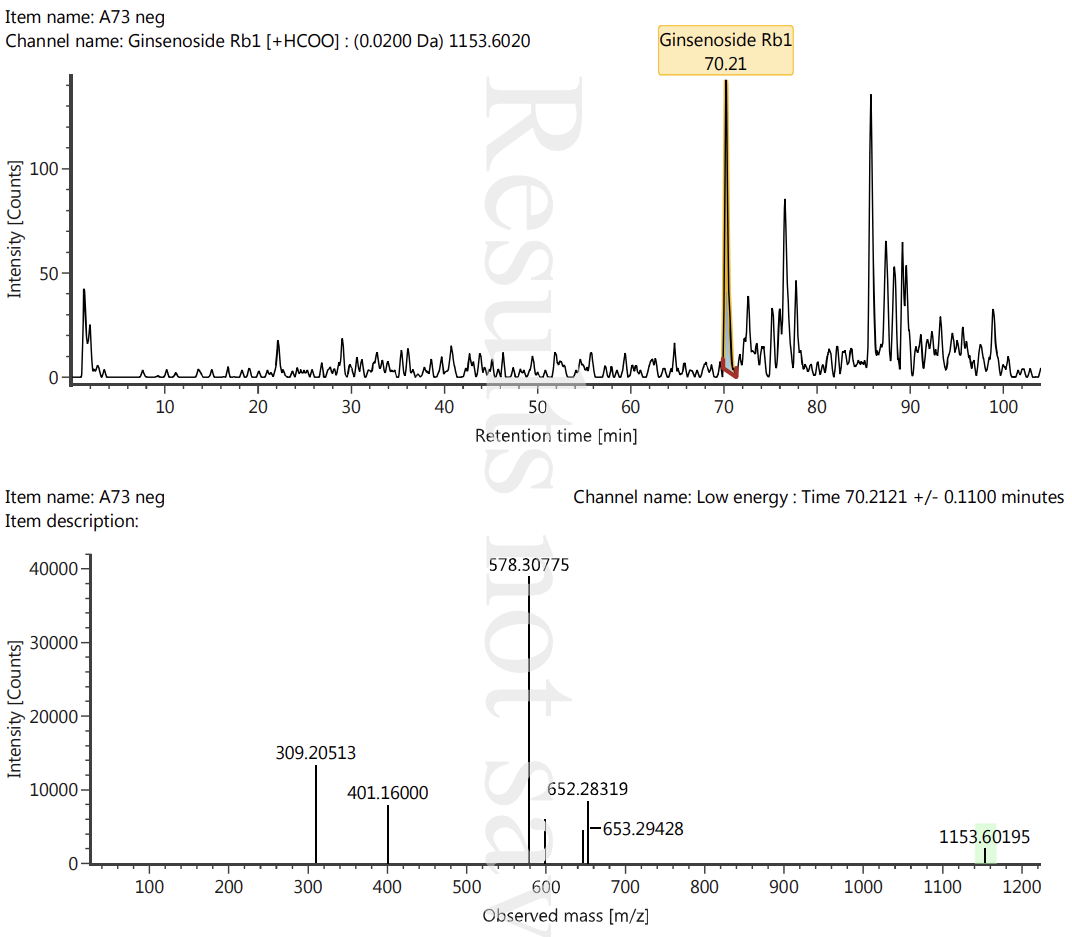

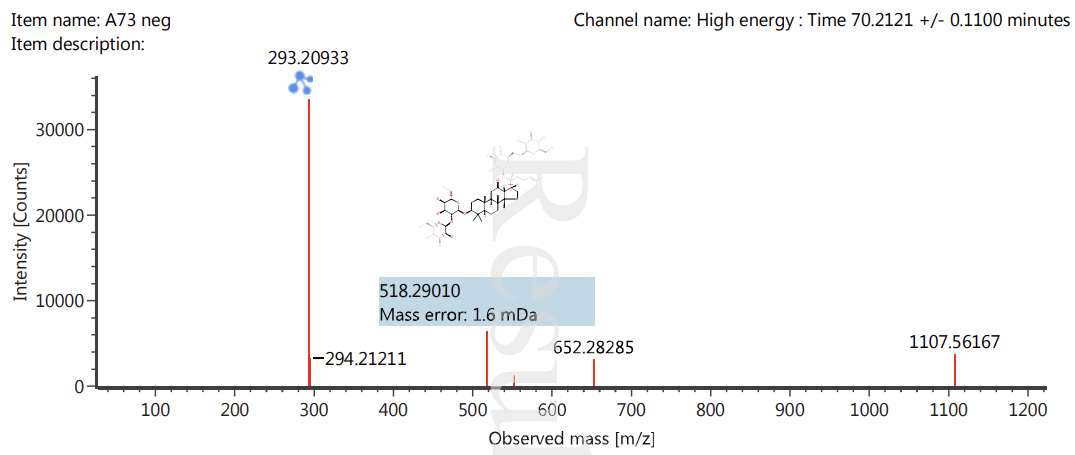


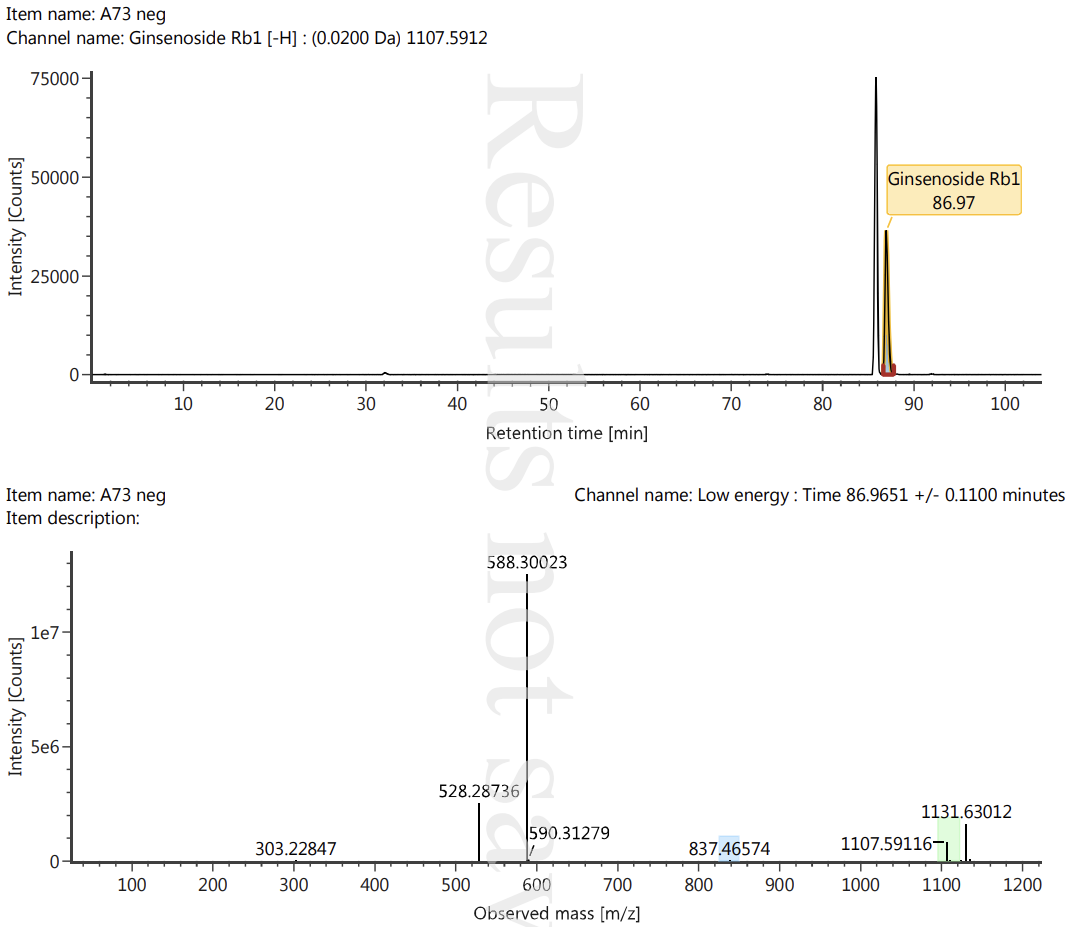


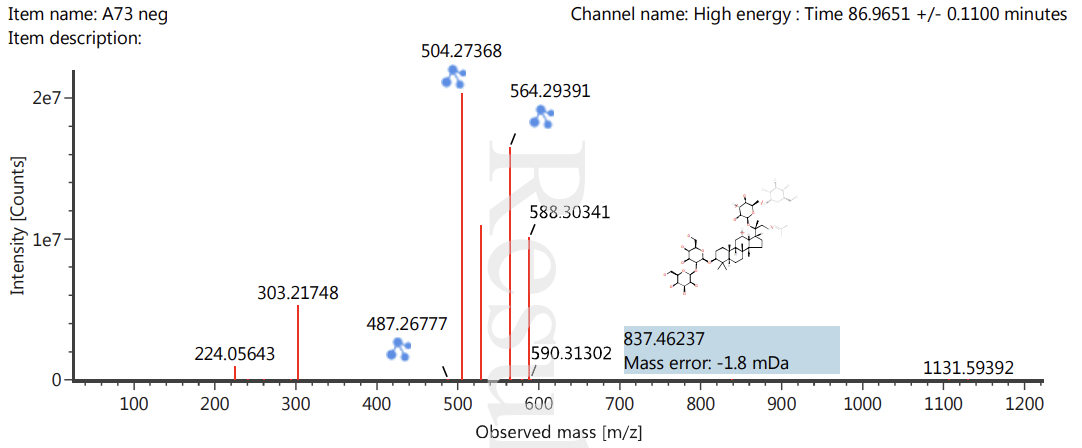


Ginsenoside Ki

Neutral mass (Da)

Observed neutral mass (Da)

Observed m/z

Mass error (mDa)

Mass error (ppm)

Expected RT (min)

Observed RT (min)

Detector counts

Response

Adducts

654.4343 654.4313 653.4241 -3 -4.5 0 92.37 441 441 -H

ASTRAGALOSIDE I

Neutral mass (Da)

Observed neutral mass (Da)

Observed m/z

Mass error (mDa)

Mass error (ppm)

Expected RT (min)

Observed RT (min)

Detector counts

Response

Adducts

840.45074 840.4457 885.4439 -5 -5.6 0 62.14 265 265 +HCOO

Astragaloside III

Neutral mass (Da)

Observed neutral mass (Da)

Observed m/z

Mass error (mDa)

Mass error (ppm)

Expected RT (min)

Observed RT (min)

Detector counts

Response

Adducts

784.46091 784.4648 829.463 3.8 4.6 0 73.29 10970 237 +HCOO

Floralginsenoside H

Neutral mass (Da)

Observed neutral mass (Da)

Observed m/z

Mass error (mDa)

Mass error (ppm)

Expected RT (min)

Observed RT (min)

Detector counts

Response

Adducts

1020.55051 1020.5408 1019.5336 -9.7 -9.5 0 87.23 196 196 -H

Ginsenoside Rs1

Neutral mass (Da)

Observed neutral mass (Da)

Observed m/z

Mass error (mDa)

Mass error (ppm)

Expected RT (min)

Observed RT (min)

Detector counts

Response

Adducts

1120.60294 1120.5989 1165.5971 -4.1 -3.5 0 85.94 751017 149 +HCOO

Ginsenoside Rb2

Neutral mass (Da)

Observed neutral mass (Da)

Observed m/z

Mass error (mDa)

Mass error (ppm)

Expected RT (min)

Observed RT (min)

Detector counts

Response

Adducts

Ginsenoside Rb2 Identified 1078.59237 1078.5917 1123.5899 -0.7 -0.6 0 72.55 78 78 +HCOO

Malonyl-ginsenoside Rb2

Neutral mass (Da)

Observed neutral mass (Da)

Observed m/z

Mass error (mDa)

Mass error (ppm)

Expected RT (min)

Observed RT (min)

Detector counts

Response

Adducts

1164.59277 1164.6008 1163.5935 8 6.9 0 93.2 96 96 -H

**A96**

给药后6小时样本

ASTRAGALOSIDE I

Neutral mass (Da)

Observed neutral mass (Da)

Observed m/z

Mass error (mDa)

Mass error (ppm)

Expected RT (min)

Observed RT (min)

Detector counts

Response

Adducts

840.45074 840.4491 885.4473 -1.7 -1.9 0 89.01 1486 1486 +HCOO

840.45074 840.4509 885.4491 0.2 0.2 0 62.11 426 325 +HCOO

Methyl palmitate

Neutral mass (Da)

Observed neutral mass (Da)

Observed m/z

Mass error (mDa)

Mass error (ppm)

Expected RT (min)

Observed RT (min)

Detector counts

Response

Adducts

Methyl palmitate Identified 270.25588 270.253 315.2512 -2.9 -9.3 0 76.73 821 821 +HCOO

Ginsenoside Rb1

Neutral mass (Da)

Observed neutral mass (Da)

Observed m/z

Mass error (mDa)

Mass error (ppm)

Expected RT (min)

Observed RT (min)

Detector counts

Response

Adducts

1108.60294 1108.5952 1153.5934 -7.7 -6.7 0 70.2 1064 599 +HCOO

1108.60294 1108.605 1153.6032 2 1.8 0 85.74 145940 93 +HCOO

Ginsenoside Rb3

Neutral mass (Da)

Observed neutral mass (Da)

Observed m/z

Mass error (mDa)

Mass error (ppm)

Expected RT (min)

Observed RT (min)

Detector counts

Response

Adducts

1078.59237 1078.5943 1123.5925 2 1.8 0 70.8 2820 328 +HCOO

1078.59237 1078.5859 1123.5841 -6.4 -5.7 0 85.91 107398 315 +HCOO

Astragaloside III

Neutral mass (Da)

Observed neutral mass (Da)

Observed m/z

Mass error (mDa)

Mass error (ppm)

Expected RT (min)

Observed RT (min)

Detector counts

Response

Adducts

784.46091 784.4662 829.4644 5.3 6.3 0 73.24 3479 190 +HCOO

Floralginsenoside F

Neutral mass (Da)

Observed neutral mass (Da)

Observed m/z

Mass error (mDa)

Mass error (ppm)

Expected RT (min)

Observed RT (min)

Detector counts

Response

Adducts

816.48712 816.4953 861.4935 8.2 9.5 0 96.02 187 187 +HCOO

**A118**

给药后8小时样本

Formononetin

Neutral mass (Da)

Observed neutral mass (Da)

Observed m/z

Mass error (mDa)

Mass error (ppm)

Expected RT (min)

Observed RT (min)

Detector counts

Response

Adducts

268.07356 268.0755 267.0683 2 7.4 0 3.54 63065 62198 -H

Ginsenoside Rb1

Neutral mass (Da)

Observed neutral mass (Da)

Observed m/z

Mass error (mDa)

Mass error (ppm)

Expected RT (min)

Observed RT (min)

Detector counts

Response

Adducts

1108.60294 1108.5972 1107.59 -5.7 -5.1 0 87.04 236176 47522 -H

1108.60294 1108.5983 1153.5966 -4.6 -4 0 70.22 222 98 +HCOO

Ginsenoside Rb3Neutral mass (Da)

Observed neutral mass (Da)

Observed m/z

Mass error (mDa)

Mass error (ppm)

Expected RT (min)

Observed RT (min)

Detector counts

Response

Adducts

1078.59237 1078.5818 1077.5746 -10.5 -9.8 0 90.43 2246 1127 -H

Ginsenoside Ra9

Neutral mass (Da)

Observed neutral mass (Da)

Observed m/z

Mass error (mDa)

Mass error (ppm)

Expected RT (min)

Observed RT (min)

Detector counts

Response

Adducts

Ginsenoside Ra9

1146.61859 1146.626 1145.6187 7.4 6.4 0 86.83 194 194 -H

Ginsenoside Ki

Neutral mass (Da)

Observed neutral mass (Da)

Observed m/z

Mass error (mDa)

Mass error (ppm)

Expected RT (min)

Observed RT (min)

Detector counts

Response

Adducts

Ginsenoside Ki Identified 654.4343 654.4343 653.427 0 0 0 92.39 571 571 -H

Malonyl-ginsenoside Rb2

Neutral mass (Da)

Observed neutral mass (Da)

Observed m/z

Mass error (mDa)

Mass error (ppm)

Expected RT (min)

Observed RT (min)

Detector counts

Response

Adducts

1164.59277 1164.5957 1163.5884 2.9 2.5 0 86.45 169 169 -H

20(R)-notoginsenoside R2

Neutral mass (Da)

Observed neutral mass (Da)

Observed m/z

Mass error (mDa)

Mass error (ppm)

Expected RT (min)

Observed RT (min)

Detector counts

Response

Adducts

20(R)-notoginsenoside R2

770.48164 770.4784 815.4766 -3.2 -4 0 74.33 10297 8892 +HCOO

Astragaloside III

Neutral mass (Da)

Observed neutral mass (Da)

Observed m/z

Mass error (mDa)

Mass error (ppm)

Expected RT (min)

Observed RT (min)

Detector counts

Response

Adducts

784.46091 784.4678 829.466 6.9 8.3 0 73.29 518 232 +HCOO

Astraganoside

Neutral mass (Da)

Observed neutral mass (Da)

Observed m/z

Mass error (mDa)

Mass error (ppm)

Expected RT (min)

Observed RT (min)

Detector counts

Response

Adducts

480.16316 480.1616 479.1543 -1.6 -3.2 0 67.67 52 52 -H

Astraisoflavan-7--O--β-D-glucoside

Neutral mass (Da)

Observed neutral mass (Da)

Observed m/z

Mass error (mDa)

Mass error (ppm)

Expected RT (min)

Observed RT (min)

Detector counts

Response

Adducts

464.16825 464.169 463.1617 0.8 1.6 0 52.26 67 67 -H

Majoroside F6

Neutral mass (Da)

Observed neutral mass (Da)

Observed m/z

Mass error (mDa)

Mass error (ppm)

Expected RT (min)

Observed RT (min)

Detector counts

Response

Adducts

948.52938 948.5254 947.5182 -3.9 -4.2 0 89.57 13109 989 -H

Floralginsenoside H

Neutral mass (Da)

Observed neutral mass (Da)

Observed m/z

Mass error (mDa)

Mass error (ppm)

Expected RT (min)

Observed RT (min)

Detector counts

Response

Adducts

1020.55051 1020.5465 1019.5392 -4.1 -4 0 87.24 511 511 -H

Floralginsenoside Td

Neutral mass (Da)

Observed neutral mass (Da)

Observed m/z

Mass error (mDa)

Mass error (ppm)

Expected RT (min)

Observed RT (min)

Detector counts

Response

Adducts

1110.5822 1110.591 1109.5837 8.8 7.9 0 89.74 355 355 -H

Floralginsenoside P

Neutral mass (Da)

Observed neutral mass (Da)

Observed m/z

Mass error (mDa)

Mass error (ppm)

Expected RT (min)

Observed RT (min)

Detector counts

Response

Adducts

1094.58729 1094.5806 1139.5788 -6.7 -5.9 0 86.41 174 174 +HCOO

**A1312**

给药后12小时样本

Methyl palmitate

Neutral mass (Da)

Observed neutral mass (Da)

Observed m/z

Mass error (mDa)

Mass error (ppm)

Expected RT (min)

Observed RT (min)

Detector counts

Response

Adducts

270.25588 270.2574 315.2556 1.5 4.8 0 78.88 17959 17229 +HCOO

270.25588 270.2567 315.2549 0.8 2.6 0 76.81 1064 958 +HCOO

Isoastragaloside I Neutral mass (Da)

Observed neutral mass (Da)

Observed m/z

Mass error (mDa)

Mass error (ppm)

Expected RT (min)

Observed RT (min)

Detector counts

Response

Adducts

868.48204 868.4905 913.4887 8.5 9.3 0 98.91 150 150 +HCOO

**A1424**

给药后24小时样本

Methyl palmitate

Neutral mass (Da)

Observed neutral mass (Da)

Observed m/z

Mass error (mDa)

Mass error (ppm)

Expected RT (min)

Observed RT (min)

Detector counts

Response

Adducts

270.25588 270.2545 315.2527 -1.4 -4.5 0 78.91 21190 20357 +HCOO

270.25588 270.2528 315.251 -3.1 -9.9 0 76.84 1137 1032 +HCOO

Ginsenoside Rb3

Neutral mass (Da)

Observed neutral mass (Da)

Observed m/z

Mass error (mDa)

Mass error (ppm)

Expected RT (min)

Observed RT (min)

Detector counts

Response

Adducts

Ginsenoside Rb3 Identified 1078.59237 1078.59 1077.5827 -2.3 -2.2 0 90.57 1096802 1160 -H

Ginsenoside Rb1Neutral mass (Da)

Observed neutral mass (Da)

Observed m/z

Mass error (mDa)

Mass error (ppm)

Expected RT (min)

Observed RT (min)

Detector counts

Response

Adducts

Ginsenoside Rb1 Identified 1108.60294 1108.6141 1153.6123 11.2 9.7 0 70.31 53 53 +HCOO

ASTRAGALOSIDE I

Neutral mass (Da)

Observed neutral mass (Da)

Observed m/z

Mass error (mDa)

Mass error (ppm)

Expected RT (min)

Observed RT (min)

Detector counts

Response

Adducts

840.45074 840.4497 885.4479 -1 -1.2 0 62.18 345 244 +HCOO

Astragaloside A

Neutral mass (Da)

Observed neutral mass (Da)

Observed m/z

Mass error (mDa)

Mass error (ppm)

Expected RT (min)

Observed RT (min)

Detector counts

Response

Adducts

784.46091 784.4646 829.4628 3.7 4.4 0 73.35 110 110 +HCOO

Notoginsenoside Rt

Neutral mass (Da)

Observed neutral mass (Da)

Observed m/z

Mass error (mDa)

Mass error (ppm)

Expected RT (min)

Observed RT (min)

Detector counts

Response

Adducts

842.50277 842.5104 887.5086 7.6 8.6 0 95.75 149 149 +HCOO

Floralginsenoside H

Neutral mass (Da)

Observed neutral mass (Da)

Observed m/z

Mass error (mDa)

Mass error (ppm)

Expected RT (min)

Observed RT (min)

Detector counts

Response

Adducts

1020.55051 1020.5557 1019.5484 5.2 5.1 0 87.33 655 655 -H

1020.55051 1020.558 1019.5507 7.5 7.3 0 87.35 632 632 -H
